# Reverse vaccinology assisted designing of multiepitope-based subunit vaccine against SARS-CoV-2

**DOI:** 10.1186/s40249-020-00752-w

**Published:** 2020-09-16

**Authors:** Muhammad Tahir ul Qamar, Farah Shahid, Sadia Aslam, Usman Ali Ashfaq, Sidra Aslam, Israr Fatima, Muhammad Mazhar Fareed, Ali Zohaib, Ling-Ling Chen

**Affiliations:** 1 College of Life Science and Technology, Guangxi University, Nanning, P. R. China; 2 Department of Bioinformatics and Biotechnology, Government College University Faisalabad (GCUF), Faisalabad, Pakistan; 3 Jinnah Hospital, Lahore, Pakistan; 4 Department of Healthcare Biotechnology, Atta-ur-Rahman School of Applied Biosciences (ASAB), National University of Sciences and Technology (NUST), Islamabad, Pakistan

**Keywords:** SARS-CoV-2, COVID-19, Structural protein, Epitope, Vaccine, Multiepitope-based subunit vaccine, Immunoinformatics

## Abstract

**Background:**

Coronavirus disease 2019 (COVID-19) linked with severe acute respiratory syndrome coronavirus 2 (SARS-CoV-2) cause severe illness and life-threatening pneumonia in humans. The current COVID-19 pandemic demands an effective vaccine to acquire protection against the infection. Therefore, the present study was aimed to design a multiepitope-based subunit vaccine (MESV) against COVID-19.

**Methods:**

Structural proteins (Surface glycoprotein, Envelope protein, and Membrane glycoprotein) of SARS-CoV-2 are responsible for its prime functions. Sequences of proteins were downloaded from GenBank and several immunoinformatics coupled with computational approaches were employed to forecast B- and T- cell epitopes from the SARS-CoV-2 highly antigenic structural proteins to design an effective MESV.

**Results:**

Predicted epitopes suggested high antigenicity, conserveness, substantial interactions with the human leukocyte antigen (HLA) binding alleles, and collective global population coverage of 88.40%. Taken together, 276 amino acids long MESV was designed by connecting 3 cytotoxic T lymphocytes (CTL), 6 helper T lymphocyte (HTL) and 4 B-cell epitopes with suitable adjuvant and linkers. The MESV construct was non-allergenic, stable, and highly antigenic. Molecular docking showed a stable and high binding affinity of MESV with human pathogenic toll-like receptors-3 (TLR3). Furthermore, in silico immune simulation revealed significant immunogenic response of MESV. Finally, MEV codons were optimized for its in silico cloning into the *Escherichia coli* K-12 system, to ensure its increased expression.

**Conclusion:**

The MESV developed in this study is capable of generating immune response against COVID-19. Therefore, if designed MESV further investigated experimentally, it would be an effective vaccine candidate against SARS-CoV-2 to control and prevent COVID-19.

## Background

Viruses have the potential to become dangerous life threat and cause irreparable loss to human beings. Hardly the world learns to cope with one strain of virus when another emerges and poses a threat to the future of humanity. A similar situation has emerged when a new strain of novel coronavirus (CoV) that has not been previously identified in humans reported in December, 2019 [1, 2]. Coronaviruses are the largest among RNA viruses belonging to Coronaviridae, Roniviridae and Arteriviridae families. Coronaviridae are unsegmented, *3*′ polyadenylated and *5*′ capped positive sense single-stranded RNA viruses cause various respiratory diseases in humans [2, 3]. CoVs are classified into four classes: alpha, beta, delta, and gamma. Amongst them, beta and alpha CoVs have been reported for infecting humans [4]. Recent CoV strain has received tremendous attention from researchers, as it causes a global pandemic of coronavirus disease 2019 (COVID-19) [5]. Severe acute respiratory syndrome coronavirus 2 (SARS-CoV-2) was identified as the causative agent of this pandemic [6]. The study of genome sequences has cast a shadow that SARS-CoV-2 is closely related to the SARS-CoV which is the causative agent of the SARS disease in 2002/2003 [7]. Initial diagnostic procedures indicated that the SARS-CoV-2 is primarily spread through respiratory droplets from sneezing/coughing, body contact and to some extent through fecal contact [8]. The SARS-CoV-2 may show symptoms within 14 days after exposure, or in some cases it takes more than 14 days. Symptoms of patients infected with COVID-19 include fever, runny nose, cough, and dyspnea [9]. Although the entire genome sequence of the virus has been published, the origin and proliferation mechanism of the new coronavirus is still ambiguous as stated by the World Health Organization [10]. Initial reports claimed that bats, snakes, pangolins or civet could be a possible animal source, but the claims are under debate and needs substantial research to prove it [6, 11–13]. Researchers are currently working to sort out the SARS-CoV-2 source, including possible intermediate animal vectors.

The samples taken from a respiratory system-throat swab or lung fluid are helpful in diagnosing its infection in patients [14]. A special clinical diagnostic reverse transcription-PCR based test was developed [15]. Over 200 clinical trials are currently underway to test new and repurposed compounds against SARS-CoV-2 [16, 17]. Several medications such as hydroxychloroquine, remedesivir, and dexamethasone are being tested in clinical trials [18–21]. Several vaccines including subunit vaccines [18, 22], nano-particle based vaccines, viral vector vaccines (adenovirus vector, Ankara vector), inactivated vaccines, fusion-protein based vaccines, recombinant protein, DNA vaccines, and live-attenuated vaccines are also being developed and in pre-clinical trials, but these vaccines are long months away from the market [23–27].

**Fig. 1:**
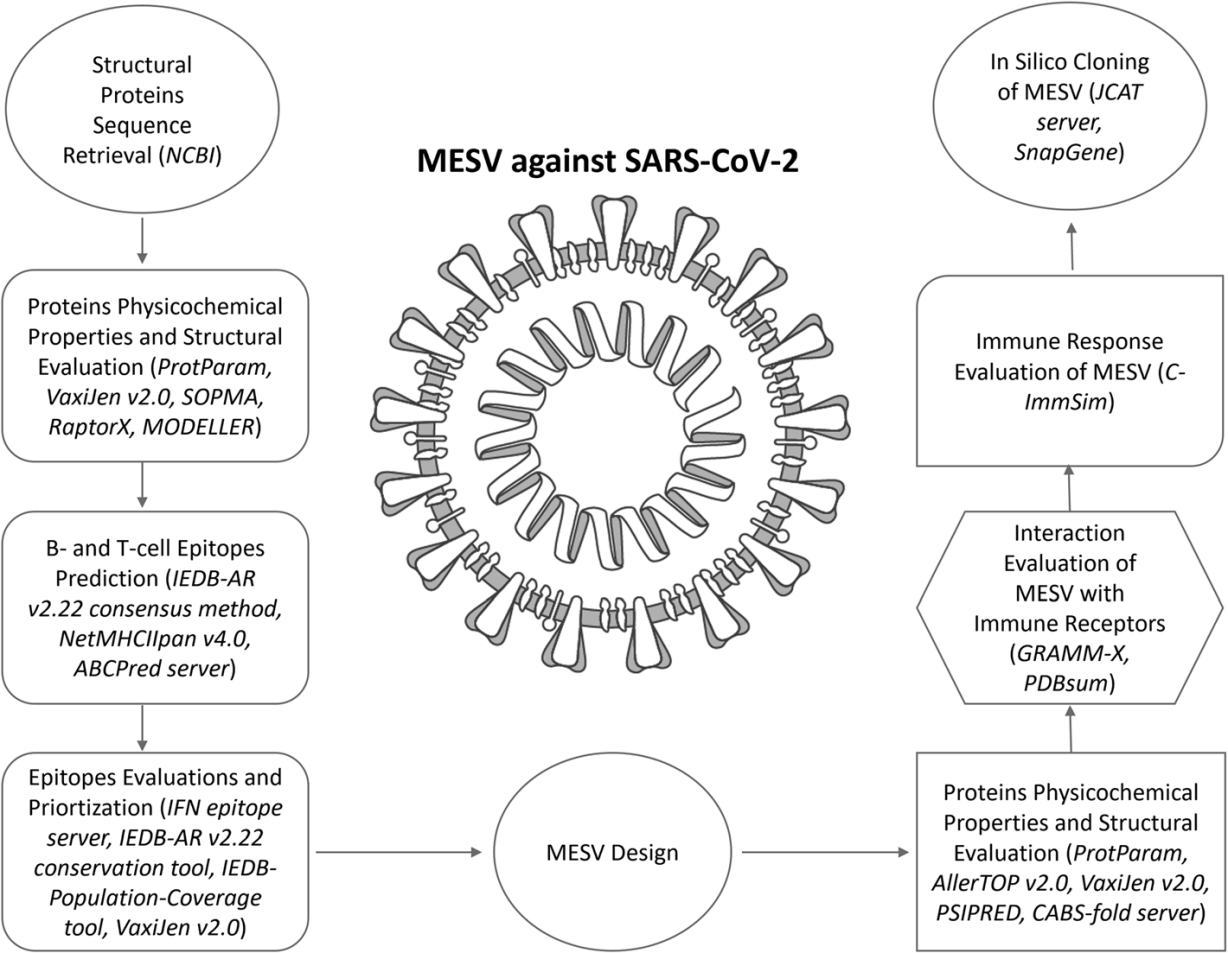
The schematic workflow used to develop MESV construct against SARS-CoV-2 structural proteins

Immunoinformatics approaches can be applied to examine viral antigens, prediction of its epitopes and assessment of its immunogenicity [28, 29]. Moreover, this approach could be both time and cost-effective [3, 30, 31]. Excessive respiratory infection can also resolve with T-cell reactions and antibodies [32]. Furthermore, rapid identification, isolation, disease prevention, and control measures are required to hinder its spread of SARS-CoV-2 at homes, communities and healthcare units [33, 34]. In various studies, therapeutic approaches against the Ebola virus, Zika virus and Middle East respiratory syndrome corona virus (MERS-CoV) were developed using immunoinformatics approaches [3, 31, 35]. The purpose of this study was to pinpoint the potential T-cell and B-cell epitopes from SARS-CoV-2 structural proteins which can be further joined through adjuvant and linkers to design a multiepitope-based subunit vaccine (MESV). Many in silico approaches were used to validate the structural and physiochemical properties of the MESV. To examine the binding interaction and stability of MESV with human pathogenic receptors, molecular docking analysis has also been carried out. In addition, in silico immune simulation was also performed to validate the immunogenic potential of designed MESV. At the end, the MESV codons were optimized for *Escherichia coli* system and in silico cloning was performed to ensure its expression profiling. Flow chart of methodology used in present study is graphically presented in Fig. 1.

## Methods

### Target proteins sequence and structural analyses

Main structural proteins, Surface glycoprotein (S [Genbank: QHD43416.1]), Envelope protein (E [Genbank: QHD43418.1]) and Membrane glycoprotein (M [QHD43419.1]) of SARS-CoV-2 were taken as targets for epitopes screening and vaccine designing against SARS-CoV-2. Their amino acid sequences were collected in fasta format from GenBank (https://www.ncbi.nlm.nih.gov/genbank/) [36]. Allergenicity and antigenicity (at a threshold of 0.4) of selected proteins were evaluated through AllerTOP v2.0 (https://www.ddg-pharmfac.net/AllerTOP/) and VaxiJen v2.0 (http://www.ddg-pharmfac.net/vaxijen/VaxiJen/VaxiJen.html) respectively [37, 38]. Three dimensional (3D) structure of S protein was retrieved from RCSB Protein Data Bank (PDB; https://www.rcsb.org/) [39]. However, 3D structures of other two proteins (E and M) were predicted using homology modeling approach, as their resolved structures are not available yet. RaptorX (http://raptorx.uchicago.edu/) and MODELLER v.9.12 (https://salilab.org/modeller/) were employed for homology modeling [40]. Predicted models were then visualized by Chimera (https://www.cgl.ucsf.edu/chimera/) [41]. Galaxy refines server (http://galaxy.seoklab.org/) and ModRefiner (https://zhanglab.ccmb.med.umich.edu/ModRefiner/) was used to refine the predicted models [42, 43]. Besides, the refined structure needs to be validated based on experimentally validated 3D structure of proteins. Refined structures were therefore applied in the PROSA web (https://prosa.services.came.sbg.ac.at/prosa.php) providing a quality score for a given structure [44]. The quality score beyond the usual range of native proteins indicates a possible error in protein structure. Ramachandran plot was created by rampage server (http://mordred.bioc.cam.ac.uk/~rapper/rampage.php), where the principle of PROCHECK is applied to validate the protein structure [45]. Structural analysis was performed to later investigate the positions of B-cell epitopes on target proteins.

### Prediction of B-cell and T-cell epitopes

The epitopes of B-cells help to detect viral infections in the immune system. ABCpred (http://crdd.osdd.net/raghava/abcpred/) was used to forecast 14-mer B cell epitopes for target proteins at 0.51 threshold [46]. Epitopes evident on the outer surface were picked, and other intracellular epitopes were removed. The Vaxijen server tested the antigenicity of the selected epitopes at a threshold of 0.5. B-cell epitope identification was based upon antigenicity, flexibility, linear epitope predictions, hydrophilicity, and surface accessibility [47]. Parker hydrophilicity prediction algorithms, Emini surface accessibility prediction method, Kolaskar and Tongaonkar antigenicity scale, and Karplus and Schulz flexibility prediction tool were used to perform hydrophilicity, accessibility of surface, antigenicity and flexibility analysis respectively [48]. As discontinuous epitopes become more evident and have higher dominant properties than linear epitopes, DiscoTop 2.0 server (http://www.cbs.dtu.dk/services/DiscoTope/) was used to forecast discontinuous epitopes from 3D structures of surface glycoprotein, membrane protein and envelope protein [49]. The position of epitopes on 3D structures of proteins was visualized by Pymol (https://pymol.org/2/) [50].

In vaccine designing, T-cell epitopes play a crucial role. More specifically, it reduces the cost and time compared with laboratory experiments [51]. IEDB consensus method (http://tools.iedb.org/mhcii/) was used to predict 8–11 mer MHC class-I and 11–14 mer MHC class-II epitopes. The results of this method are very important due to a large number of human leukocyte antigen (HLA) alleles used in the calculation. The sequence was given in a FASTA format and all the alleles were selected for prediction. Epitopes with less than 2 consensus score believed to be good binders and chosen for further research.

### Evaluation of predicted epitopes

Antigenicity and allergenicity of the selected epitopes were checked by Vaxijen v2.0 and Allergen FP v1.0 respectively [52]. Protein Digest server (http://db.systemsbiology.net:8080/proteomicsToolkit/proteinDigest.html) was used to predict epitopes digesting enzymes. ToxinPred (http://crdd.osdd.net/raghava/toxinpred/) was used for non-toxic/toxic properties prediction of epitopes. Non-toxic epitopes were selected for further analysis [53].

### Epitopes conservation and population coverage analysis

The degree of conservation of predicted T-cell and B-cell epitopes within the protein sequence was analyzed by IEDB conservancy analysis tool (http://tools.iedb.org/conservancy/). Epitopes having conservancy among all 3 selected proteins were shortlisted for further analyses [54].

The expression and distribution of HLA alleles vary depending on the world’s ethnicities and regions, thereby impacting the effective production of MESV [55]. The population coverage was calculated using the IEDB population coverage tool (http://tools.iedb.org/population/), and for this purpose MHC class-I and MHC class-II epitopes and corresponding HLA-binding alleles were considered. This tool estimates population coverage for each epitope for various regions of the world based on the distribution of HLA binding alleles [56].

### Multi-epitope-based subunit vaccine (MESV) designing and evaluation

Epitopes with the following characteristics are generally preferred to design a subunit vaccine: (a) highly antigenic, (b) immunogenic, (c) non-allergenic, (d) non-toxic, and (e) with significant population coverage. Therefore, only those epitopes were selected further to construct MESV following the above parameters. An adjuvant was attached with the EAAAK linker to the first cytotoxic T lymphocytes (CTL) epitope to improve the immune response. Other epitopes were linked using AAY, GPGPG, and KK linkers after validation of their interaction compatibility to preserve their independent immunogenic activity. β-defensin has been used as an adjuvant in the present research since it is a simple 45 amino acids long peptide that acts as an immunomodulator and as an antimicrobial agent both [57].

First, Blastp analysis was carried out using default parameters to confirm that the designed MESV sequence is non-homologous against the *Homo sapiens* proteome [58]. Protein with less than 37% is commonly known to be a non-homologous. Physiochemical properties of the designed MESV were accessed by the Protparam tool [59]. Protparam predicts various physiochemical properties like (half-life, theoretical isoelectric point [pI], instability index, grand average hydropathy, and aliphatic index) based on the amino acid approximations involved in the pk [60]. AllerTOP v.2.0 server was used to analyze the allergenicity of the MESV construct [38]. The secondary structure of the MESV construct was evaluated using a PSIPRED workbench [58]. This test also evaluated various vaccine properties such as alpha helices, extended chain, degree of beta turns, and random coil.

The 3D structure of MESV was predicted using the de novo modeling approach of CABS fold server (http://biocomp.chem.uw.edu.pl/CABSfold/), since the designed MESV was a series of epitopes and no appropriate template was available [61]. This server is based on a CABS modeling approach that combines a multi-scale modeling pipeline with an exchange replica Monte Carlo scheme. Predicted MESV 3D structure was modified using a galaxy refine server [62]. The Ramachandran plot analysis was carried out using the RAMPAGE server (http://mordred.bioc.cam.ac.uk/~rapper/rampage.php) [45], to confirm the quality of the refined MESV structure, followed by the structural validation analysis using the PROSA web server [44]. The ERRAT server (https://servicesn.mbi.ucla.edu/ERRAT/) was also used to evaluate the calculation of unbounded interactions in the MESV structure [63].

Besides, linear B-cell epitopes were predicted from the MESV using the ABCpred server [46]. Ellipro tool (http://tools.iedb.org/ellipro/) was used to predict the conformational B-cell epitopes of the designed MESV using default settings (maximum distance: 6 A^°^; minimum score: 0.5), provided by IEDB-AR v.2.22. It predicts epitopes by estimating residual protrusion index (PI), protein shape, and neighbor residue clustering [64].

### Molecular docking of MESV with human immune receptors

All together for the appropriate evocation of immune response, the interaction amongst the antigenic molecule and immune receptor molecule is essential. Molecular Docking was performed to analyze the interaction between MESV construct and human immune receptors. Toll-like receptors-3 (TLR3) has been thoroughly studied, and studies found its key role in antiviral immune response generation. GRAMM-X (http://vakser.compbio.ku.edu/resources/gramm/grammx/) was used for the MESV docking with TLR3 (PDB ID: 1ZIW) [65]. Pymol was utilized for visualization of the docked complexes [50]. Moreover, for the achievement of the conventional sketch of interactions among docked proteins, an online server PDBsum (http://www.ebi.ac.uk/thornton-srv/databases/cgi-bin/pdbsum/GetPage.pl?pdbcode=index.html) was utilized. It analyzes the protein-protein interactions among docked molecules [66].

### Immunogenicity evaluation of the vaccine construct

An in silico immune simulation was performed using C-ImmSim 10.1 server (http://150.146.2.1/C-IMMSIM/index.php?page=0) to validate the immunological responses of the designed MESV. C-ImmSim simulates the three main components of the functional mammal system (Thymus, lymph node, and bone marrow) [67]. The input parameters for the immune simulations are as follows: volume (10), HLA (A0101, A0101, B0702, B0702, DRB1_0101, DRB1_0101), random seed (12345), number of steps (100), number of injection set to 1. The rest of the parameters were considered to be the default.

### In silico cloning and codon optimization

Codon optimization is a method to improve the translation effectiveness of foreign genes in the host if the use of codon is different in both organisms. Codon optimization was carried out followed by in silico cloning, after the careful evaluation of MESV properties and immune response. To make this method consistent with the commonly used prokaryotic expression system; *E. coli* K12 [68], the java codon adaptation tool (http://www.jcat.de/) [69] was used for MESV codon optimization. The other available choices were selected to evade: (i) termination of rho-independent transcription, (ii) binding-site of prokaryote ribosome, and (iii) cleavage-sites of restriction enzymes. Codon adaptation index (CAI) [70] along with the GC (guanine and cytosine) contents were assessed. Sticky ends of the restriction sites of *Hin*dIII and *Bam*HI restriction enzymes were added to allow restriction and cloning, in the start/N terminal and end/C terminal of the modified MESV sequence, respectively. The modified nucleotide sequence of MESV was additionally cloned into the *E. coli* pET30a (+) vector by using SnapGene tool (https://www.snapgene.com/), to assure its in vitro expression.

## Results

### Sequence and structural analysis of the target proteins

All target structural proteins were found to be non-allergenic and highly antigenic. E protein was the most antigenic followed by M and S protein with 0.60, 0.51 and 0.46 antigenic values, respectively.

The 3D structure of S protein was retrieved from Protein-Data-Bank using ID: 6VYB [39]. The 3D structure of E protein was determined using homology modeling. Chain-A of envelope small membrane protein of SARS-CoV (PDB ID: 5X29) was found to be the best template (percent identity 88.71%) for E protein of SARS-CoV-2. However, no suitable template was found for M protein, so its structure was predicted by Raptor X [71]. Visualization of the models was done by Chimera (Additional file 1: Fig. S1). The quality factor (z-score) and Ramachandran plot values of refined predicted models are mentioned in Additional file 2: Table S1 (Additional file 1: Fig. S2–S3).

### B-cell epitopes prediction from target proteins

Total 23 linear epitopes (S-19, E-1, and M-3) were selected. Among the chosen linear epitopes, ‘**ILPVSMTKTSVDCT’** of S protein showed the highest antigenicity (1.6) and predicted score (Additional file 3: Table S2). The positions of epitopes on their respective protein structures were visualized by Pymol (Additional file 1: Fig. S4).

Identification of B cell epitope was based on antigenicity, flexibility, linear epitope predictions, hydrophilicity, and surface accessibility. Parker hydrophilicity prediction algorithms, Emini surface accessibility prediction method, Kolaskar and Tongaonkar antigenicity scale, and Karplus and Schulz flexibility prediction tool were used to perform hydrophilicity, accessibility of surface, antigenicity and flexibility analysis respectively (Additional file 1: Fig. S5–S7).

To further improve the specificity and variety of B-cell epitopes, Discotop 2.0 server was used to calculate surface abundance concerning residual contact number and use the novel amino acid score to forecast discontinuous epitopes. 3D structures of the target proteins were used to predict discontinuous epitopes; 90% specificity, − 3.700 thresholds and 22.000 Angstroms propensity score radius. Fifty-five discontinuous epitopes of S protein, 1 epitope of the E protein and 22 epitopes of M protein were calculated (Additional file 5: Table S4).

### T-cell epitopes prediction from target proteins

Epitopes that are bound to multiple alleles, highly antigenic, non-allergenic and 100% conserved were screened out, and their antigenicity and allergenicity were checked. Based on these criteria, 9 MHC class-I (S-3, E-3, and M-3) and 7 MHC class-II (S-1, E-3 and M-3) were shortlisted (Additional file 6: Table S5). Protein Digest server was used to estimate epitopes/peptides digesting enzymes. Epitopes digestible with many enzymes are not stable. Less enzyme digested epitopes, on the other hand, are very stable and favored vaccine candidates (Additional file 7: Table S6).

### Evaluation and selection of epitopes for further analyses

Total three CTL epitopes (S-1 and M-2), six HTL epitopes (E-3 and M-3), and four B-cell epitopes (S-3 and M-1) were selected to construct MESV (Table 1).

**Table 1:** Final selected epitopes from SARS-CoV-2 structural proteins used to design the multi-epitope-based subunit vaccine (MESV) construct

Sr.No	Epitope	Protein	Position	HLA alleles	Antigenicity	Immunogenicity
**MHC class-I**						
1	VRFPNITNLCPF	S	327–338	HLA-B*35:01	1.2	0.11
2	YRINWITGGIAI	M	71–82	HLA-B*27:05	1.2	0.61
3	SFRLFARTRSMW	M	99–110	HLA-B*57:01	0.6	0.05
**MHC class-II**						
1	LLFLAFVVFLLVTLA	E	18–32	HLA-DRB1*04:04	0.8	0.41
2	AFVVFLLVTLAILTA	E	22–36	HLA-DRB1*04:01	0.6	0.39
3	FVVFLLVTLAILTAL	E	23–37	HLA-DRB1*04:01	0.5	0.38
4	VTLACFVLAAVYRIN	M	60–74	HLA-DRB1*04:08	1.0	0.36
5	ASFRLFARTRSMWSF	M	98–112	HLA-DRB1*04:01	0.7	0.13
6	FRLFARTRSMWSFNP	M	100–114	HLA-DRB1*04:01	0.8	0.11
**B-Cell**						
1	SPTKLNDLCFTNVY	S	383	–	1.6	0.67
2	EILDITPCSFGGVS	S	583	–	1.6	0.81
3	ILPVSMTKTSVDCT	S	726	–	1.6	0.89
4	LEQWNLVIGFLFLT	M	17	–	0.9	0.67

**Fig. 2:**
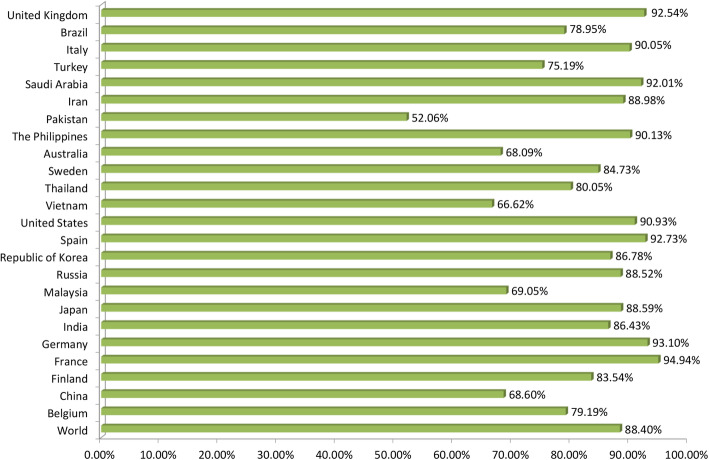
Population coverage of MESV epitopes around the globe predicted by IEDB population coverage tool

The selected epitopes showed 88.40% of the world population coverage (Fig. 2). Results revealed that predicted epitopes are showing promising population coverage of the countries strongly affected by COVID-19 including, Germany, France, Spain, Saudi Arabia, England, Italy, Iran, the Philippines, the United States, and Sweden.

### Designing of MESV

**Fig. 3:**
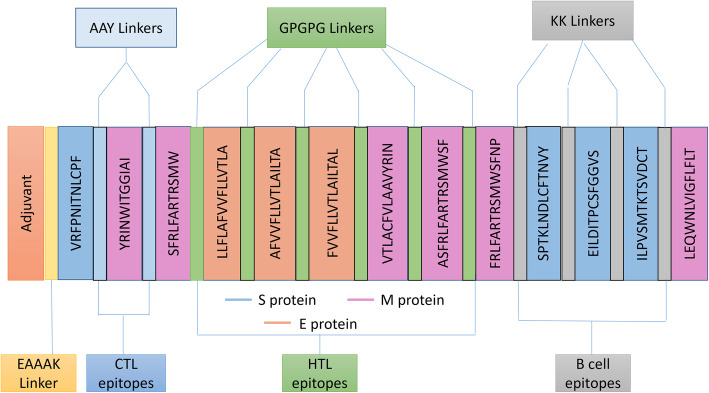
Schematic diagram of MESV construct: It has 276 amino acids, consisting of an adjuvant (orange) linked at N-terminal of MEV with the aid of EAAAK linker (yellow). AAY linkers (blue) used to join the CTL epitopes, GPGPG linkers (green) used to join the HTL epitopes and KK linkers (gray) used to join the B-cell epitopes

A MESV construct was further developed using all selected epitopes. Using the EAAAK linker, an adjuvant (45 amino acid long B-defensin) was bound at the beginning (to the MEV N-terminal). EAAAK linker reduces connections to other protein areas with efficient detachment and improves stability [58, 72]. Epitopes were merged in a sequential manner with AAY, GPGPG, and KK linkers, respectively, based on the compatibility of their interaction. Two hundred seventy-six amino acids represented the final MESV construct (Fig. 3).

### Sequence and structural analyses of MESV

First, Blastp analysis was carried out against the *Homo sapiens* proteome, and the results revealed that MESV does not resemble any human protein (higher or equal to 37%). The vaccine structure was then tested for toxicity, allergenicity, and antigenicity. MESV was found to be non-allergenic, highly antigenic (0.6737), and non-toxic. The mean half-life of the construct was calculated as 30 h in vitro, > 20 h in yeast and > 10 h in vivo. Molecular weight and theoretical pI of the vaccine were 3157.01 kDa and 10.31 respectively. Grand average hydropathicity was calculated as 0.395. A positive score of the grand average of hydropathy suggests its hydrophobic nature. The secondary structure analysis show that 74 (26.81%) amino acids were involved in the formation of α-helix, 67 amino acids (24.27%) formed β-strands and 135 amino acids (48.91%) form coils.

**Fig. 4:**
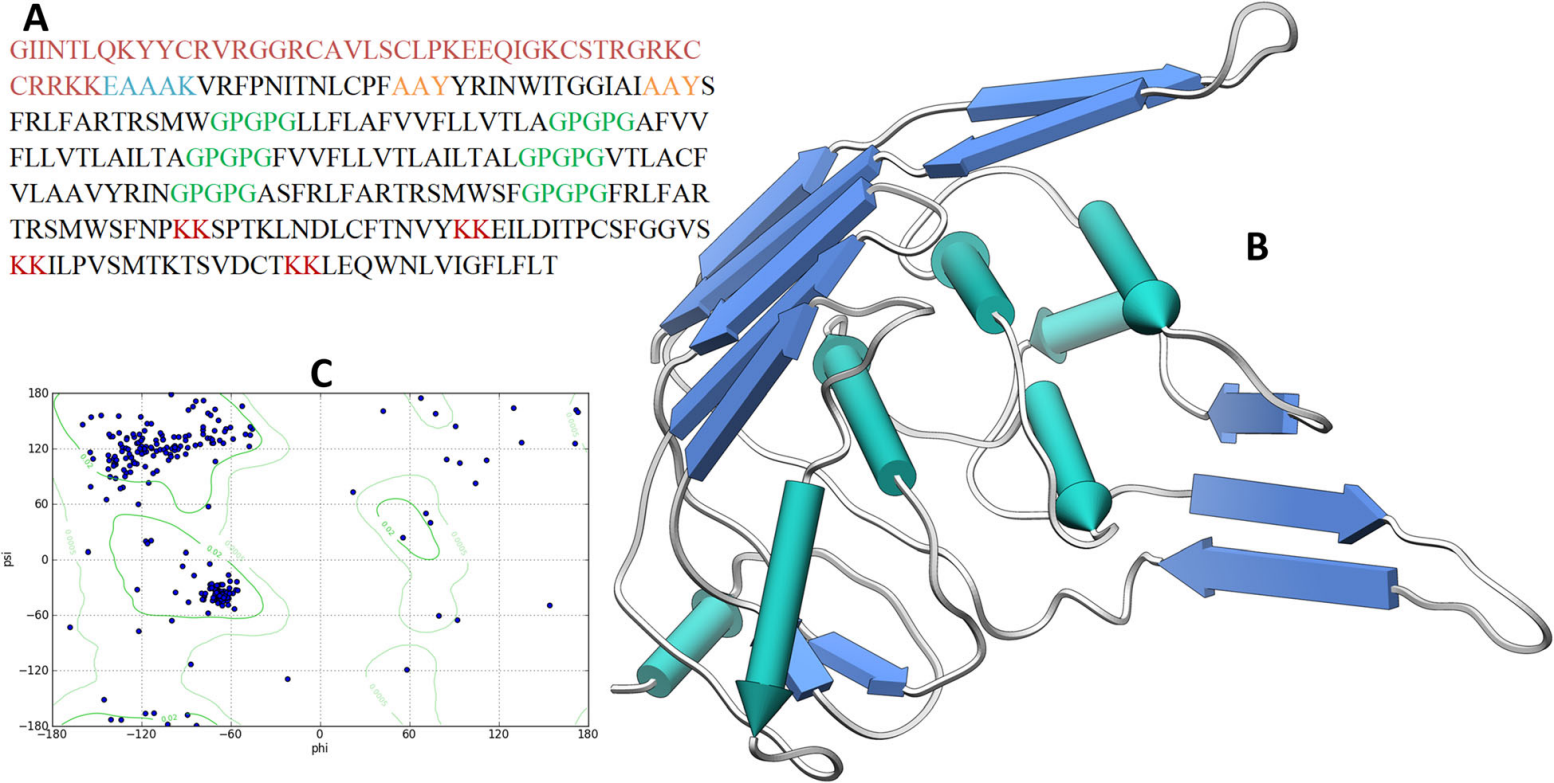
**a** MESV construct sequence. Epitopes sequence is in black. The adjuvant sequence is highlighted in brown color, EAAAK linker sequence is highlighted in blue, AAY linkers are highlighted with orange, GPGPG linkers are highlighted with green and KK linkers are highlighted with maroon color; **b** MESV construct refined 3D structure pipes representation (alpha helix: green; beta strands: blue; loops: gray); **c** Ramachandran plot analysis of predicted structure shows 89.4% residues are present in the favored region

CABS fold server was used to predict the tertiary structure of the MESV (Fig. 4). The structure was refined by the galaxy refine server. Ramachandran plot analysis of improved model showed that 89.4% amino acids are in favored region, 6.9% amino acids in the allowed region and 3.6% amino acids in the outlier region. Further analysis showed that the qRMSD is 0.544, MolProbity is 2.356, poor rotamers are 0.0%, clash score is 17.7 and z-score is − 4.8. In quality check analysis by ERRAT, the refined model score was 82.4561.

### B-cell epitopes screening from MESV

B-lymphocytes also produce antibodies that provide humoral immunity, in addition to the secretion of cytokines. Eighteen linear/continuous (Additional file 8: Table S7) and six conformational/ discontinuous epitopes (Additional file 9: Table S8) from the MESV sequence were predicted without altering ABCPred 2.0 and Ellipro prediction parameters.

### Molecular docking of MESV construct with TLR3

**Fig. 5:**
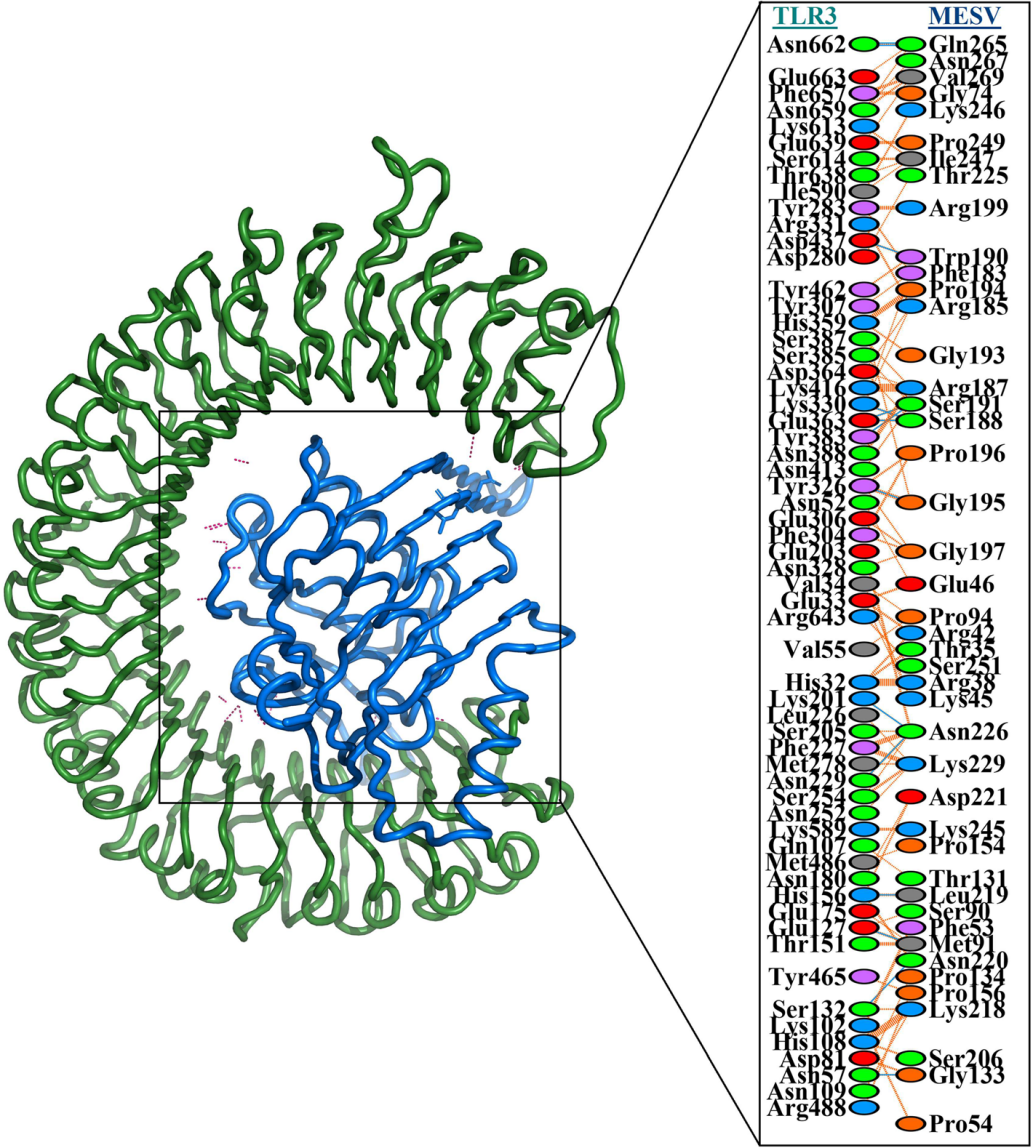
TLR3-MESV docked complex shown at the left in cartoon representation. Interacting residues of MESV are highlighted at right side. MESV vaccine construct displayed with blue color and TLR3 displayed with green color. Salt bridges are displayed with red color lines; other contacts are shown with orange color lines, and hydrogen bonds are displayed with blue color lines. The colors of interacting residues are interpreting the characteristics of amino acids (neutral: green, Cys: yellow, aromatic: pink, aliphatic: grey, positive: blue, negative: red, and Pro&Gly: orange)

To start the immune response, an appropriate interaction among the antigenic molecule and immune receptor molecule is needed. To decode the binding potential of MESV to the innate immune receptors, bioinformatic modeling driven molecular docking of the designed MESV to a representative innate immune receptor TLR3 was performed. The docking evaluation forecast that the best complex with a net global energy of − 22.36 kJ/mol. Visual analysis of the complex leads to the observation of the MESV’s deep binding in the center of TLR3 and favors rigorously hydrogen and weak van dar Waals interactions with specific TLR3 residues. PDBsum was used to gain insights and pin down possible residues of MESV making stable bonds with TLR3 (Fig. 5). Within 3 Å, the MESV was observed to form 14 hydrogen bonds with TLR3 potential residues.

### Immunogenicity evaluation of MESV

**Fig. 6:**
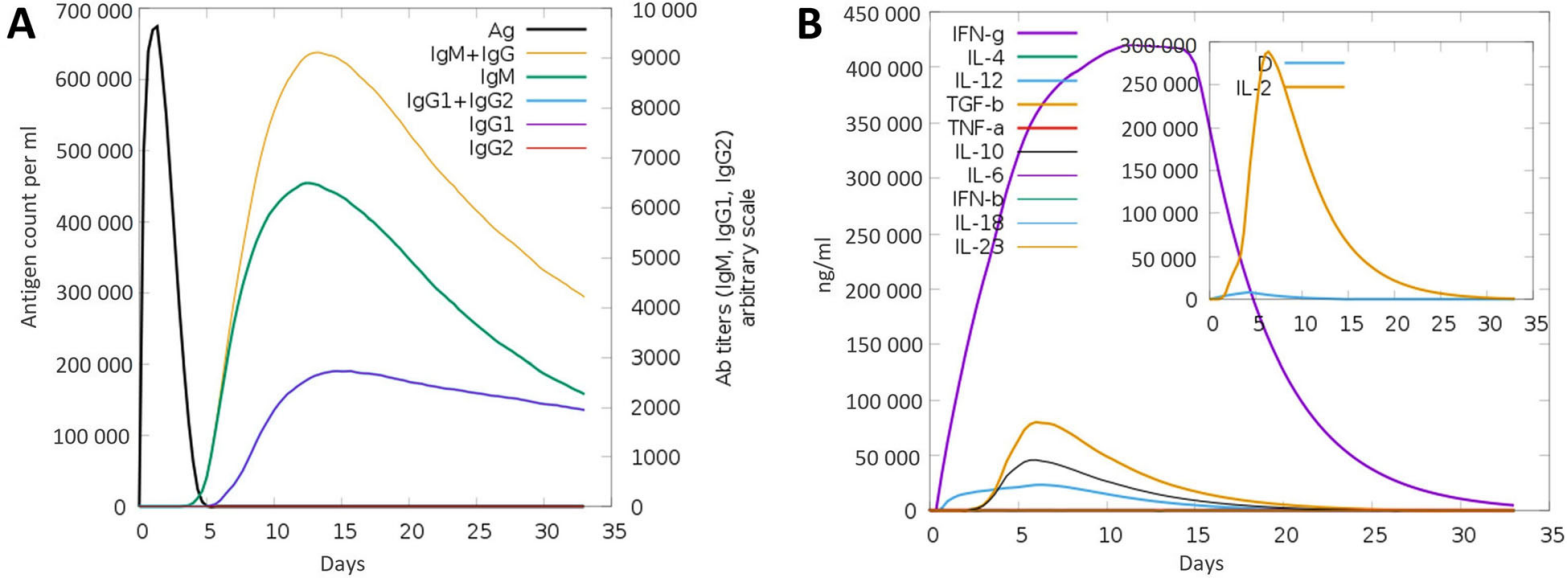
In silico immune response using MESV as antigen. **a** The antibodies, and **b** cytokines and interleukins

All secondary and primary immune responses tend to contribute significantly to the pathogen and may be consistent with the actual immune response. The in silico host immune system response to the antigen is shown in Fig. 6. The primary response was characterized by high IgG + IgG and IgM concentration, followed by IgM, IgG1 + IgG2 and IgG1 at both the secondary and primary stages with concomitant antigen reduction. Additionally, robust interleukin and cytokine response was observed. All of this indicates the MESV’s successful immune response and clearance after subsequent encounters.

### In silico cloning within *E. coli* system

**Fig. 7:**
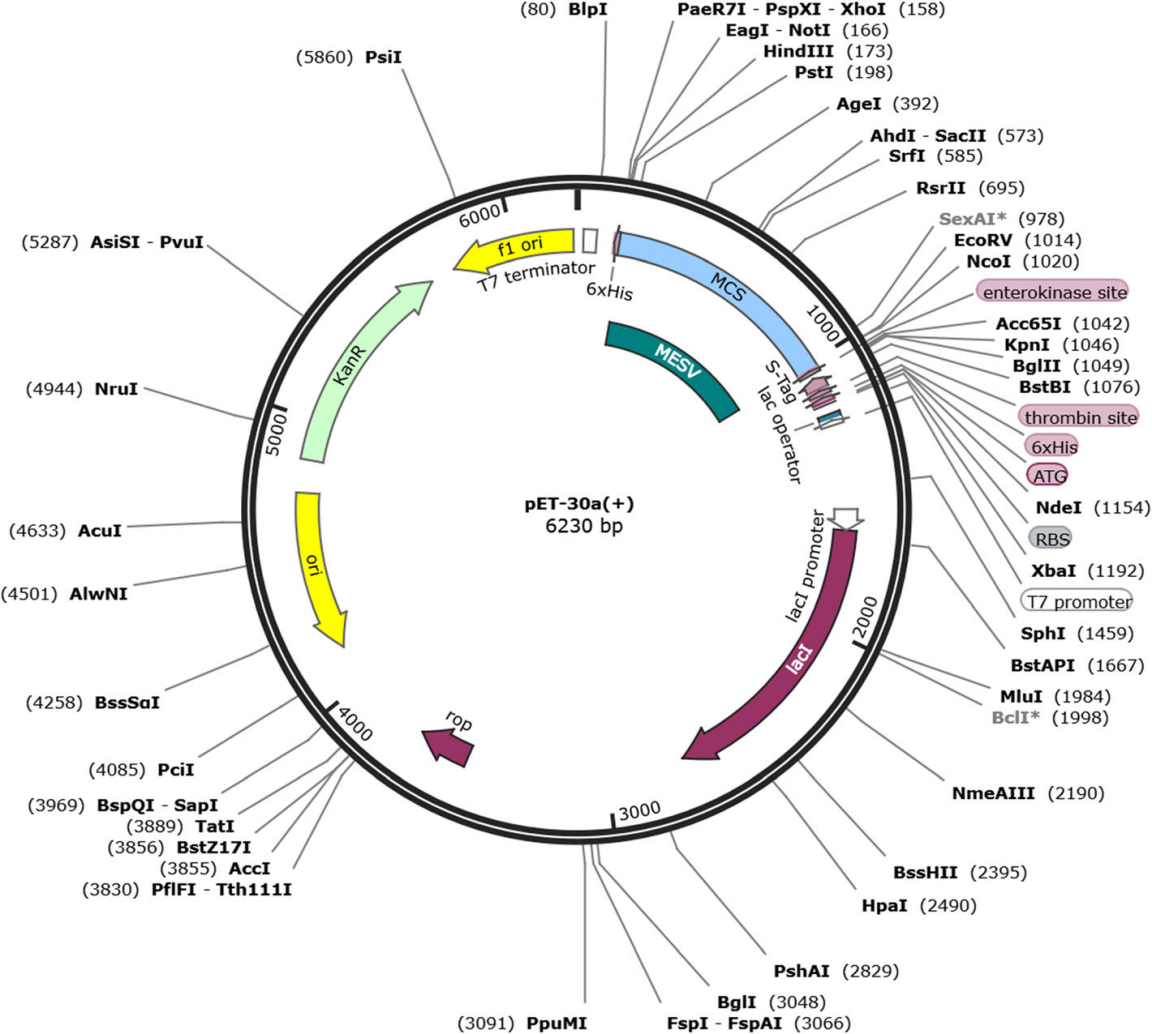
In silico cloning of codon optimized MESV into *E. coli* K12 expression system. The plasmid back-bone is kept in black color while the inserted DNA sequence is shown in green color

In silico cloning was done to assure the expression of MESV derived from SARS-CoV-2 in widely used *E. coli* hosts. First, the codons of MESV were modified according to the use of codons of *E. coli* expression system (strain K12). The optimized MESV construct contains 828 nucleotides, CAI value of 1.0 (0.8–1.0), and an optimal range of GC content of 53.2% (30–70%) demonstrating the strong potential for reliability and positive protein expression. In the following step, both ends of MESV optimized nucleotide sequence were attached to buffer compatible restriction enzymes *Bam*HI and *Hind*III restriction sites to assist the purification/cloning process. Finally, the refined MESV sequence was cloned to the several cloning sites of the pET30a (+) vector between the restriction sites. The clone was 6.23 kb long (Fig. 7).

## Discussion

CoVs have long been considered as insignificant pathogens causing “colds” in humans. In the twenty-first century, two extremely pathogenic CoVs named SARS-CoV and MERS-CoV emerged from the livestock reservoirs and cause deadly outbreaks. A new strain of CoV officially named as SARS-COV-2 was identified recently, which started a deadly global pandemic of COVID-19. The final dimension and impact of this pandemic are currently uncertain due to the rapidly changing situation [4]. After the recombination of various virus genomes particles, the novel virus infects the host cells rapidly. No reliable medication is currently available for the said infection. COVID-19 infection is a severe problem of morbidity and mortality worldwide. Unfortunately, the unavailability of the vaccinations against COVID-19 has impacted several precious lives, in different regions of the world. The emergence of COVID-19 results in a significant global disease burden, for which preventative measures are urgently needed. To successfully eradicate the disease, researchers have been trying to collect data associated with CoVs to understand its transmission, pathophysiology, and biology [73]. The rapid development of structural and genomic databases combined with computational tools helps in the design and discovery of new vaccine candidates.

Recent advancements in the immunological bioinformatics area have resulted in a variety of tools and servers that can lessen the time and cost of traditional vaccine advancement. Due to the problems in the selection of suitable antigen candidates and immunodominant epitopes, the development of effective multiepitope vaccines remains toilsome. Thus, the prediction of appropriate antigenic epitopes of a targeted protein by the immunoinformatics approaches is very essential for designing a MESV [74].

Here, we explored the development of epitope-based vaccines targeting the structural proteins (S, M, and E) of the SARS-CoV-2. These proteins play a crucial in the replication cycle and the virus particle structure. The S-protein plays an important part in binding the virus to the host cell surface receptors and consecutive fusion to promote the viral entrance in the host cell [75–77]. M and E proteins are important for replication, particle assembly within human cells, and viral entry [78, 79]. T- and B-cell epitopes of the target proteins were predicted to support the host’s immune response. The research was performed at primary, secondary and tertiary structural levels of proteins. IEDB analysis resource and ABCPred predicted B-cell conserved epitopes. The position of epitopes on 3D structures of proteins was visualized by Pymol. DiscoTop server was used to predict discontinuous epitopes. To further improve specificity and selectivity, allergenicity, toxicity, and physiochemical properties of predicted epitopes were checked. Digestion analysis verified that the peptides predicted during the analysis were stable and safe to use.

An appropriate MESV should be designed with B-cell, HTL, and CTL epitopes and cause effective reactions to a specific virus [80]. Few groups developed SARS-CoV-2 subunit vaccines but only used a single protein for the vaccine design [15, 81, 82] and the use of CTL epitopes only without taking into account the importance of HTL or B cell epitopes [83]. However, we have incorporated B-cell epitopes in addition to T-cell epitopes from multiple structural proteins, because of the functions they play in inducing antibody production and mediating its effective features [84]. Besides, the humoral response of memory B-cells can be easily overcome by the onset of antigens, while the cell-mediated immunity (T-cell immunity) in many cases leads to long-life immunity [85]. CTL limits pathogen spread through the secretion of unique antiviral cytokines and the identification and destruction of infected cells [86]. Therefore, the present vaccine construct has an advantage over already reported constructs.

The HLA alleles retain their response to T-cell epitopes which are highly polymorphic in different ethnic groups. To gain more population coverage, the T-cell epitopes are paired with more alleles. So we chose the HTL and CTL epitopes with their respective HLA alleles to predict the worldwide distribution of the alleles. The results showed that the chosen epitopes and their corresponding alleles preferably cover various geographical areas of the world. The selected epitopes cover 88.40% of the world population. France has the highest population, with 94.94%. In Germany, Spain, Saudi Arab, England, and Iran, the epidemic of SARS-CoV-2 happened in most significant measures. Vaccine candidates are therefore vital to protect individuals from SARS-CoV-2 infection in these geographical regions. The population coverage was 68.60% in China, where the virus first emerged and had several outbreaks.

Vaccine candidates were chosen form CTL, HTL, and B cell epitopes depending on their antigenicity, toxicity, immunogenicity, population coverage, and allergenicity. The MESV was designed by joining the HTL, CTL, and B cell epitopes with GPGPG, AAY, and KK linkers respectively. Linkers are introduced as an indispensable element in the MESV development to enhance folding, stabilization, and expression. Multi-epitope based vaccines are poorly immunogenic when used alone, and need adjuvant coupling [87]. Adjuvants are ingredients in a vaccine formulation that protects against infection and affect certain immune responses, growth, stability, and durability of antigens [88]. Therefore, 45 amino acids long, an adjuvant β-defensin, was integrated with the EAAAK linker whose length is 5, at N-terminal. The EAAAK linker is used to integrate the first epitope and adjuvant to facilitate efficient separation of the bifunctional fusion protein domains [89]. The final vaccine stretch with the addition of adjuvant and linkers was discovered to be 276 amino acid long.

The analysis of physiochemical characteristics of the MESV construct has shown that it is stable, basic, and hydrophobic. MESV was basic, according to the theoretical pI value, which can ensure stable physiological pH interaction. The calculated aliphatic index and instability index scores showed that the vaccine protein may be stable and thermostable. A positive score of the grand average of hydropathy suggests its hydrophobic nature. MESV has been found to be immunogenic, strongly antigenic, and non-allergenic. This suggests the ability of the epitopic vaccine to elicit a strong immune response without allergic reactions.

The 3D structure prediction provides extensive knowledge of the spatial arrangement of essential protein components and provides excellent support for the study of ligand interactions, protein functions, dynamics, and other proteins [90, 91]. After refinement, the desirable characteristics of the MESV construct improved considerably. The Ramachandran plot analysis shows that most residues are present in favored and allowed regions with very few residues in the disallowed region, which shows a satisfactory overall quality of the model. The good quality of designed MESV construct is further indicated by RMSD value, Poor Rotamers, Clash Score, and MolProbity. Various structure validation methods have been used to detect errors in the modeled MESV construct. The ERRAT quality factor (82.4%) and z-score (− 4.8) proved that the overall structure of the refined MESV is of good quality.

An adequate interaction between the antigen molecule and the immune receptor molecules is important for triggering an immune response. The refined MESV construct was then docked against TLR3 to examine adequate binding to immediate immune response. Stable interactions were observed among the MESV and TLR3 in molecular docking analysis, and less energy was needed for proficient binding.

B- and T-cell epitopes consisting multi-epitope vaccine should hypothetically activate both humoral and cellular immune reactions. With substantial IL-10 and IL-2 activities, our vaccine demonstrated the highest production of IFN-γ. Antibodies also provide extracellular SARS-CoV-2 protection. We have also noticed excess immunoglobulins that are active, i.e., IgM, IgG, and their isotypes that may be involved in switching isotype. Besides, the irrelevant Simpson index (D) recommends a diverse immune reaction that is conceivable as a subunit vaccine contains various B-cell and T-cell epitopes.

The translation efficiency of foreign genes inside the host system varies because of the incompatibility of mRNA codons, which require codon optimization for higher expression [92]. CAI value obtained was 1.0 and GC content (53.2%) was also within the optimum limit suggesting possible higher expression in the *E. coli* K-12 system. The main aim of MESV in silico cloning was to direct genetic engineers and molecular biologists on the expected expression level and the potential cloning sites in a particular expression system i.e., *E. coli* K12 system.

We applied the next-generation vaccine designing approach in this research to create a MESV construct, capable of generating immunological responses against the SARS-CoV-2. We believe that our vaccine will successfully produce humoral and cell-mediated immune responses. Interaction and binding patterns between receptor and vaccine protein were stable and higher. Moreover, in immune simulation, effective immune responses were observed in real life. Thus, MESV designed carefully using such a methodology could become an important asset in combating viral infections.

Computational/immunoinformatics approaches rely on experimental methodologies to generate initial raw data for further analyses. The data quality and efficiency of computational algorithms being applied, can limit the accuracy of immunoinformatics predictions. Therefore, further in vivo and in vitro investigations are however required to ensure the real potential of designed MESV to combat COVID-19.

## Conclusions

Taken together, we characterized SARS-CoV-2 structural proteins (S, E, and M) for antigenic epitopes and proposed a potential MESV utilizing various immunoinformatics and computational approaches. The findings of this research could save time and related costs for the study of experimental epitope targets. The MESV can activate all host immune system components and has adequate physicochemical and structural properties. It also appears to interact very stably with an innate immune receptor TLR3, making it more likely to be introduced into the host immune system. To reveal its effectiveness in the fight against COVID-19, however, additional in vitro and in vivo experiments are warranted.

## Supplementary information

**Additional file 1: Figure S1.** 3D structural representation of SARS-CoV-2 structural proteins: (A) S protein, (B) E protein and (C) M protein. **Figure S2.** (a) the E protein contains α-helix (77.33%, 58) and random coil (22.66%, 17); (b) the z-score (0.41) of the E protein; (c) the Ramachandran plot of refined structure shows 97.3, 2.7 and 0.0% residues in favored, allowed and disallowed region, respectively. **Figure S3.** (a) the M protein contains α-helix (40.54%, 90), β-strand (24.32%, 54) and random coil (35.13%, 78); (b) the z-score (− 3.88) of the M protein; (c) the Ramachandran plot of refined structure shows 96.8, 2.7 and 0.5% residues in favored, allowed and disallowed region, respectively. **Figure S4.** Specific sites of B cells predicted linear epitopes on the 3D structure of SARS-CoV-2 proteins: (A) S protein, (B) E protein and (C) M protein. **Figure S5.** (A) Prediction of antigenic determinants of S proteinusing Kolaskar and Tongaonkar antigenicity scale; (B) Beta Turns analyses in S protein using Chou and Fasman Beta Turn prediction; (C) Hydrophilicity Prediction of S protein using Parker Hydrophilicity; (D) Surface Accessibility Analyses of S protein using Emini Surface Accessibility Scale; (E) Flexibility Analyses of S protein using Karplus and Schulz Flexibility Scale. **Figure S6.** (A) Prediction of antigenic determinants of E protein using Kolaskar and Tongaonkar antigenicity scale; (B) Beta Turns Analyses in E protein using Chou and Fasman Beta Turn Prediction; (C) Hydrophilicity Prediction of E protein using Parker Hydrophilicity; (D) surface accessibility analyses of E protein using Emini Surface Accessibility Scale; (E) Flexibility Analyses of E protein using Karplus and Schulz Flexibility Scale. **Figure S7.** (A) Prediction of antigenic determinants of M protein using Kolaskar and Tongaonkar Antigenicity Scale; (B) Beta turns analyses in M protein using Chou and Fasman Beta Turn Prediction; (C) Hydrophilicity Prediction of M protein using Parker Hydrophilicity; (D) Surface Accessibility Analyses of M protein using Emini Surface Accessibility Scale; (E) Flexibility Analyses of M protein using Karplus and Schulz Flexibility Scale.

**Additional file 2: Table S1.** Structural details of the SARS-CoV-2 structural protein predicted models.

**Additional file 3: Table S2.** Linear B cell epitopes predicted through ABCPred 2.0 server (NT: nontoxic).

**Additional file 4: Table S3.** Emini surface accessibility of SARS-CoV-2 structural proteins.

**Additional file 5: Table S4.** Discontinuous epitopes predicted through DiscoTop 2.0 server.

**Additional file 6: Table S5.** MHC class-I allele and MHC class-II binding peptides with their antigenicity scores.

**Additional file 7: Table S6.** Digestion, allergenicity, toxicity and physiochemical profiling of selected peptides (NA: not allergic; NT: nontoxic).

**Additional file 8: Table S7.** Linear B cell epitopes predicted in vaccine construct.

**Additional file 9: Table S8.** Conformational epitopes in 3D structure of vaccine.

## Data Availability

Not applicable.

## Abbreviations

3D: Three dimensional
CAI: Codon adaptation index
CoV: Coronavirus
COVID-19: Coronavirus disease 2019
CTL: Cytotoxic T lymphocytes
E: Envelope
HLA: Human leukocyte antigen
HTL: Helper T lymphocyte
M: Membrane
MERS-CoV: Middle East respiratory syndrome corona virus
MESV: Multiepitope-based subunit vaccine
MHC: Major histocompatibility complex
PDB: Protein Data Bank
pI: Isoelectric point
S: Spike
SARS-CoV-2: Severe acute respiratory syndrome coronavirus 2
TLR3: Toll-like receptors-3
