# Supplementary material for: Reverse vaccinology assisted designing of multiepitope-based subunit vaccine against SARS-CoV-2
Source: Infect Dis Poverty. 2020 Sep 16;9:132. doi: 10.1186/s40249-020-00752-w (PMC7492789; doi:10.1186/s40249-020-00752-w)
Supplement: Supplementary file 1 — Additional file 1: Figure S1. 3D structural representation of SARS-CoV-2 structural proteins: (A) S protein, (B) E protein and (C) M protein. Figure S2. (a) the E protein contains α-helix (77.33%, 58) and random coil (22.66%, 17); (b) the z-score (0.41) of the E protein; (c) the Ramachandran plot of refined structure shows 97.3, 2.7 and 0.0% residues in favored, allowed and disallowed region, respectively. Figure S3. (a) the M protein contains α-helix (40.54%, 90), β-strand (24.32%, 54) and random coil (35.13%, 78); (b) the z-score (− 3.88) of the M protein; (c) the Ramachandran plot of refined structure shows 96.8, 2.7 and 0.5% residues in favored, allowed and disallowed region, respectively. Figure S4. Specific sites of B cells predicted linear epitopes on the 3D structure of SARS-CoV-2 proteins: (A) S protein, (B) E protein and (C) M protein. Figure S5. (A) Prediction of antigenic determinants of S proteinusing Kolaskar and Tongaonkar antigenicity scale; (B) Beta Turns analyses in S protein using Chou and Fasman Beta Turn prediction; (C) Hydrophilicity Prediction of S protein using Parker Hydrophilicity; (D) Surface Accessibility Analyses of S protein using Emini Surface Accessibility Scale; (E) Flexibility Analyses of S protein using Karplus and Schulz Flexibility Scale. Figure S6. (A) Prediction of antigenic determinants of E protein using Kolaskar and Tongaonkar antigenicity scale; (B) Beta Turns Analyses in E protein using Chou and Fasman Beta Turn Prediction; (C) Hydrophilicity Prediction of E protein using Parker Hydrophilicity; (D) surface accessibility analyses of E protein using Emini Surface Accessibility Scale; (E) Flexibility Analyses of E protein using Karplus and Schulz Flexibility Scale. Figure S7. (A) Prediction of antigenic determinants of M protein using Kolaskar and Tongaonkar Antigenicity Scale; (B) Beta turns analyses in M protein using Chou and Fasman Beta Turn Prediction; (C) Hydrophilicity Prediction of M protein using Parker Hydrophilicit [file 40249_2020_752_MOESM1_ESM.docx]

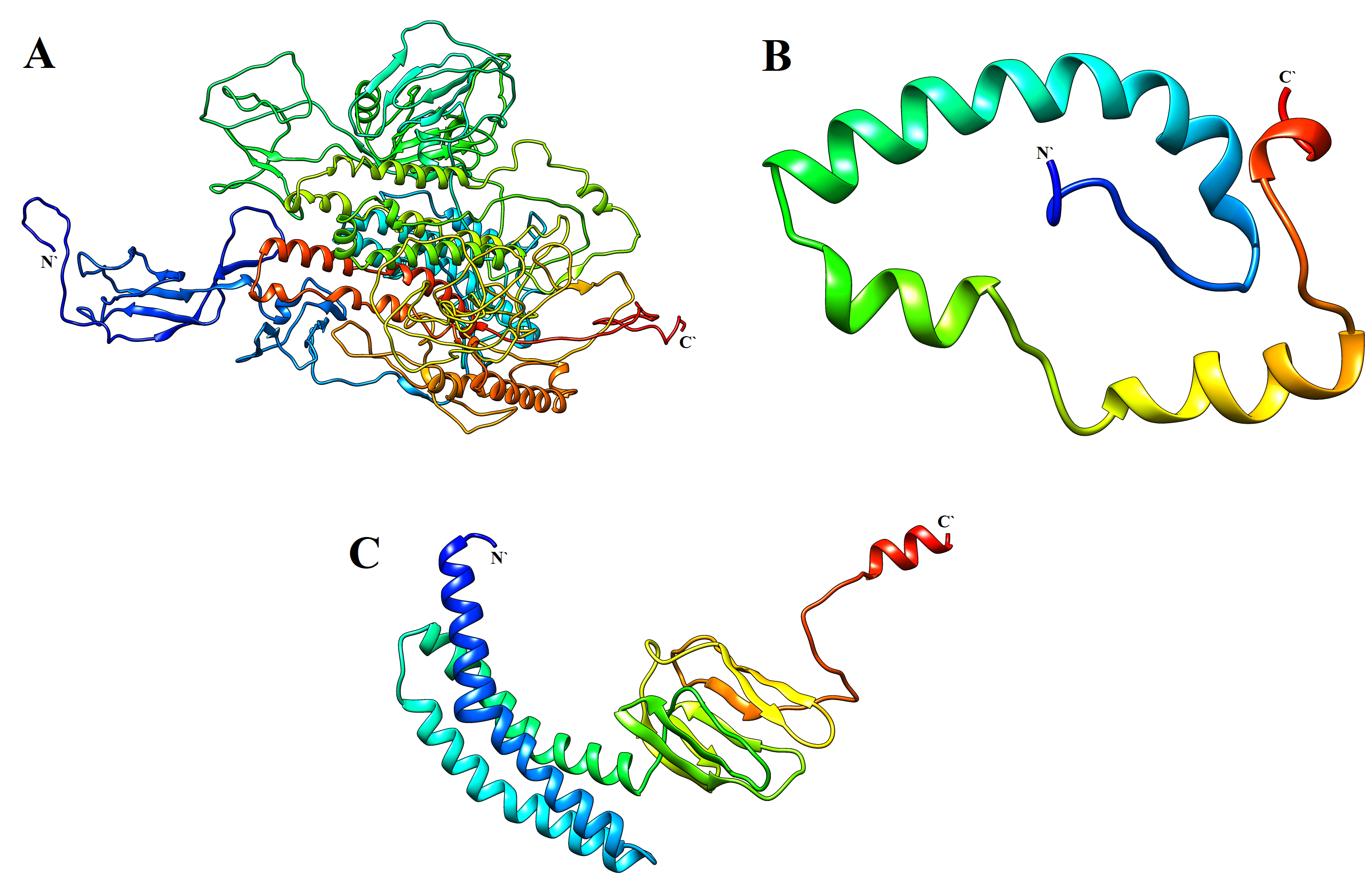


**Fig. S1.** 3D structural representation of SARS-CoV-2 structural proteins: (A) S protein, (B) E protein and (C) M protein.


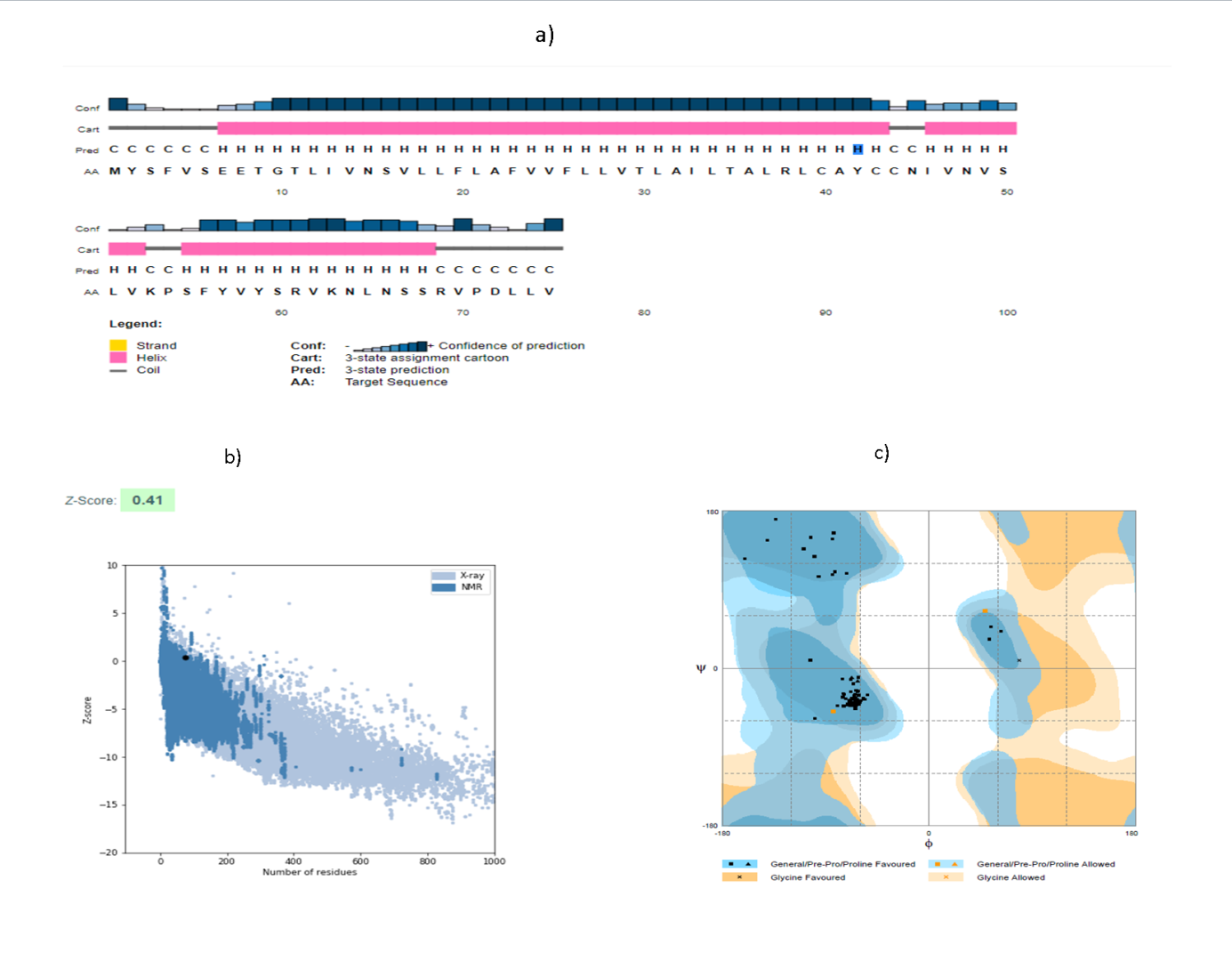


**Fig. S2:** (**a**) the E protein contains α-helix (77.33%, 58) and random coil (22.66%, 17); (**b**) the z-score (0.41) of the E protein; (**c**) the Ramachandran plot of refined structure shows 97.3%, 2.7% and 0.0% residues in favored, allowed and disallowed region, respectively.


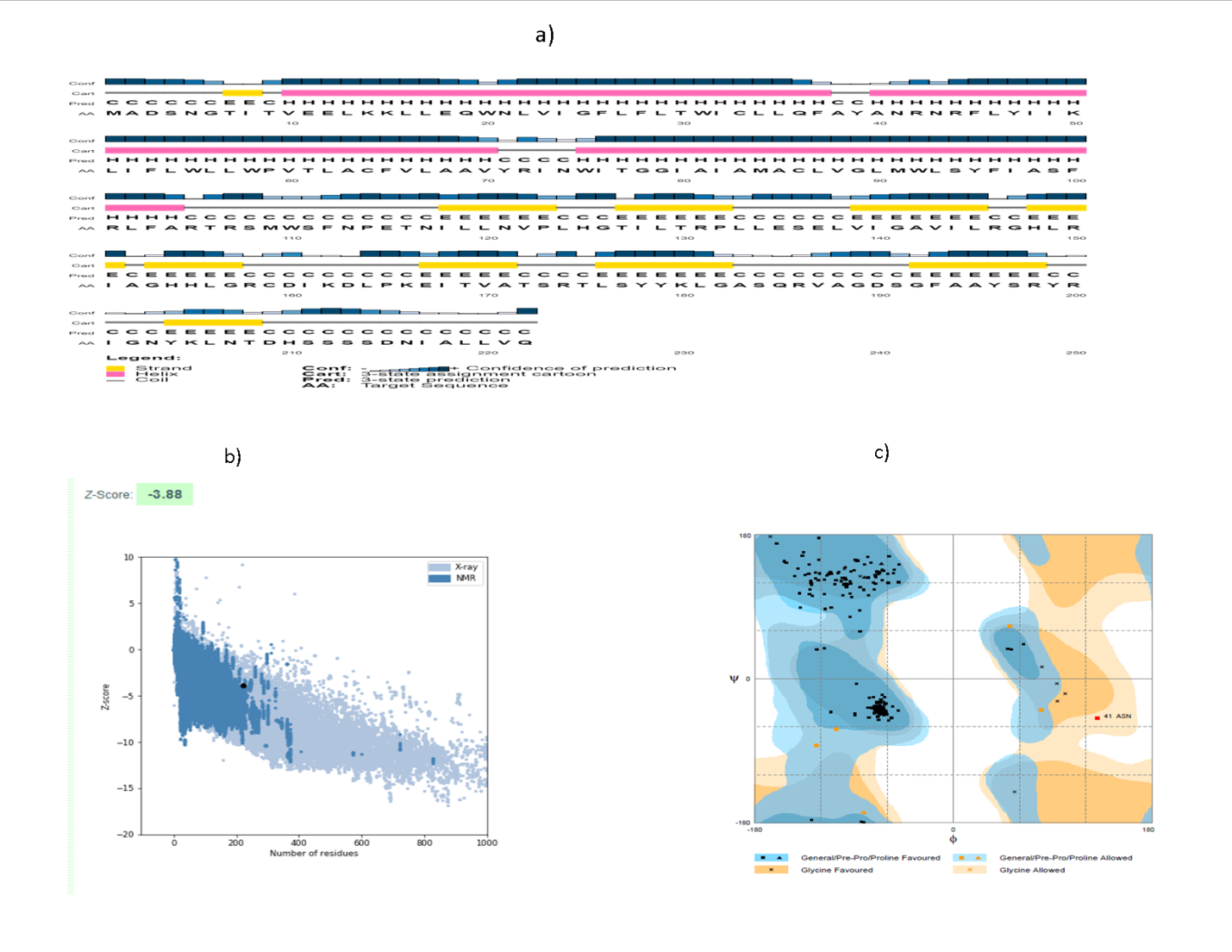


**Fig. S3:** (**a**) the M protein contains α-helix (40.54%, 90), β-strand (24.32%, 54) and random coil (35.13%, 78); (**b**) the z-score (-3.88) of the M protein; (**c**) the Ramachandran plot of refined structure shows 96.8%, 2.7% and 0.5% residues in favored, allowed and disallowed region, respectively.


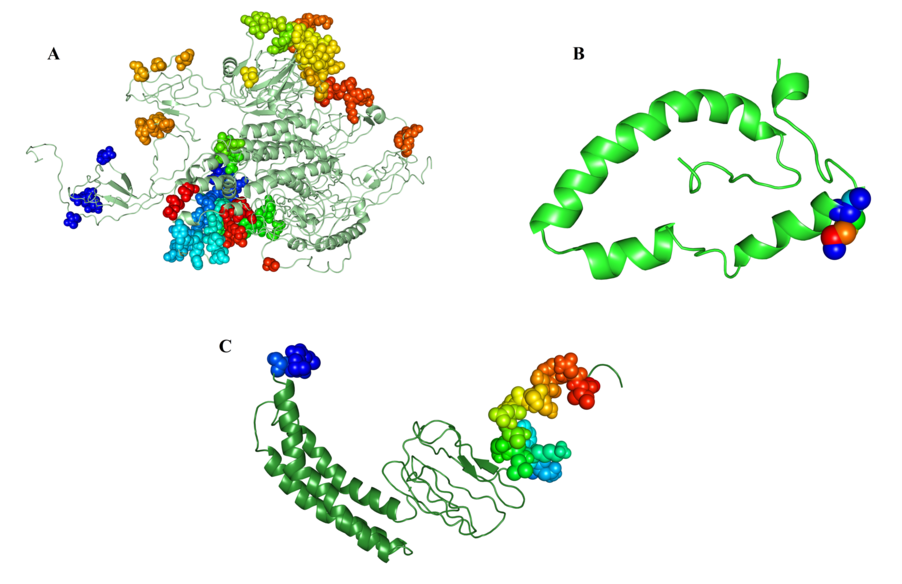


**Fig. S4.** Specific sites of B cells predicted linear epitopes on the 3D structure of SARS-CoV-2 proteins: (A) S protein, (B) E protein and (C) M protein.


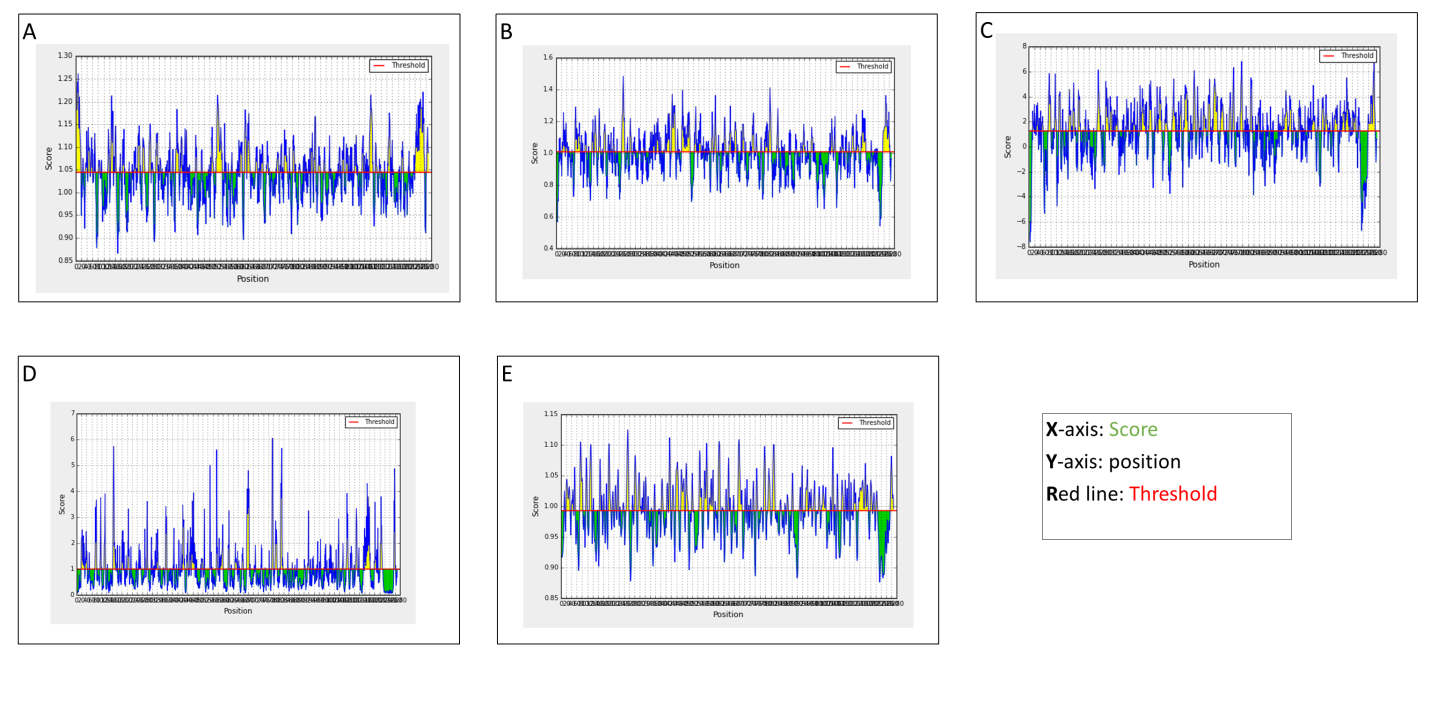


**Fig. S5:** (**A**) Prediction of antigenic determinants of S proteinusing Kolaskar and Tongaonkar antigenicity scale; (**B**) Beta Turns analyses in S protein using Chou and Fasman Beta Turn prediction; (**C**) Hydrophilicity Prediction of S protein using Parker Hydrophilicity; (**D**) Surface Accessibility Analyses of S protein using Emini Surface Accessibility Scale; (**E**) Flexibility Analyses of S protein using Karplus and Schulz Flexibility Scale.


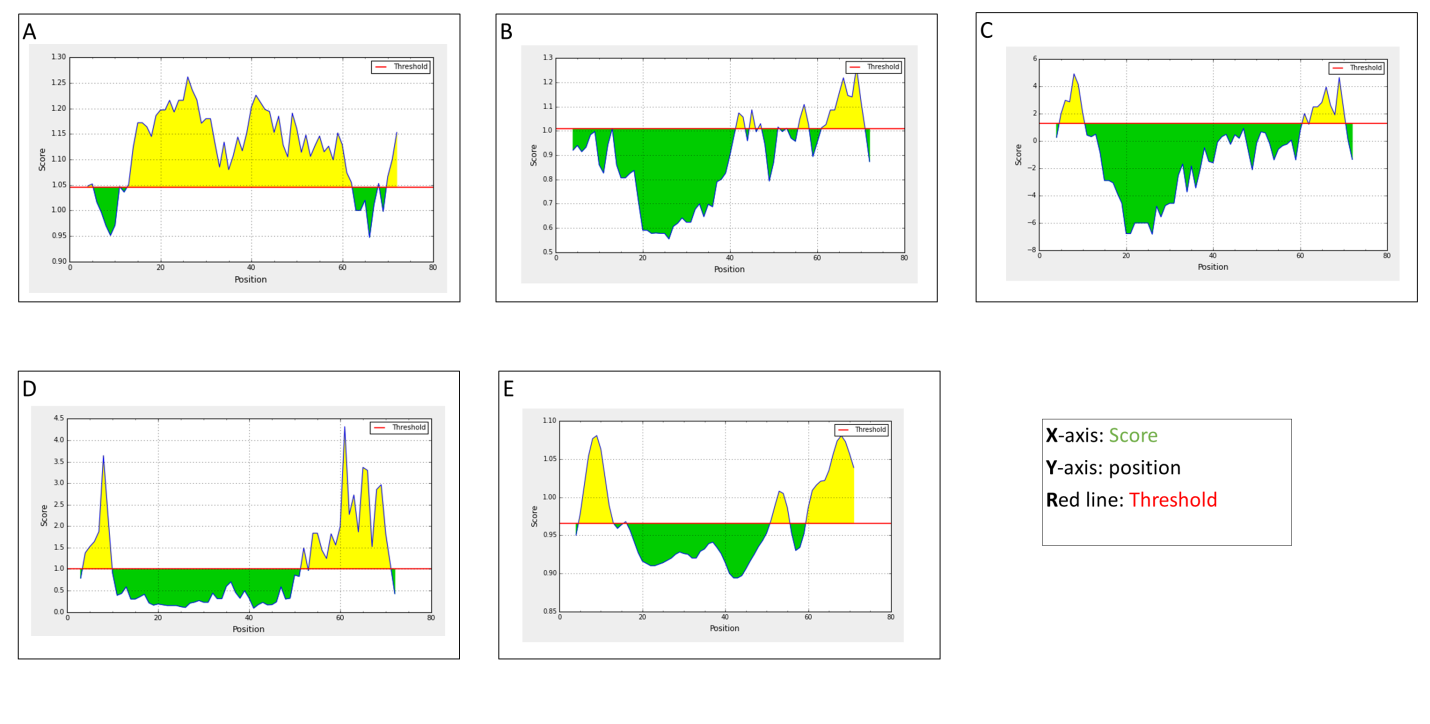


**Fig. S6:** (**A**) Prediction of antigenic determinants of E protein using Kolaskar and Tongaonkar antigenicity scale; (**B**) Beta Turns Analyses in E protein using Chou and Fasman Beta Turn Prediction; (**C**) Hydrophilicity Prediction of E protein using Parker Hydrophilicity; (**D**) surface accessibility analyses of E protein using Emini Surface Accessibility Scale; (**E**) Flexibility Analyses of E protein using Karplus and Schulz Flexibility Scale.


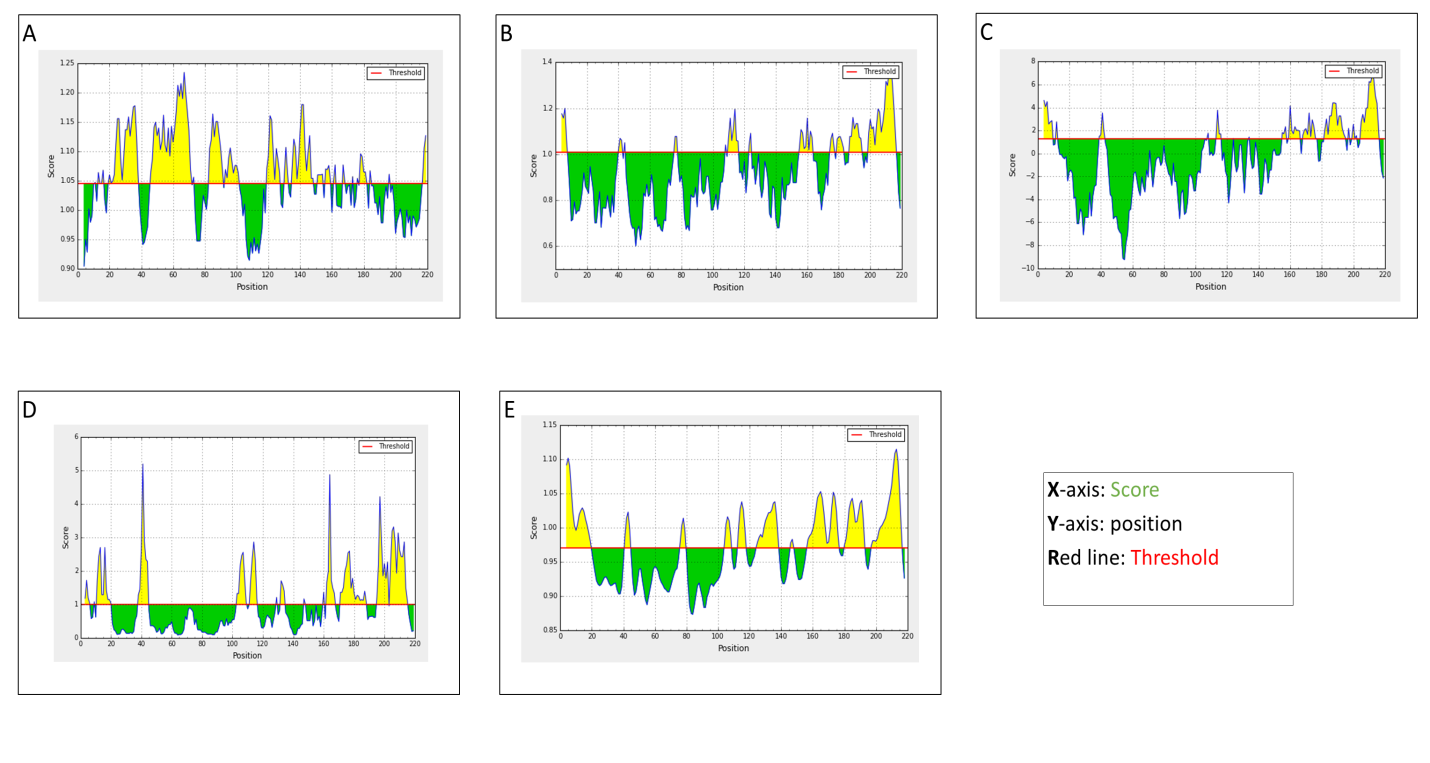


**Fig. S7:** (**A**) Prediction of antigenic determinants of M protein using Kolaskar and Tongaonkar Antigenicity Scale; (**B**) Beta turns analyses in M protein using Chou and Fasman Beta Turn Prediction; (**C**) Hydrophilicity Prediction of M protein using Parker Hydrophilicity; (**D**) Surface Accessibility Analyses of M protein using Emini Surface Accessibility Scale; (**E**) Flexibility Analyses of M protein using Karplus and Schulz Flexibility Scale.
