# Supplementary material for: Reverse vaccinology assisted designing of multiepitope-based subunit vaccine against SARS-CoV-2
Source: Infect Dis Poverty. 2020 Sep 16;9:132. doi: 10.1186/s40249-020-00752-w (PMC7492789; doi:10.1186/s40249-020-00752-w)
Supplement: Supplementary file 2 — Additional file 2: Table S1. Structural details of the SARS-CoV-2 structural protein predicted models. [file 40249_2020_752_MOESM2_ESM.docx]

Table S1. Structural details of the SARS-COV-2 structural protein predicted models.

| **Proteins** | **Tool utilized for modeling** | **Best template** | **Ramachandran plot** | | | **Model quality**  **(z-score)** |
| --- | --- | --- | --- | --- | --- | --- |
|  |  |  | **Favored region** | **Allowed region** | **Disallowed region** |  |
| **E** | Modeler | 5X29 | 97.3% | 2.7% | 0.0% | 0.41 |
| **M** | Raptor X | 5yckA | 96.8% | 2.7% | 0.5% | -3.88 |
