# Supplementary material for: Reverse vaccinology assisted designing of multiepitope-based subunit vaccine against SARS-CoV-2
Source: Infect Dis Poverty. 2020 Sep 16;9:132. doi: 10.1186/s40249-020-00752-w (PMC7492789; doi:10.1186/s40249-020-00752-w)
Supplement: Supplementary file 3 — Additional file 3: Table S2. Linear B cell epitopes predicted through ABCPred 2.0 server (NT: nontoxic). [file 40249_2020_752_MOESM3_ESM.docx]

Table S2. Linear B-cell epitopes predicted through ABCPred 2.0 server (NT: nontoxic)

| Protein | B-cell epitopes (Position) | Score | Antigenicity | Toxicity |
| --- | --- | --- | --- | --- |
| S | FSTFKCYGVSPTKL (374) | 0.90 | 0.8 | NT |
|  | ILPVSMTKTSVDCT (726) | 0.89 | 1.6 | NT |
|  | AGCLIGAEHVNNSY (647) | 0.87 | 0.8 | NT |
|  | LSSTASALGKLQDV (938) | 0.85 | 0.8 | NT |
|  | DLPIGINITRFQTL (228) | 0.84 | 1.1 | NT |
|  | LTGTGVLTESNKKF (546) | 0.83 | 0.7 | NT |
|  | SIIAYTMSLGAENS (691) | 0.81 | 0.7 | NT |
|  | EILDITPCSFGGVS (583) | 0.81 | 1.6 | NT |
|  | DPQTLEILDITPCS (578) | 0.81 | 1.2 | NT |
|  | VNFNFNGLTGTGVL (539) | 0.80 | 1.2 | NT |
|  | QPYRVVVLSFELLH (506) | 0.78 | 0.9 | NT |
|  | GVVFLHVTYVPAQE (1059) | 0.78 | 1.0 | NT |
|  | LPLVSSQCVNLTTR (8) | 0.76 | 1.3 | NT |
|  | ISVTTEILPVSMTK (720) | 0.75 | 1.2 | NT |
|  | AIPTNFTISVTTEI (713) | 0.73 | 0.8 | NT |
|  | LQYGSFCTQLNRAL (754) | 0.69 | 1.0 | NT |
|  | SPTKLNDLCFTNVY (383) | 0.67 | 1.6 | NT |
|  | TPCSFGGVSVITPG (588) | 0.56 | 0.9 | NT |
|  | MSLGAENSVAYSNN (697) | 0.51 | 0.8 | NT |
| E | FLLVTLAILTALRL (26) | 0.60 | 0.8 | NT |
| M | PVTLACFVLAAVYR (59) | 0.75 | 0.9 | NT |
|  | GGIAIAMACLVGLM (78) | 0.68 | 0.8 | NT |
|  | LEQWNLVIGFLFLT (17) | 0.67 | 0.9 | NT |
