# Supplementary material for: Reverse vaccinology assisted designing of multiepitope-based subunit vaccine against SARS-CoV-2
Source: Infect Dis Poverty. 2020 Sep 16;9:132. doi: 10.1186/s40249-020-00752-w (PMC7492789; doi:10.1186/s40249-020-00752-w)
Supplement: Supplementary file 4 — Additional file 4: Table S3. Emini surface accessibility of SARS-CoV-2 structural proteins. [file 40249_2020_752_MOESM4_ESM.docx]

Table S3. Emini surface accessibility of SARS-CoV-2 structural proteins

| Position | Residues | Start | End | Peptide | Score |
| --- | --- | --- | --- | --- | --- |
| S | | | | | |
| 3 | **V** | 1 | 6 | MFVFLV | 0.088 |
| 4 | **F** | 2 | 7 | FVFLVL | 0.073 |
| 5 | **L** | 3 | 8 | VFLVLL | 0.07 |
| 6 | **V** | 4 | 9 | FLVLLP | 0.145 |
| 7 | **L** | 5 | 10 | LVLLPL | 0.138 |
| 8 | **L** | 6 | 11 | VLLPLV | 0.124 |
| 9 | **P** | 7 | 12 | LLPLVS | 0.225 |
| 10 | **L** | 8 | 13 | LPLVSS | 0.365 |
| 11 | **V** | 9 | 14 | PLVSSQ | 0.766 |
| 12 | **S** | 10 | 15 | LVSSQC | 0.266 |
| 13 | **S** | 11 | 16 | VSSQCV | 0.239 |
| 14 | **Q** | 12 | 17 | SSQCVN | 0.518 |
| 15 | **C** | 13 | 18 | SQCVNL | 0.319 |
| 16 | **V** | 14 | 19 | QCVNLT | 0.343 |
| 17 | **N** | 15 | 20 | CVNLTT | 0.286 |
| 18 | **L** | 16 | 21 | VNLTTR | 1.045 |
| 19 | **T** | 17 | 22 | NLTTRT | 2.032 |
| 20 | **T** | 18 | 23 | LTTRTQ | 2.189 |
| 21 | **R** | 19 | 24 | TTRTQL | 2.189 |
| 22 | **T** | 20 | 25 | TRTQLP | 2.345 |
| 23 | **Q** | 21 | 26 | RTQLPP | 2.513 |
| 24 | **L** | 22 | 27 | TQLPPA | 1.296 |
| 25 | **P** | 23 | 28 | QLPPAY | 1.407 |
| 26 | **P** | 24 | 29 | LPPAYT | 1.173 |
| 27 | **A** | 25 | 30 | PPAYTN | 2.286 |
| 28 | **Y** | 26 | 31 | PAYTNS | 1.982 |
| 29 | **T** | 27 | 32 | AYTNSF | 1.11 |
| 30 | **N** | 28 | 33 | YTNSFT | 1.585 |
| 31 | **S** | 29 | 34 | TNSFTR | 1.982 |
| 32 | **F** | 30 | 35 | NSFTRG | 1.359 |
| 33 | **T** | 31 | 36 | SFTRGV | 0.627 |
| 34 | **R** | 32 | 37 | FTRGVY | 0.733 |
| 35 | **G** | 33 | 38 | TRGVYY | 1.327 |
| 36 | **V** | 34 | 39 | RGVYYP | 1.422 |
| 37 | **Y** | 35 | 40 | GVYYPD | 1.212 |
| 38 | **Y** | 36 | 41 | VYYPDK | 2.449 |
| 39 | **P** | 37 | 42 | YYPDKV | 2.449 |
| 40 | **D** | 38 | 43 | YPDKVF | 1.354 |
| 41 | **K** | 39 | 44 | PDKVFR | 1.692 |
| 42 | **V** | 40 | 45 | DKVFRS | 1.466 |
| 43 | **F** | 41 | 46 | KVFRSS | 1.177 |
| 44 | **R** | 42 | 47 | VFRSSV | 0.437 |
| 45 | **S** | 43 | 48 | FRSSVL | 0.485 |
| 46 | **S** | 44 | 49 | RSSVLH | 0.763 |
| 47 | **V** | 45 | 50 | SSVLHS | 0.522 |
| 48 | **L** | 46 | 51 | SVLHST | 0.562 |
| 49 | **H** | 47 | 52 | VLHSTQ | 0.726 |
| 50 | **S** | 48 | 53 | LHSTQD | 1.634 |
| 51 | **T** | 49 | 54 | HSTQDL | 1.634 |
| 52 | **Q** | 50 | 55 | STQDLF | 1.04 |
| 53 | **D** | 51 | 56 | TQDLFL | 0.64 |
| 54 | **L** | 52 | 57 | QDLFLP | 0.686 |
| 55 | **F** | 53 | 58 | DLFLPF | 0.343 |
| 56 | **L** | 54 | 59 | LFLPFF | 0.178 |
| 57 | **P** | 55 | 60 | FLPFFS | 0.289 |
| 58 | **F** | 56 | 61 | LPFFSN | 0.536 |
| 59 | **F** | 57 | 62 | PFFSNV | 0.483 |
| 60 | **S** | 58 | 63 | FFSNVT | 0.451 |
| 61 | **N** | 59 | 64 | FSNVTW | 0.547 |
| 62 | **V** | 60 | 65 | SNVTWF | 0.547 |
| 63 | **T** | 61 | 66 | NVTWFH | 0.555 |
| 64 | **W** | 62 | 67 | VTWFHA | 0.349 |
| 65 | **F** | 63 | 68 | TWFHAI | 0.33 |
| 66 | **H** | 64 | 69 | WFHAIH | 0.311 |
| 67 | **A** | 65 | 70 | FHAIHV | 0.219 |
| 68 | **I** | 66 | 71 | HAIHVS | 0.339 |
| 69 | **H** | 67 | 72 | AIHVSG | 0.247 |
| 70 | **V** | 68 | 73 | IHVSGT | 0.353 |
| 71 | **S** | 69 | 74 | HVSGTN | 0.809 |
| 72 | **G** | 70 | 75 | VSGTNG | 0.588 |
| 73 | **T** | 71 | 76 | SGTNGT | 1.144 |
| 74 | **N** | 72 | 77 | GTNGTK | 1.708 |
| 75 | **G** | 73 | 78 | TNGTKR | 3.379 |
| 76 | **T** | 74 | 79 | NGTKRF | 2.028 |
| 77 | **K** | 75 | 80 | GTKRFD | 2.106 |
| 78 | **R** | 76 | 81 | TKRFDN | 3.422 |
| 79 | **F** | 77 | 82 | KRFDNP | 3.666 |
| 80 | **D** | 78 | 83 | RFDNPV | 1.361 |
| 81 | **N** | 79 | 84 | FDNPVL | 0.573 |
| 82 | **P** | 80 | 85 | DNPVLP | 1.023 |
| 83 | **V** | 81 | 86 | NPVLPF | 0.53 |
| 84 | **L** | 82 | 87 | PVLPFN | 0.53 |
| 85 | **P** | 83 | 88 | VLPFND | 0.573 |
| 86 | **F** | 84 | 89 | LPFNDG | 0.764 |
| 87 | **N** | 85 | 90 | PFNDGV | 0.687 |
| 88 | **D** | 86 | 91 | FNDGVY | 0.697 |
| 89 | **G** | 87 | 92 | NDGVYF | 0.697 |
| 90 | **V** | 88 | 93 | DGVYFA | 0.438 |
| 91 | **Y** | 89 | 94 | GVYFAS | 0.351 |
| 92 | **F** | 90 | 95 | VYFAST | 0.512 |
| 93 | **A** | 91 | 96 | YFASTE | 1.195 |
| 94 | **S** | 92 | 97 | FASTEK | 1.525 |
| 95 | **T** | 93 | 98 | ASTEKS | 2.36 |
| 96 | **E** | 94 | 99 | STEKSN | 3.757 |
| 97 | **K** | 95 | 100 | TEKSNI | 1.965 |
| 98 | **S** | 96 | 101 | EKSNII | 0.955 |
| 99 | **N** | 97 | 102 | KSNIIR | 1.08 |
| 100 | **I** | 98 | 103 | SNIIRG | 0.534 |
| 101 | **I** | 99 | 104 | NIIRGW | 0.419 |
| 102 | **R** | 100 | 105 | IIRGWI | 0.183 |
| 103 | **G** | 101 | 106 | IRGWIF | 0.226 |
| 104 | **W** | 102 | 107 | RGWIFG | 0.319 |
| 105 | **I** | 103 | 108 | GWIFGT | 0.235 |
| 106 | **F** | 104 | 109 | WIFGTT | 0.342 |
| 107 | **G** | 105 | 110 | IFGTTL | 0.269 |
| 108 | **T** | 106 | 111 | FGTTLD | 0.64 |
| 109 | **T** | 107 | 112 | GTTLDS | 0.99 |
| 110 | **L** | 108 | 113 | TTLDSK | 2.001 |
| 111 | **D** | 109 | 114 | TLDSKT | 2.001 |
| 112 | **S** | 110 | 115 | LDSKTQ | 2.401 |
| 113 | **K** | 111 | 116 | DSKTQS | 3.902 |
| 114 | **T** | 112 | 117 | SKTQSL | 1.927 |
| 115 | **Q** | 113 | 118 | KTQSLL | 1.186 |
| 116 | **S** | 114 | 119 | TQSLLI | 0.416 |
| 117 | **L** | 115 | 120 | QSLLIV | 0.214 |
| 118 | **L** | 116 | 121 | SLLIVN | 0.198 |
| 119 | **I** | 117 | 122 | LLIVNN | 0.238 |
| 120 | **V** | 118 | 123 | LIVNNA | 0.292 |
| 121 | **N** | 119 | 124 | IVNNAT | 0.511 |
| 122 | **N** | 120 | 125 | VNNATN | 1.171 |
| 123 | **A** | 121 | 126 | NNATNV | 1.171 |
| 124 | **T** | 122 | 127 | NATNVV | 0.541 |
| 125 | **N** | 123 | 128 | ATNVVI | 0.236 |
| 126 | **V** | 124 | 129 | TNVVIK | 0.467 |
| 127 | **V** | 125 | 130 | NVVIKV | 0.24 |
| 128 | **I** | 126 | 131 | VVIKVC | 0.08 |
| 129 | **K** | 127 | 132 | VIKVCE | 0.187 |
| 130 | **V** | 128 | 133 | IKVCEF | 0.218 |
| 131 | **C** | 129 | 134 | KVCEFQ | 0.538 |
| 132 | **E** | 130 | 135 | VCEFQF | 0.233 |
| 133 | **F** | 131 | 136 | CEFQFC | 0.168 |
| 134 | **Q** | 132 | 137 | EFQFCN | 0.505 |
| 135 | **F** | 133 | 138 | FQFCND | 0.487 |
| 136 | **C** | 134 | 139 | QFCNDP | 0.869 |
| 137 | **N** | 135 | 140 | FCNDPF | 0.434 |
| 138 | **D** | 136 | 141 | CNDPFL | 0.414 |
| 139 | **P** | 137 | 142 | NDPFLG | 0.764 |
| 140 | **F** | 138 | 143 | DPFLGV | 0.353 |
| 141 | **L** | 139 | 144 | PFLGVY | 0.331 |
| 142 | **G** | 140 | 145 | FLGVYY | 0.335 |
| 143 | **V** | 141 | 146 | LGVYYH | 0.527 |
| 144 | **Y** | 142 | 147 | GVYYHK | 1.277 |
| 145 | **Y** | 143 | 148 | VYYHKN | 2.076 |
| 146 | **H** | 144 | 149 | YYHKNN | 4.497 |
| 147 | **K** | 145 | 150 | YHKNNK | 5.74 |
| 148 | **N** | 146 | 151 | HKNNKS | 4.909 |
| 149 | **N** | 147 | 152 | KNNKSW | 3.794 |
| 150 | **K** | 148 | 153 | NNKSWM | 1.877 |
| 151 | **S** | 149 | 154 | NKSWME | 2.022 |
| 152 | **W** | 150 | 155 | KSWMES | 1.685 |
| 153 | **M** | 151 | 156 | SWMESE | 1.459 |
| 154 | **E** | 152 | 157 | WMESEF | 0.943 |
| 155 | **S** | 153 | 158 | MESEFR | 1.756 |
| 156 | **E** | 154 | 159 | ESEFRV | 1.317 |
| 157 | **F** | 155 | 160 | SEFRVY | 1.192 |
| 158 | **R** | 156 | 161 | EFRVYS | 1.192 |
| 159 | **V** | 157 | 162 | FRVYSS | 0.922 |
| 160 | **Y** | 158 | 163 | RVYSSA | 1.076 |
| 161 | **S** | 159 | 164 | VYSSAN | 0.883 |
| 162 | **S** | 160 | 165 | YSSANN | 1.914 |
| 163 | **A** | 161 | 166 | SSANNC | 0.655 |
| 164 | **N** | 162 | 167 | SANNCT | 0.705 |
| 165 | **N** | 163 | 168 | ANNCTF | 0.456 |
| 166 | **C** | 164 | 169 | NNCTFE | 0.781 |
| 167 | **T** | 165 | 170 | NCTFEY | 0.761 |
| 168 | **F** | 166 | 171 | CTFEYV | 0.351 |
| 169 | **E** | 167 | 172 | TFEYVS | 0.878 |
| 170 | **Y** | 168 | 173 | FEYVSQ | 1.054 |
| 171 | **V** | 169 | 174 | EYVSQP | 1.881 |
| 172 | **S** | 170 | 175 | YVSQPF | 0.941 |
| 173 | **Q** | 171 | 176 | VSQPFL | 0.495 |
| 174 | **P** | 172 | 177 | SQPFLM | 0.66 |
| 175 | **F** | 173 | 178 | QPFLMD | 0.823 |
| 176 | **L** | 174 | 179 | PFLMDL | 0.392 |
| 177 | **M** | 175 | 180 | FLMDLE | 0.439 |
| 178 | **D** | 176 | 181 | LMDLEG | 0.501 |
| 179 | **L** | 177 | 182 | MDLEGK | 1.216 |
| 180 | **E** | 178 | 183 | DLEGKQ | 2.128 |
| 181 | **G** | 179 | 184 | LEGKQG | 1.261 |
| 182 | **K** | 180 | 185 | EGKQGN | 2.459 |
| 183 | **Q** | 181 | 186 | GKQGNF | 1.229 |
| 184 | **G** | 182 | 187 | KQGNFK | 2.484 |
| 185 | **N** | 183 | 188 | QGNFKN | 1.998 |
| 186 | **F** | 184 | 189 | GNFKNL | 0.951 |
| 187 | **K** | 185 | 190 | NFKNLR | 1.883 |
| 188 | **N** | 186 | 191 | FKNLRE | 2.028 |
| 189 | **L** | 187 | 192 | KNLREF | 2.028 |
| 190 | **R** | 188 | 193 | NLREFV | 0.753 |
| 191 | **E** | 189 | 194 | LREFVF | 0.405 |
| 192 | **F** | 190 | 195 | REFVFK | 0.983 |
| 193 | **V** | 191 | 196 | EFVFKN | 0.807 |
| 194 | **F** | 192 | 197 | FVFKNI | 0.327 |
| 195 | **K** | 193 | 198 | VFKNID | 0.63 |
| 196 | **N** | 194 | 199 | FKNIDG | 0.84 |
| 197 | **I** | 195 | 200 | KNIDGY | 1.52 |
| 198 | **D** | 196 | 201 | NIDGYF | 0.658 |
| 199 | **G** | 197 | 202 | IDGYFK | 0.818 |
| 200 | **Y** | 198 | 203 | DGYFKI | 0.818 |
| 201 | **F** | 199 | 204 | GYFKIY | 0.768 |
| 202 | **K** | 200 | 205 | YFKIYS | 1.04 |
| 203 | **I** | 201 | 206 | FKIYSK | 1.327 |
| 204 | **Y** | 202 | 207 | KIYSKH | 2.085 |
| 205 | **S** | 203 | 208 | IYSKHT | 1.505 |
| 206 | **K** | 204 | 209 | YSKHTP | 3.319 |
| 207 | **H** | 205 | 210 | SKHTPI | 1.485 |
| 208 | **T** | 206 | 211 | KHTPIN | 1.782 |
| 209 | **P** | 207 | 212 | HTPINL | 0.735 |
| 210 | **I** | 208 | 213 | TPINLV | 0.401 |
| 211 | **N** | 209 | 214 | PINLVR | 0.544 |
| 212 | **L** | 210 | 215 | INLVRD | 0.587 |
| 213 | **V** | 211 | 216 | NLVRDL | 0.691 |
| 214 | **R** | 212 | 217 | LVRDLP | 0.665 |
| 215 | **D** | 213 | 218 | VRDLPQ | 1.396 |
| 216 | **L** | 214 | 219 | RDLPQG | 1.861 |
| 217 | **P** | 215 | 220 | DLPQGF | 0.823 |
| 218 | **Q** | 216 | 221 | LPQGFS | 0.66 |
| 219 | **G** | 217 | 222 | PQGFSA | 0.809 |
| 220 | **F** | 218 | 223 | QGFSAL | 0.431 |
| 221 | **S** | 219 | 224 | GFSALE | 0.431 |
| 222 | **A** | 220 | 225 | FSALEP | 0.674 |
| 223 | **L** | 221 | 226 | SALEPL | 0.642 |
| 224 | **E** | 222 | 227 | ALEPLV | 0.355 |
| 225 | **P** | 223 | 228 | LEPLVD | 0.588 |
| 226 | **L** | 224 | 229 | EPLVDL | 0.588 |
| 227 | **V** | 225 | 230 | PLVDLP | 0.525 |
| 228 | **D** | 226 | 231 | LVDLPI | 0.238 |
| 229 | **L** | 227 | 232 | VDLPIG | 0.285 |
| 230 | **P** | 228 | 233 | DLPIGI | 0.27 |
| 231 | **I** | 229 | 234 | LPIGIN | 0.26 |
| 232 | **G** | 230 | 235 | PIGINI | 0.221 |
| 233 | **I** | 231 | 236 | IGINIT | 0.206 |
| 234 | **N** | 232 | 237 | GINITR | 0.575 |
| 235 | **I** | 233 | 238 | INITRF | 0.503 |
| 236 | **T** | 234 | 239 | NITRFQ | 1.244 |
| 237 | **R** | 235 | 240 | ITRFQT | 1.116 |
| 238 | **F** | 236 | 241 | TRFQTL | 1.313 |
| 239 | **Q** | 237 | 242 | RFQTLL | 0.75 |
| 240 | **T** | 238 | 243 | FQTLLA | 0.387 |
| 241 | **L** | 239 | 244 | QTLLAL | 0.369 |
| 242 | **L** | 240 | 245 | TLLALH | 0.29 |
| 243 | **A** | 241 | 246 | LLALHR | 0.393 |
| 244 | **L** | 242 | 247 | LALHRS | 0.639 |
| 245 | **H** | 243 | 248 | ALHRSY | 1.214 |
| 246 | **R** | 244 | 249 | LHRSYL | 0.991 |
| 247 | **S** | 245 | 250 | HRSYLT | 1.734 |
| 248 | **Y** | 246 | 251 | RSYLTP | 1.97 |
| 249 | **L** | 247 | 252 | SYLTPG | 0.995 |
| 250 | **T** | 248 | 253 | YLTPGD | 1.24 |
| 251 | **P** | 249 | 254 | LTPGDS | 1.061 |
| 252 | **G** | 250 | 255 | TPGDSS | 1.724 |
| 253 | **D** | 251 | 256 | PGDSSS | 1.601 |
| 254 | **S** | 252 | 257 | GDSSSG | 1.025 |
| 255 | **S** | 253 | 258 | DSSSGW | 1.089 |
| 256 | **S** | 254 | 259 | SSSGWT | 0.941 |
| 257 | **G** | 255 | 260 | SSGWTA | 0.709 |
| 258 | **W** | 256 | 261 | SGWTAG | 0.524 |
| 259 | **T** | 257 | 262 | GWTAGA | 0.395 |
| 260 | **A** | 258 | 263 | WTAGAA | 0.403 |
| 261 | **G** | 259 | 264 | TAGAAA | 0.387 |
| 262 | **A** | 260 | 265 | AGAAAY | 0.42 |
| 263 | **A** | 261 | 266 | GAAAYY | 0.652 |
| 264 | **A** | 262 | 267 | AAAYYV | 0.489 |
| 265 | **Y** | 263 | 268 | AAYYVG | 0.479 |
| 266 | **Y** | 264 | 269 | AYYVGY | 0.743 |
| 267 | **V** | 265 | 270 | YYVGYL | 0.607 |
| 268 | **G** | 266 | 271 | YVGYLQ | 0.67 |
| 269 | **Y** | 267 | 272 | VGYLQP | 0.662 |
| 270 | **L** | 268 | 273 | GYLQPR | 1.746 |
| 271 | **Q** | 269 | 274 | YLQPRT | 2.546 |
| 272 | **P** | 270 | 275 | LQPRTF | 1.407 |
| 273 | **R** | 271 | 276 | QPRTFL | 1.407 |
| 274 | **T** | 272 | 277 | PRTFLL | 0.67 |
| 275 | **F** | 273 | 278 | RTFLLK | 0.867 |
| 276 | **L** | 274 | 279 | TFLLKY | 0.693 |
| 277 | **L** | 275 | 280 | FLLKYN | 0.772 |
| 278 | **K** | 276 | 281 | LLKYNE | 1.545 |
| 279 | **Y** | 277 | 282 | LKYNEN | 3.013 |
| 280 | **N** | 278 | 283 | KYNENG | 3.615 |
| 281 | **E** | 279 | 284 | YNENGT | 2.609 |
| 282 | **N** | 280 | 285 | NENGTI | 1.167 |
| 283 | **G** | 281 | 286 | ENGTIT | 1.047 |
| 284 | **T** | 282 | 287 | NGTITD | 1.01 |
| 285 | **I** | 283 | 288 | GTITDA | 0.634 |
| 286 | **T** | 284 | 289 | TITDAV | 0.476 |
| 287 | **D** | 285 | 290 | ITDAVD | 0.551 |
| 288 | **A** | 286 | 291 | TDAVDC | 0.421 |
| 289 | **V** | 287 | 292 | DAVDCA | 0.295 |
| 290 | **D** | 288 | 293 | AVDCAL | 0.146 |
| 291 | **C** | 289 | 294 | VDCALD | 0.241 |
| 292 | **A** | 290 | 295 | DCALDP | 0.501 |
| 293 | **L** | 291 | 296 | CALDPL | 0.248 |
| 294 | **D** | 292 | 297 | ALDPLS | 0.619 |
| 295 | **P** | 293 | 298 | LDPLSE | 1.061 |
| 296 | **L** | 294 | 299 | DPLSET | 1.857 |
| 297 | **S** | 295 | 300 | PLSETK | 2.223 |
| 298 | **E** | 296 | 301 | LSETKC | 0.771 |
| 299 | **T** | 297 | 302 | SETKCT | 1.349 |
| 300 | **K** | 298 | 303 | ETKCTL | 0.83 |
| 301 | **C** | 299 | 304 | TKCTLK | 0.959 |
| 302 | **T** | 300 | 305 | KCTLKS | 0.89 |
| 303 | **L** | 301 | 306 | CTLKSF | 0.385 |
| 304 | **K** | 302 | 307 | TLKSFT | 1.038 |
| 305 | **S** | 303 | 308 | LKSFTV | 0.534 |
| 306 | **F** | 304 | 309 | KSFTVE | 1.121 |
| 307 | **T** | 305 | 310 | SFTVEK | 1.121 |
| 308 | **V** | 306 | 311 | FTVEKG | 0.827 |
| 309 | **E** | 307 | 312 | TVEKGI | 0.67 |
| 310 | **K** | 308 | 313 | VEKGIY | 0.727 |
| 311 | **G** | 309 | 314 | EKGIYQ | 1.697 |
| 312 | **I** | 310 | 315 | KGIYQT | 1.414 |
| 313 | **Y** | 311 | 316 | GIYQTS | 0.948 |
| 314 | **Q** | 312 | 317 | IYQTSN | 1.54 |
| 315 | **T** | 313 | 318 | YQTSNF | 1.902 |
| 316 | **S** | 314 | 319 | QTSNFR | 2.378 |
| 317 | **N** | 315 | 320 | TSNFRV | 1.019 |
| 318 | **F** | 316 | 321 | SNFRVQ | 1.223 |
| 319 | **R** | 317 | 322 | NFRVQP | 1.411 |
| 320 | **V** | 318 | 323 | FRVQPT | 1.266 |
| 321 | **Q** | 319 | 324 | RVQPTE | 2.533 |
| 322 | **P** | 320 | 325 | VQPTES | 1.733 |
| 323 | **T** | 321 | 326 | QPTESI | 1.637 |
| 324 | **E** | 322 | 327 | PTESIV | 0.701 |
| 325 | **S** | 323 | 328 | TESIVR | 0.888 |
| 326 | **I** | 324 | 329 | ESIVRF | 0.533 |
| 327 | **V** | 325 | 330 | SIVRFP | 0.476 |
| 328 | **R** | 326 | 331 | IVRFPN | 0.571 |
| 329 | **F** | 327 | 332 | VRFPNI | 0.571 |
| 330 | **P** | 328 | 333 | RFPNIT | 1.111 |
| 331 | **N** | 329 | 334 | FPNITN | 0.912 |
| 332 | **I** | 330 | 335 | PNITNL | 0.868 |
| 333 | **T** | 331 | 336 | NITNLC | 0.301 |
| 334 | **N** | 332 | 337 | ITNLCP | 0.289 |
| 335 | **L** | 333 | 338 | TNLCPF | 0.358 |
| 336 | **C** | 334 | 339 | NLCPFG | 0.245 |
| 337 | **P** | 335 | 340 | LCPFGE | 0.264 |
| 338 | **F** | 336 | 341 | CPFGEV | 0.238 |
| 339 | **G** | 337 | 342 | PFGEVF | 0.384 |
| 340 | **E** | 338 | 343 | FGEVFN | 0.399 |
| 341 | **V** | 339 | 344 | GEVFNA | 0.466 |
| 342 | **F** | 340 | 345 | EVFNAT | 0.679 |
| 343 | **N** | 341 | 346 | VFNATR | 0.768 |
| 344 | **A** | 342 | 347 | FNATRF | 0.896 |
| 345 | **T** | 343 | 348 | NATRFA | 1.046 |
| 346 | **R** | 344 | 349 | ATRFAS | 0.871 |
| 347 | **F** | 345 | 350 | TRFASV | 0.64 |
| 348 | **A** | 346 | 351 | RFASVY | 0.695 |
| 349 | **S** | 347 | 352 | FASVYA | 0.359 |
| 350 | **V** | 348 | 353 | ASVYAW | 0.435 |
| 351 | **Y** | 349 | 354 | SVYAWN | 0.693 |
| 352 | **A** | 350 | 355 | VYAWNR | 1.013 |
| 353 | **W** | 351 | 356 | YAWNRK | 2.729 |
| 354 | **N** | 352 | 357 | AWNRKR | 3.411 |
| 355 | **R** | 353 | 358 | WNRKRI | 2.367 |
| 356 | **K** | 354 | 359 | NRKRIS | 3.017 |
| 357 | **R** | 355 | 360 | RKRISN | 3.017 |
| 358 | **I** | 356 | 361 | KRISNC | 0.826 |
| 359 | **S** | 357 | 362 | RISNCV | 0.306 |
| 360 | **N** | 358 | 363 | ISNCVA | 0.158 |
| 361 | **C** | 359 | 364 | SNCVAD | 0.377 |
| 362 | **V** | 360 | 365 | NCVADY | 0.44 |
| 363 | **A** | 361 | 366 | CVADYS | 0.367 |
| 364 | **D** | 362 | 367 | VADYSV | 0.508 |
| 365 | **Y** | 363 | 368 | ADYSVL | 0.564 |
| 366 | **S** | 364 | 369 | DYSVLY | 0.875 |
| 367 | **V** | 365 | 370 | YSVLYN | 0.843 |
| 368 | **L** | 366 | 371 | SVLYNS | 0.721 |
| 369 | **Y** | 367 | 372 | VLYNSA | 0.544 |
| 370 | **N** | 368 | 373 | LYNSAS | 0.981 |
| 371 | **S** | 369 | 374 | YNSASF | 1.03 |
| 372 | **A** | 370 | 375 | NSASFS | 0.881 |
| 373 | **S** | 371 | 376 | SASFST | 0.791 |
| 374 | **F** | 372 | 377 | ASFSTF | 0.511 |
| 375 | **S** | 373 | 378 | SFSTFK | 1.012 |
| 376 | **T** | 374 | 379 | FSTFKC | 0.405 |
| 377 | **F** | 375 | 380 | STFKCY | 0.732 |
| 378 | **K** | 376 | 381 | TFKCYG | 0.541 |
| 379 | **C** | 377 | 382 | FKCYGV | 0.278 |
| 380 | **Y** | 378 | 383 | KCYGVS | 0.43 |
| 381 | **G** | 379 | 384 | CYGVSP | 0.333 |
| 382 | **V** | 380 | 385 | YGVSPT | 0.896 |
| 383 | **S** | 381 | 386 | GVSPTK | 1.143 |
| 384 | **P** | 382 | 387 | VSPTKL | 0.953 |
| 385 | **T** | 383 | 388 | SPTKLN | 2.065 |
| 386 | **K** | 384 | 389 | PTKLND | 2.573 |
| 387 | **L** | 385 | 390 | TKLNDL | 1.372 |
| 388 | **N** | 386 | 391 | KLNDLC | 0.51 |
| 389 | **D** | 387 | 392 | LNDLCF | 0.221 |
| 390 | **L** | 388 | 393 | NDLCFT | 0.386 |
| 391 | **C** | 389 | 394 | DLCFTN | 0.386 |
| 392 | **F** | 390 | 395 | LCFTNV | 0.172 |
| 393 | **T** | 391 | 396 | CFTNVY | 0.326 |
| 394 | **N** | 392 | 397 | FTNVYA | 0.615 |
| 395 | **V** | 393 | 398 | TNVYAD | 1.185 |
| 396 | **Y** | 394 | 399 | NVYADS | 1.101 |
| 397 | **A** | 395 | 400 | VYADSF | 0.593 |
| 398 | **D** | 396 | 401 | YADSFV | 0.593 |
| 399 | **S** | 397 | 402 | ADSFVI | 0.265 |
| 400 | **F** | 398 | 403 | DSFVIR | 0.514 |
| 401 | **V** | 399 | 404 | SFVIRG | 0.305 |
| 402 | **I** | 400 | 405 | FVIRGD | 0.38 |
| 403 | **R** | 401 | 406 | VIRGDE | 0.759 |
| 404 | **G** | 402 | 407 | IRGDEV | 0.759 |
| 405 | **D** | 403 | 408 | RGDEVR | 2.121 |
| 406 | **E** | 404 | 409 | GDEVRQ | 1.876 |
| 407 | **V** | 405 | 410 | DEVRQI | 1.329 |
| 408 | **R** | 406 | 411 | EVRQIA | 0.804 |
| 409 | **Q** | 407 | 412 | VRQIAP | 0.718 |
| 410 | **I** | 408 | 413 | RQIAPG | 0.957 |
| 411 | **A** | 409 | 414 | QIAPGQ | 0.846 |
| 412 | **P** | 410 | 415 | IAPGQT | 0.705 |
| 413 | **G** | 411 | 416 | APGQTG | 0.995 |
| 414 | **Q** | 412 | 417 | PGQTGK | 1.97 |
| 415 | **T** | 413 | 418 | GQTGKI | 0.893 |
| 416 | **G** | 414 | 419 | QTGKIA | 0.912 |
| 417 | **K** | 415 | 420 | TGKIAD | 0.879 |
| 418 | **I** | 416 | 421 | GKIADY | 0.955 |
| 419 | **A** | 417 | 422 | KIADYN | 1.551 |
| 420 | **D** | 418 | 423 | IADYNY | 1.215 |
| 421 | **Y** | 419 | 424 | ADYNYK | 3.467 |
| 422 | **N** | 420 | 425 | DYNYKL | 2.83 |
| 423 | **Y** | 421 | 426 | YNYKLP | 2.621 |
| 424 | **K** | 422 | 427 | NYKLPD | 2.793 |
| 425 | **L** | 423 | 428 | YKLPDD | 2.901 |
| 426 | **P** | 424 | 429 | KLPDDF | 1.603 |
| 427 | **D** | 425 | 430 | LPDDFT | 1.157 |
| 428 | **D** | 426 | 431 | PDDFTG | 1.388 |
| 429 | **F** | 427 | 432 | DDFTGC | 0.481 |
| 430 | **T** | 428 | 433 | DFTGCV | 0.214 |
| 431 | **G** | 429 | 434 | FTGCVI | 0.09 |
| 432 | **C** | 430 | 435 | TGCVIA | 0.105 |
| 433 | **V** | 431 | 436 | GCVIAW | 0.076 |
| 434 | **I** | 432 | 437 | CVIAWN | 0.124 |
| 435 | **A** | 433 | 438 | VIAWNS | 0.31 |
| 436 | **W** | 434 | 439 | IAWNSN | 0.672 |
| 437 | **N** | 435 | 440 | AWNSNN | 1.541 |
| 438 | **S** | 436 | 441 | WNSNNL | 1.258 |
| 439 | **N** | 437 | 442 | NSNNLD | 1.998 |
| 440 | **N** | 438 | 443 | SNNLDS | 1.665 |
| 441 | **L** | 439 | 444 | NNLDSK | 2.485 |
| 442 | **D** | 440 | 445 | NLDSKV | 1.147 |
| 443 | **S** | 441 | 446 | LDSKVG | 0.706 |
| 444 | **K** | 442 | 447 | DSKVGG | 0.847 |
| 445 | **V** | 443 | 448 | SKVGGN | 0.815 |
| 446 | **G** | 444 | 449 | KVGGNY | 0.953 |
| 447 | **G** | 445 | 450 | VGGNYN | 0.767 |
| 448 | **N** | 446 | 451 | GGNYNY | 1.619 |
| 449 | **Y** | 447 | 452 | GNYNYL | 1.349 |
| 450 | **N** | 448 | 453 | NYNYLY | 2.136 |
| 451 | **Y** | 449 | 454 | YNYLYR | 2.601 |
| 452 | **L** | 450 | 455 | NYLYRL | 1.369 |
| 453 | **Y** | 451 | 456 | YLYRLF | 0.737 |
| 454 | **R** | 452 | 457 | LYRLFR | 0.921 |
| 455 | **L** | 453 | 458 | YRLFRK | 2.234 |
| 456 | **F** | 454 | 459 | RLFRKS | 1.911 |
| 457 | **R** | 455 | 460 | LFRKSN | 1.569 |
| 458 | **K** | 456 | 461 | FRKSNL | 1.569 |
| 459 | **S** | 457 | 462 | RKSNLK | 3.624 |
| 460 | **N** | 458 | 463 | KSNLKP | 2.861 |
| 461 | **L** | 459 | 464 | SNLKPF | 1.239 |
| 462 | **K** | 460 | 465 | NLKPFE | 1.601 |
| 463 | **P** | 461 | 466 | LKPFER | 1.95 |
| 464 | **F** | 462 | 467 | KPFERD | 3.948 |
| 465 | **E** | 463 | 468 | PFERDI | 1.384 |
| 466 | **R** | 464 | 469 | FERDIS | 1.199 |
| 467 | **D** | 465 | 470 | ERDIST | 1.999 |
| 468 | **I** | 466 | 471 | RDISTE | 1.999 |
| 469 | **S** | 467 | 472 | DISTEI | 0.715 |
| 470 | **T** | 468 | 473 | ISTEIY | 0.671 |
| 471 | **E** | 469 | 474 | STEIYQ | 1.658 |
| 472 | **I** | 470 | 475 | TEIYQA | 1.25 |
| 473 | **Y** | 471 | 476 | EIYQAG | 0.857 |
| 474 | **Q** | 472 | 477 | IYQAGS | 0.663 |
| 475 | **A** | 473 | 478 | YQAGST | 1.366 |
| 476 | **G** | 474 | 479 | QAGSTP | 1.348 |
| 477 | **S** | 475 | 480 | AGSTPC | 0.417 |
| 478 | **T** | 476 | 481 | GSTPCN | 0.664 |
| 479 | **P** | 477 | 482 | STPCNG | 0.664 |
| 480 | **C** | 478 | 483 | TPCNGV | 0.368 |
| 481 | **N** | 479 | 484 | PCNGVE | 0.441 |
| 482 | **G** | 480 | 485 | CNGVEG | 0.282 |
| 483 | **V** | 481 | 486 | NGVEGF | 0.456 |
| 484 | **E** | 482 | 487 | GVEGFN | 0.456 |
| 485 | **G** | 483 | 488 | VEGFNC | 0.247 |
| 486 | **F** | 484 | 489 | EGFNCY | 0.522 |
| 487 | **N** | 485 | 490 | GFNCYF | 0.261 |
| 488 | **C** | 486 | 491 | FNCYFP | 0.408 |
| 489 | **Y** | 487 | 492 | NCYFPL | 0.388 |
| 490 | **F** | 488 | 493 | CYFPLQ | 0.418 |
| 491 | **P** | 489 | 494 | YFPLQS | 1.045 |
| 492 | **L** | 490 | 495 | FPLQSY | 1.045 |
| 493 | **Q** | 491 | 496 | PLQSYG | 1.195 |
| 494 | **S** | 492 | 497 | LQSYGF | 0.669 |
| 495 | **Y** | 493 | 498 | QSYGFQ | 1.405 |
| 496 | **G** | 494 | 499 | SYGFQP | 1.254 |
| 497 | **F** | 495 | 500 | YGFQPT | 1.351 |
| 498 | **Q** | 496 | 501 | GFQPTN | 1.386 |
| 499 | **P** | 497 | 502 | FQPTNG | 1.386 |
| 500 | **T** | 498 | 503 | QPTNGV | 1.188 |
| 501 | **N** | 499 | 504 | PTNGVG | 0.679 |
| 502 | **G** | 500 | 505 | TNGVGY | 0.688 |
| 503 | **V** | 501 | 506 | NGVGYQ | 0.826 |
| 504 | **G** | 502 | 507 | GVGYQP | 0.794 |
| 505 | **Y** | 503 | 508 | VGYQPY | 1.257 |
| 506 | **Q** | 504 | 509 | GYQPYR | 3.317 |
| 507 | **P** | 505 | 510 | YQPYRV | 2.488 |
| 508 | **Y** | 506 | 511 | QPYRVV | 1.178 |
| 509 | **R** | 507 | 512 | PYRVVV | 0.505 |
| 510 | **V** | 508 | 513 | YRVVVL | 0.269 |
| 511 | **V** | 509 | 514 | RVVVLS | 0.23 |
| 512 | **V** | 510 | 515 | VVVLSF | 0.102 |
| 513 | **L** | 511 | 516 | VVLSFE | 0.238 |
| 514 | **S** | 512 | 517 | VLSFEL | 0.264 |
| 515 | **F** | 513 | 518 | LSFELL | 0.293 |
| 516 | **E** | 514 | 519 | SFELLH | 0.484 |
| 517 | **L** | 515 | 520 | FELLHA | 0.365 |
| 518 | **L** | 516 | 521 | ELLHAP | 0.652 |
| 519 | **H** | 517 | 522 | LLHAPA | 0.38 |
| 520 | **A** | 518 | 523 | LHAPAT | 0.665 |
| 521 | **P** | 519 | 524 | HAPATV | 0.599 |
| 522 | **A** | 520 | 525 | APATVC | 0.236 |
| 523 | **T** | 521 | 526 | PATVCG | 0.231 |
| 524 | **V** | 522 | 527 | ATVCGP | 0.231 |
| 525 | **C** | 523 | 528 | TVCGPK | 0.457 |
| 526 | **G** | 524 | 529 | VCGPKK | 0.634 |
| 527 | **P** | 525 | 530 | CGPKKS | 1.144 |
| 528 | **K** | 526 | 531 | GPKKST | 3.081 |
| 529 | **K** | 527 | 532 | PKKSTN | 5.007 |
| 530 | **S** | 528 | 533 | KKSTNL | 2.67 |
| 531 | **T** | 529 | 534 | KSTNLV | 0.991 |
| 532 | **N** | 530 | 535 | STNLVK | 0.991 |
| 533 | **L** | 531 | 536 | TNLVKN | 1.189 |
| 534 | **V** | 532 | 537 | NLVKNK | 1.648 |
| 535 | **K** | 533 | 538 | LVKNKC | 0.549 |
| 536 | **N** | 534 | 539 | VKNKCV | 0.494 |
| 537 | **K** | 535 | 540 | KNKCVN | 1.071 |
| 538 | **C** | 536 | 541 | NKCVNF | 0.464 |
| 539 | **V** | 537 | 542 | KCVNFN | 0.464 |
| 540 | **N** | 538 | 543 | CVNFNF | 0.201 |
| 541 | **F** | 539 | 544 | VNFNFN | 0.602 |
| 542 | **N** | 540 | 545 | NFNFNG | 0.803 |
| 543 | **F** | 541 | 546 | FNFNGL | 0.412 |
| 544 | **N** | 542 | 547 | NFNGLT | 0.687 |
| 545 | **G** | 543 | 548 | FNGLTG | 0.422 |
| 546 | **L** | 544 | 549 | NGLTGT | 0.704 |
| 547 | **T** | 545 | 550 | GLTGTG | 0.433 |
| 548 | **G** | 546 | 551 | LTGTGV | 0.325 |
| 549 | **T** | 547 | 552 | TGTGVL | 0.325 |
| 550 | **G** | 548 | 553 | GTGVLT | 0.325 |
| 551 | **V** | 549 | 554 | TGVLTE | 0.569 |
| 552 | **L** | 550 | 555 | GVLTES | 0.528 |
| 553 | **T** | 551 | 556 | VLTESN | 0.858 |
| 554 | **E** | 552 | 557 | LTESNK | 2.312 |
| 555 | **S** | 553 | 558 | TESNKK | 5.607 |
| 556 | **N** | 554 | 559 | ESNKKF | 3.364 |
| 557 | **K** | 555 | 560 | SNKKFL | 1.602 |
| 558 | **K** | 556 | 561 | NKKFLP | 1.849 |
| 559 | **F** | 557 | 562 | KKFLPF | 0.995 |
| 560 | **L** | 558 | 563 | KFLPFQ | 0.862 |
| 561 | **P** | 559 | 564 | FLPFQQ | 0.746 |
| 562 | **F** | 560 | 565 | LPFQQF | 0.746 |
| 563 | **Q** | 561 | 566 | PFQQFG | 0.896 |
| 564 | **Q** | 562 | 567 | FQQFGR | 1.135 |
| 565 | **F** | 563 | 568 | QQFGRD | 2.188 |
| 566 | **G** | 564 | 569 | QFGRDI | 0.886 |
| 567 | **R** | 565 | 570 | FGRDIA | 0.517 |
| 568 | **D** | 566 | 571 | GRDIAD | 0.996 |
| 569 | **I** | 567 | 572 | RDIADT | 1.453 |
| 570 | **A** | 568 | 573 | DIADTT | 1.071 |
| 571 | **D** | 569 | 574 | IADTTD | 1.071 |
| 572 | **T** | 570 | 575 | ADTTDA | 1.543 |
| 573 | **T** | 571 | 576 | DTTDAV | 1.134 |
| 574 | **D** | 572 | 577 | TTDAVR | 1.33 |
| 575 | **A** | 573 | 578 | TDAVRD | 1.539 |
| 576 | **V** | 574 | 579 | DAVRDP | 1.648 |
| 577 | **R** | 575 | 580 | AVRDPQ | 1.71 |
| 578 | **D** | 576 | 581 | VRDPQT | 2.442 |
| 579 | **P** | 577 | 582 | RDPQTL | 2.714 |
| 580 | **Q** | 578 | 583 | DPQTLE | 2.399 |
| 581 | **T** | 579 | 584 | PQTLEI | 1.007 |
| 582 | **L** | 580 | 585 | QTLEIL | 0.537 |
| 583 | **E** | 581 | 586 | TLEILD | 0.518 |
| 584 | **I** | 582 | 587 | LEILDI | 0.252 |
| 585 | **L** | 583 | 588 | EILDIT | 0.44 |
| 586 | **D** | 584 | 589 | ILDITP | 0.393 |
| 587 | **I** | 585 | 590 | LDITPC | 0.301 |
| 588 | **T** | 586 | 591 | DITPCS | 0.488 |
| 589 | **P** | 587 | 592 | ITPCSF | 0.253 |
| 590 | **C** | 588 | 593 | TPCSFG | 0.358 |
| 591 | **S** | 589 | 594 | PCSFGG | 0.245 |
| 592 | **F** | 590 | 595 | CSFGGV | 0.118 |
| 593 | **G** | 591 | 596 | SFGGVS | 0.294 |
| 594 | **G** | 592 | 597 | FGGVSV | 0.163 |
| 595 | **V** | 593 | 598 | GGVSVI | 0.132 |
| 596 | **S** | 594 | 599 | GVSVIT | 0.192 |
| 597 | **V** | 595 | 600 | VSVITP | 0.301 |
| 598 | **I** | 596 | 601 | SVITPG | 0.401 |
| 599 | **T** | 597 | 602 | VITPGT | 0.432 |
| 600 | **P** | 598 | 603 | ITPGTN | 0.935 |
| 601 | **G** | 599 | 604 | TPGTNT | 1.925 |
| 602 | **T** | 600 | 605 | PGTNTS | 1.788 |
| 603 | **N** | 601 | 606 | GTNTSN | 1.859 |
| 604 | **T** | 602 | 607 | TNTSNQ | 3.254 |
| 605 | **S** | 603 | 608 | NTSNQV | 1.673 |
| 606 | **N** | 604 | 609 | TSNQVA | 1.051 |
| 607 | **Q** | 605 | 610 | SNQVAV | 0.541 |
| 608 | **V** | 606 | 611 | NQVAVL | 0.333 |
| 609 | **A** | 607 | 612 | QVAVLY | 0.324 |
| 610 | **V** | 608 | 613 | VAVLYQ | 0.324 |
| 611 | **L** | 609 | 614 | AVLYQD | 0.729 |
| 612 | **Y** | 610 | 615 | VLYQDV | 0.536 |
| 613 | **Q** | 611 | 616 | LYQDVN | 1.161 |
| 614 | **D** | 612 | 617 | YQDVNC | 0.755 |
| 615 | **V** | 613 | 618 | QDVNCT | 0.695 |
| 616 | **N** | 614 | 619 | DVNCTE | 0.695 |
| 617 | **C** | 615 | 620 | VNCTEV | 0.309 |
| 618 | **T** | 616 | 621 | NCTEVP | 0.644 |
| 619 | **E** | 617 | 622 | CTEVPV | 0.297 |
| 620 | **V** | 618 | 623 | TEVPVA | 0.56 |
| 621 | **P** | 619 | 624 | EVPVAI | 0.272 |
| 622 | **V** | 620 | 625 | VPVAIH | 0.214 |
| 623 | **A** | 621 | 626 | PVAIHA | 0.291 |
| 624 | **I** | 622 | 627 | VAIHAD | 0.314 |
| 625 | **H** | 623 | 628 | AIHADQ | 0.733 |
| 626 | **A** | 624 | 629 | IHADQL | 0.598 |
| 627 | **D** | 625 | 630 | HADQLT | 1.232 |
| 628 | **Q** | 626 | 631 | ADQLTP | 1.4 |
| 629 | **L** | 627 | 632 | DQLTPT | 1.999 |
| 630 | **T** | 628 | 633 | QLTPTW | 1.259 |
| 631 | **P** | 629 | 634 | LTPTWR | 1.424 |
| 632 | **T** | 630 | 635 | TPTWRV | 1.281 |
| 633 | **W** | 631 | 636 | PTWRVY | 1.391 |
| 634 | **R** | 632 | 637 | TWRVYS | 1.206 |
| 635 | **V** | 633 | 638 | WRVYST | 1.206 |
| 636 | **Y** | 634 | 639 | RVYSTG | 1.135 |
| 637 | **S** | 635 | 640 | VYSTGS | 0.776 |
| 638 | **T** | 636 | 641 | YSTGSN | 1.682 |
| 639 | **G** | 637 | 642 | STGSNV | 0.797 |
| 640 | **S** | 638 | 643 | TGSNVF | 0.515 |
| 641 | **N** | 639 | 644 | GSNVFQ | 0.618 |
| 642 | **V** | 640 | 645 | SNVFQT | 0.901 |
| 643 | **F** | 641 | 646 | NVFQTR | 1.317 |
| 644 | **Q** | 642 | 647 | VFQTRA | 0.827 |
| 645 | **T** | 643 | 648 | FQTRAG | 1.103 |
| 646 | **R** | 644 | 649 | QTRAGC | 0.683 |
| 647 | **A** | 645 | 650 | TRAGCL | 0.325 |
| 648 | **G** | 646 | 651 | RAGCLI | 0.158 |
| 649 | **C** | 647 | 652 | AGCLIG | 0.08 |
| 650 | **L** | 648 | 653 | GCLIGA | 0.08 |
| 651 | **I** | 649 | 654 | CLIGAE | 0.14 |
| 652 | **G** | 650 | 655 | LIGAEH | 0.355 |
| 653 | **A** | 651 | 656 | IGAEHV | 0.319 |
| 654 | **E** | 652 | 657 | GAEHVN | 0.732 |
| 655 | **H** | 653 | 658 | AEHVNN | 1.189 |
| 656 | **V** | 654 | 659 | EHVNNS | 1.578 |
| 657 | **N** | 655 | 660 | HVNNSY | 1.428 |
| 658 | **N** | 656 | 661 | VNNSYE | 1.817 |
| 659 | **S** | 657 | 662 | NNSYEC | 1.312 |
| 660 | **Y** | 658 | 663 | NSYECD | 1.363 |
| 661 | **E** | 659 | 664 | SYECDI | 0.594 |
| 662 | **C** | 660 | 665 | YECDIP | 0.685 |
| 663 | **D** | 661 | 666 | ECDIPI | 0.307 |
| 664 | **I** | 662 | 667 | CDIPIG | 0.175 |
| 665 | **P** | 663 | 668 | DIPIGA | 0.33 |
| 666 | **I** | 664 | 669 | IPIGAG | 0.196 |
| 667 | **G** | 665 | 670 | PIGAGI | 0.196 |
| 668 | **A** | 666 | 671 | IGAGIC | 0.068 |
| 669 | **G** | 667 | 672 | GAGICA | 0.098 |
| 670 | **I** | 668 | 673 | AGICAS | 0.132 |
| 671 | **C** | 669 | 674 | GICASY | 0.205 |
| 672 | **A** | 670 | 675 | ICASYQ | 0.359 |
| 673 | **S** | 671 | 676 | CASYQT | 0.74 |
| 674 | **Y** | 672 | 677 | ASYQTQ | 2.39 |
| 675 | **Q** | 673 | 678 | SYQTQT | 3.414 |
| 676 | **T** | 674 | 679 | YQTQTN | 4.097 |
| 677 | **Q** | 675 | 680 | QTQTNS | 3.504 |
| 678 | **T** | 676 | 681 | TQTNSP | 3.129 |
| 679 | **N** | 677 | 682 | QTNSPR | 4.246 |
| 680 | **S** | 678 | 683 | TNSPRR | 4.802 |
| 681 | **P** | 679 | 684 | NSPRRA | 3.362 |
| 682 | **R** | 680 | 685 | SPRRAR | 4.094 |
| 683 | **R** | 681 | 686 | PRRARS | 4.094 |
| 684 | **A** | 682 | 687 | RRARSV | 1.965 |
| 685 | **R** | 683 | 688 | RARSVA | 1.014 |
| 686 | **S** | 684 | 689 | ARSVAS | 0.694 |
| 687 | **V** | 685 | 690 | RSVASQ | 1.189 |
| 688 | **A** | 686 | 691 | SVASQS | 0.813 |
| 689 | **S** | 687 | 692 | VASQSI | 0.426 |
| 690 | **Q** | 688 | 693 | ASQSII | 0.402 |
| 691 | **S** | 689 | 694 | SQSIIA | 0.402 |
| 692 | **I** | 690 | 695 | QSIIAY | 0.47 |
| 693 | **I** | 691 | 696 | SIIAYT | 0.392 |
| 694 | **A** | 692 | 697 | IIAYTM | 0.289 |
| 695 | **Y** | 693 | 698 | IAYTMS | 0.553 |
| 696 | **T** | 694 | 699 | AYTMSL | 0.65 |
| 697 | **M** | 695 | 700 | YTMSLG | 0.637 |
| 698 | **S** | 696 | 701 | TMSLGA | 0.411 |
| 699 | **L** | 697 | 702 | MSLGAE | 0.493 |
| 700 | **G** | 698 | 703 | SLGAEN | 0.801 |
| 701 | **A** | 699 | 704 | LGAENS | 0.801 |
| 702 | **E** | 700 | 705 | GAENSV | 0.721 |
| 703 | **N** | 701 | 706 | AENSVA | 0.736 |
| 704 | **S** | 702 | 707 | ENSVAY | 1.141 |
| 705 | **V** | 703 | 708 | NSVAYS | 0.883 |
| 706 | **A** | 704 | 709 | SVAYSN | 0.883 |
| 707 | **Y** | 705 | 710 | VAYSNN | 1.06 |
| 708 | **S** | 706 | 711 | AYSNNS | 1.914 |
| 709 | **N** | 707 | 712 | YSNNSI | 1.328 |
| 710 | **N** | 708 | 713 | SNNSIA | 0.856 |
| 711 | **S** | 709 | 714 | NNSIAI | 0.448 |
| 712 | **I** | 710 | 715 | NSIAIP | 0.431 |
| 713 | **A** | 711 | 716 | SIAIPT | 0.386 |
| 714 | **I** | 712 | 717 | IAIPTN | 0.464 |
| 715 | **P** | 713 | 718 | AIPTNF | 0.573 |
| 716 | **T** | 714 | 719 | IPTNFT | 0.818 |
| 717 | **N** | 715 | 720 | PTNFTI | 0.818 |
| 718 | **F** | 716 | 721 | TNFTIS | 0.709 |
| 719 | **T** | 717 | 722 | NFTISV | 0.365 |
| 720 | **I** | 718 | 723 | FTISVT | 0.327 |
| 721 | **S** | 719 | 724 | TISVTT | 0.546 |
| 722 | **V** | 720 | 725 | ISVTTE | 0.655 |
| 723 | **T** | 721 | 726 | SVTTEI | 0.655 |
| 724 | **T** | 722 | 727 | VTTEIL | 0.403 |
| 725 | **E** | 723 | 728 | TTEILP | 0.839 |
| 726 | **I** | 724 | 729 | TEILPV | 0.432 |
| 727 | **L** | 725 | 730 | EILPVS | 0.401 |
| 728 | **P** | 726 | 731 | ILPVSM | 0.229 |
| 729 | **V** | 727 | 732 | LPVSMT | 0.472 |
| 730 | **S** | 728 | 733 | PVSMTK | 1.143 |
| 731 | **M** | 729 | 734 | VSMTKT | 1.067 |
| 732 | **T** | 730 | 735 | SMTKTS | 1.927 |
| 733 | **K** | 731 | 736 | MTKTSV | 1.067 |
| 734 | **T** | 732 | 737 | TKTSVD | 1.801 |
| 735 | **S** | 733 | 738 | KTSVDC | 0.669 |
| 736 | **V** | 734 | 739 | TSVDCT | 0.483 |
| 737 | **D** | 735 | 740 | SVDCTM | 0.331 |
| 738 | **C** | 736 | 741 | VDCTMY | 0.387 |
| 739 | **T** | 737 | 742 | DCTMYI | 0.366 |
| 740 | **M** | 738 | 743 | CTMYIC | 0.117 |
| 741 | **Y** | 739 | 744 | TMYICG | 0.217 |
| 742 | **I** | 740 | 745 | MYICGD | 0.251 |
| 743 | **C** | 741 | 746 | YICGDS | 0.339 |
| 744 | **G** | 742 | 747 | ICGDST | 0.313 |
| 745 | **D** | 743 | 748 | CGDSTE | 0.772 |
| 746 | **S** | 744 | 749 | GDSTEC | 0.772 |
| 747 | **T** | 745 | 750 | DSTECS | 1.046 |
| 748 | **E** | 746 | 751 | STECSN | 1.007 |
| 749 | **C** | 747 | 752 | TECSNL | 0.62 |
| 750 | **S** | 748 | 753 | ECSNLL | 0.354 |
| 751 | **N** | 749 | 754 | CSNLLL | 0.169 |
| 752 | **L** | 750 | 755 | SNLLLQ | 0.545 |
| 753 | **L** | 751 | 756 | NLLLQY | 0.637 |
| 754 | **L** | 752 | 757 | LLLQYG | 0.392 |
| 755 | **Q** | 753 | 758 | LLQYGS | 0.637 |
| 756 | **Y** | 754 | 759 | LQYGSF | 0.669 |
| 757 | **G** | 755 | 760 | QYGSFC | 0.435 |
| 758 | **S** | 756 | 761 | YGSFCT | 0.362 |
| 759 | **F** | 757 | 762 | GSFCTQ | 0.4 |
| 760 | **C** | 758 | 763 | SFCTQL | 0.334 |
| 761 | **T** | 759 | 764 | FCTQLN | 0.4 |
| 762 | **Q** | 760 | 765 | CTQLNR | 0.906 |
| 763 | **L** | 761 | 766 | TQLNRA | 1.707 |
| 764 | **N** | 762 | 767 | QLNRAL | 0.976 |
| 765 | **R** | 763 | 768 | LNRALT | 0.813 |
| 766 | **A** | 764 | 769 | NRALTG | 0.976 |
| 767 | **L** | 765 | 770 | RALTGI | 0.425 |
| 768 | **T** | 766 | 771 | ALTGIA | 0.219 |
| 769 | **G** | 767 | 772 | LTGIAV | 0.161 |
| 770 | **I** | 768 | 773 | TGIAVE | 0.338 |
| 771 | **A** | 769 | 774 | GIAVEQ | 0.406 |
| 772 | **V** | 770 | 775 | IAVEQD | 0.685 |
| 773 | **E** | 771 | 776 | AVEQDK | 1.955 |
| 774 | **Q** | 772 | 777 | VEQDKN | 3.112 |
| 775 | **D** | 773 | 778 | EQDKNT | 6.051 |
| 776 | **K** | 774 | 779 | QDKNTQ | 6.051 |
| 777 | **N** | 775 | 780 | DKNTQE | 6.051 |
| 778 | **T** | 776 | 781 | KNTQEV | 2.689 |
| 779 | **Q** | 777 | 782 | NTQEVF | 1.164 |
| 780 | **E** | 778 | 783 | TQEVFA | 0.732 |
| 781 | **V** | 779 | 784 | QEVFAQ | 0.878 |
| 782 | **F** | 780 | 785 | EVFAQV | 0.376 |
| 783 | **A** | 781 | 786 | VFAQVK | 0.434 |
| 784 | **Q** | 782 | 787 | FAQVKQ | 1.014 |
| 785 | **V** | 783 | 788 | AQVKQI | 0.821 |
| 786 | **K** | 784 | 789 | QVKQIY | 1.273 |
| 787 | **Q** | 785 | 790 | VKQIYK | 1.47 |
| 788 | **I** | 786 | 791 | KQIYKT | 2.858 |
| 789 | **Y** | 787 | 792 | QIYKTP | 2.21 |
| 790 | **K** | 788 | 793 | IYKTPP | 1.973 |
| 791 | **T** | 789 | 794 | YKTPPI | 1.973 |
| 792 | **P** | 790 | 795 | KTPPIK | 2.518 |
| 793 | **P** | 791 | 796 | TPPIKD | 2.103 |
| 794 | **I** | 792 | 797 | PPIKDF | 1.262 |
| 795 | **K** | 793 | 798 | PIKDFG | 0.807 |
| 796 | **D** | 794 | 799 | IKDFGG | 0.517 |
| 797 | **F** | 795 | 800 | KDFGGF | 0.638 |
| 798 | **G** | 796 | 801 | DFGGFN | 0.513 |
| 799 | **G** | 797 | 802 | FGGFNF | 0.266 |
| 800 | **F** | 798 | 803 | GGFNFS | 0.412 |
| 801 | **N** | 799 | 804 | GFNFSQ | 0.721 |
| 802 | **F** | 800 | 805 | FNFSQI | 0.511 |
| 803 | **S** | 801 | 806 | NFSQIL | 0.486 |
| 804 | **Q** | 802 | 807 | FSQILP | 0.468 |
| 805 | **I** | 803 | 808 | SQILPD | 0.902 |
| 806 | **L** | 804 | 809 | QILPDP | 1.041 |
| 807 | **P** | 805 | 810 | ILPDPS | 0.805 |
| 808 | **D** | 806 | 811 | LPDPSK | 2.297 |
| 809 | **P** | 807 | 812 | PDPSKP | 4.307 |
| 810 | **S** | 808 | 813 | DPSKPS | 3.733 |
| 811 | **K** | 809 | 814 | PSKPSK | 4.47 |
| 812 | **P** | 810 | 815 | SKPSKR | 5.662 |
| 813 | **S** | 811 | 816 | KPSKRS | 5.662 |
| 814 | **K** | 812 | 817 | PSKRSF | 2.452 |
| 815 | **R** | 813 | 818 | SKRSFI | 1.111 |
| 816 | **S** | 814 | 819 | KRSFIE | 1.436 |
| 817 | **F** | 815 | 820 | RSFIED | 1.199 |
| 818 | **I** | 816 | 821 | SFIEDL | 0.505 |
| 819 | **E** | 817 | 822 | FIEDLL | 0.311 |
| 820 | **D** | 818 | 823 | IEDLLF | 0.311 |
| 821 | **L** | 819 | 824 | EDLLFN | 0.713 |
| 822 | **L** | 820 | 825 | DLLFNK | 0.823 |
| 823 | **F** | 821 | 826 | LLFNKV | 0.366 |
| 824 | **N** | 822 | 827 | LFNKVT | 0.64 |
| 825 | **K** | 823 | 828 | FNKVTL | 0.64 |
| 826 | **V** | 824 | 829 | NKVTLA | 0.747 |
| 827 | **T** | 825 | 830 | KVTLAD | 0.776 |
| 828 | **L** | 826 | 831 | VTLADA | 0.392 |
| 829 | **A** | 827 | 832 | TLADAG | 0.523 |
| 830 | **D** | 828 | 833 | LADAGF | 0.314 |
| 831 | **A** | 829 | 834 | ADAGFI | 0.266 |
| 832 | **G** | 830 | 835 | DAGFIK | 0.528 |
| 833 | **F** | 831 | 836 | AGFIKQ | 0.547 |
| 834 | **I** | 832 | 837 | GFIKQY | 0.849 |
| 835 | **K** | 833 | 838 | FIKQYG | 0.849 |
| 836 | **Q** | 834 | 839 | IKQYGD | 1.636 |
| 837 | **Y** | 835 | 840 | KQYGDC | 1.251 |
| 838 | **G** | 836 | 841 | QYGDCL | 0.516 |
| 839 | **D** | 837 | 842 | YGDCLG | 0.295 |
| 840 | **C** | 838 | 843 | GDCLGD | 0.314 |
| 841 | **L** | 839 | 844 | DCLGDI | 0.223 |
| 842 | **G** | 840 | 845 | CLGDIA | 0.135 |
| 843 | **D** | 841 | 846 | LGDIAA | 0.254 |
| 844 | **I** | 842 | 847 | GDIAAR | 0.603 |
| 845 | **A** | 843 | 848 | DIAARD | 1.017 |
| 846 | **A** | 844 | 849 | IAARDL | 0.502 |
| 847 | **R** | 845 | 850 | AARDLI | 0.502 |
| 848 | **D** | 846 | 851 | ARDLIC | 0.267 |
| 849 | **L** | 847 | 852 | RDLICA | 0.267 |
| 850 | **I** | 848 | 853 | DLICAQ | 0.236 |
| 851 | **C** | 849 | 854 | LICAQK | 0.282 |
| 852 | **A** | 850 | 855 | ICAQKF | 0.296 |
| 853 | **Q** | 851 | 856 | CAQKFN | 0.68 |
| 854 | **K** | 852 | 857 | AQKFNG | 1.255 |
| 855 | **F** | 853 | 858 | QKFNGL | 1.025 |
| 856 | **N** | 854 | 859 | KFNGLT | 0.854 |
| 857 | **G** | 855 | 860 | FNGLTV | 0.317 |
| 858 | **L** | 856 | 861 | NGLTVL | 0.302 |
| 859 | **T** | 857 | 862 | GLTVLP | 0.29 |
| 860 | **V** | 858 | 863 | LTVLPP | 0.453 |
| 861 | **L** | 859 | 864 | TVLPPL | 0.453 |
| 862 | **P** | 860 | 865 | VLPPLL | 0.259 |
| 863 | **P** | 861 | 866 | LPPLLT | 0.504 |
| 864 | **L** | 862 | 867 | PPLLTD | 1.02 |
| 865 | **L** | 863 | 868 | PLLTDE | 1.143 |
| 866 | **T** | 864 | 869 | LLTDEM | 0.731 |
| 867 | **D** | 865 | 870 | LTDEMI | 0.622 |
| 868 | **E** | 866 | 871 | TDEMIA | 0.761 |
| 869 | **M** | 867 | 872 | DEMIAQ | 0.914 |
| 870 | **I** | 868 | 873 | EMIAQY | 0.857 |
| 871 | **A** | 869 | 874 | MIAQYT | 0.714 |
| 872 | **Q** | 870 | 875 | IAQYTS | 0.967 |
| 873 | **Y** | 871 | 876 | AQYTSA | 1.394 |
| 874 | **T** | 872 | 877 | QYTSAL | 1.138 |
| 875 | **S** | 873 | 878 | YTSALL | 0.542 |
| 876 | **A** | 874 | 879 | TSALLA | 0.349 |
| 877 | **L** | 875 | 880 | SALLAG | 0.24 |
| 878 | **L** | 876 | 881 | ALLAGT | 0.258 |
| 879 | **A** | 877 | 882 | LLAGTI | 0.179 |
| 880 | **G** | 878 | 883 | LAGTIT | 0.313 |
| 881 | **T** | 879 | 884 | AGTITS | 0.509 |
| 882 | **I** | 880 | 885 | GTITSG | 0.499 |
| 883 | **T** | 881 | 886 | TITSGW | 0.53 |
| 884 | **S** | 882 | 887 | ITSGWT | 0.53 |
| 885 | **G** | 883 | 888 | TSGWTF | 0.655 |
| 886 | **W** | 884 | 889 | SGWTFG | 0.449 |
| 887 | **T** | 885 | 890 | GWTFGA | 0.338 |
| 888 | **F** | 886 | 891 | WTFGAG | 0.338 |
| 889 | **G** | 887 | 892 | TFGAGA | 0.325 |
| 890 | **A** | 888 | 893 | FGAGAA | 0.228 |
| 891 | **G** | 889 | 894 | GAGAAL | 0.217 |
| 892 | **A** | 890 | 895 | AGAALQ | 0.379 |
| 893 | **A** | 891 | 896 | GAALQI | 0.263 |
| 894 | **L** | 892 | 897 | AALQIP | 0.411 |
| 895 | **Q** | 893 | 898 | ALQIPF | 0.352 |
| 896 | **I** | 894 | 899 | LQIPFA | 0.352 |
| 897 | **P** | 895 | 900 | QIPFAM | 0.423 |
| 898 | **F** | 896 | 901 | IPFAMQ | 0.423 |
| 899 | **A** | 897 | 902 | PFAMQM | 0.597 |
| 900 | **M** | 898 | 903 | FAMQMA | 0.39 |
| 901 | **Q** | 899 | 904 | AMQMAY | 0.706 |
| 902 | **M** | 900 | 905 | MQMAYR | 1.369 |
| 903 | **A** | 901 | 906 | QMAYRF | 1.198 |
| 904 | **Y** | 902 | 907 | MAYRFN | 1.112 |
| 905 | **R** | 903 | 908 | AYRFNG | 1.112 |
| 906 | **F** | 904 | 909 | YRFNGI | 0.772 |
| 907 | **N** | 905 | 910 | RFNGIG | 0.487 |
| 908 | **G** | 906 | 911 | FNGIGV | 0.185 |
| 909 | **I** | 907 | 912 | NGIGVT | 0.308 |
| 910 | **G** | 908 | 913 | GIGVTQ | 0.331 |
| 911 | **V** | 909 | 914 | IGVTQN | 0.539 |
| 912 | **T** | 910 | 915 | GVTQNV | 0.57 |
| 913 | **Q** | 911 | 916 | VTQNVL | 0.475 |
| 914 | **N** | 912 | 917 | TQNVLY | 1.003 |
| 915 | **V** | 913 | 918 | QNVLYE | 1.204 |
| 916 | **L** | 914 | 919 | NVLYEN | 1.118 |
| 917 | **Y** | 915 | 920 | VLYENQ | 1.204 |
| 918 | **E** | 916 | 921 | LYENQK | 3.244 |
| 919 | **N** | 917 | 922 | YENQKL | 3.244 |
| 920 | **Q** | 918 | 923 | ENQKLI | 1.451 |
| 921 | **K** | 919 | 924 | NQKLIA | 0.847 |
| 922 | **L** | 920 | 925 | QKLIAN | 0.847 |
| 923 | **I** | 921 | 926 | KLIANQ | 0.847 |
| 924 | **A** | 922 | 927 | LIANQF | 0.367 |
| 925 | **N** | 923 | 928 | IANQFN | 0.715 |
| 926 | **Q** | 924 | 929 | ANQFNS | 1.367 |
| 927 | **F** | 925 | 930 | NQFNSA | 1.367 |
| 928 | **N** | 926 | 931 | QFNSAI | 0.596 |
| 929 | **S** | 927 | 932 | FNSAIG | 0.34 |
| 930 | **A** | 928 | 933 | NSAIGK | 0.786 |
| 931 | **I** | 929 | 934 | SAIGKI | 0.343 |
| 932 | **G** | 930 | 935 | AIGKIQ | 0.443 |
| 933 | **K** | 931 | 936 | IGKIQD | 0.732 |
| 934 | **I** | 932 | 937 | GKIQDS | 1.4 |
| 935 | **Q** | 933 | 938 | KIQDSL | 1.166 |
| 936 | **D** | 934 | 939 | IQDSLS | 0.782 |
| 937 | **S** | 935 | 940 | QDSLSS | 1.494 |
| 938 | **L** | 936 | 941 | DSLSST | 1.245 |
| 939 | **S** | 937 | 942 | SLSSTA | 0.753 |
| 940 | **S** | 938 | 943 | LSSTAS | 0.753 |
| 941 | **T** | 939 | 944 | SSTASA | 0.923 |
| 942 | **A** | 940 | 945 | STASAL | 0.568 |
| 943 | **S** | 941 | 946 | TASALG | 0.419 |
| 944 | **A** | 942 | 947 | ASALGK | 0.581 |
| 945 | **L** | 943 | 948 | SALGKL | 0.474 |
| 946 | **G** | 944 | 949 | ALGKLQ | 0.613 |
| 947 | **K** | 945 | 950 | LGKLQD | 1.013 |
| 948 | **L** | 946 | 951 | GKLQDV | 0.912 |
| 949 | **Q** | 947 | 952 | KLQDVV | 0.684 |
| 950 | **D** | 948 | 953 | LQDVVN | 0.55 |
| 951 | **V** | 949 | 954 | QDVVNQ | 1.155 |
| 952 | **V** | 950 | 955 | DVVNQN | 1.072 |
| 953 | **N** | 951 | 956 | VVNQNA | 0.649 |
| 954 | **Q** | 952 | 957 | VNQNAQ | 1.514 |
| 955 | **N** | 953 | 958 | NQNAQA | 2.06 |
| 956 | **A** | 954 | 959 | QNAQAL | 1.057 |
| 957 | **Q** | 955 | 960 | NAQALN | 0.981 |
| 958 | **A** | 956 | 961 | AQALNT | 0.881 |
| 959 | **L** | 957 | 962 | QALNTL | 0.719 |
| 960 | **N** | 958 | 963 | ALNTLV | 0.308 |
| 961 | **T** | 959 | 964 | LNTLVK | 0.61 |
| 962 | **L** | 960 | 965 | NTLVKQ | 1.281 |
| 963 | **V** | 961 | 966 | TLVKQL | 0.657 |
| 964 | **K** | 962 | 967 | LVKQLS | 0.61 |
| 965 | **Q** | 963 | 968 | VKQLSS | 0.991 |
| 966 | **L** | 964 | 969 | KQLSSN | 2.147 |
| 967 | **S** | 965 | 970 | QLSSNF | 0.93 |
| 968 | **S** | 966 | 971 | LSSNFG | 0.531 |
| 969 | **N** | 967 | 972 | SSNFGA | 0.651 |
| 970 | **F** | 968 | 973 | SNFGAI | 0.34 |
| 971 | **G** | 969 | 974 | NFGAIS | 0.34 |
| 972 | **A** | 970 | 975 | FGAISS | 0.284 |
| 973 | **I** | 971 | 976 | GAISSV | 0.243 |
| 974 | **S** | 972 | 977 | AISSVL | 0.203 |
| 975 | **S** | 973 | 978 | ISSVLN | 0.323 |
| 976 | **V** | 974 | 979 | SSVLND | 0.768 |
| 977 | **L** | 975 | 980 | SVLNDI | 0.402 |
| 978 | **N** | 976 | 981 | VLNDIL | 0.247 |
| 979 | **D** | 977 | 982 | LNDILS | 0.447 |
| 980 | **I** | 978 | 983 | NDILSR | 1.061 |
| 981 | **L** | 979 | 984 | DILSRL | 0.544 |
| 982 | **S** | 980 | 985 | ILSRLD | 0.544 |
| 983 | **R** | 981 | 986 | LSRLDK | 1.552 |
| 984 | **L** | 982 | 987 | SRLDKV | 1.397 |
| 985 | **D** | 983 | 988 | RLDKVE | 1.805 |
| 986 | **K** | 984 | 989 | LDKVEA | 0.931 |
| 987 | **V** | 985 | 990 | DKVEAE | 1.955 |
| 988 | **E** | 986 | 991 | KVEAEV | 0.869 |
| 989 | **A** | 987 | 992 | VEAEVQ | 0.752 |
| 990 | **E** | 988 | 993 | EAEVQI | 0.711 |
| 991 | **V** | 989 | 994 | AEVQID | 0.685 |
| 992 | **Q** | 990 | 995 | EVQIDR | 1.329 |
| 993 | **I** | 991 | 996 | VQIDRL | 0.633 |
| 994 | **D** | 992 | 997 | QIDRLI | 0.597 |
| 995 | **R** | 993 | 998 | IDRLIT | 0.498 |
| 996 | **L** | 994 | 999 | DRLITG | 0.703 |
| 997 | **I** | 995 | 1000 | RLITGR | 0.824 |
| 998 | **T** | 996 | 1001 | LITGRL | 0.347 |
| 999 | **G** | 997 | 1002 | ITGRLQ | 0.729 |
| 1000 | **R** | 998 | 1003 | TGRLQS | 1.394 |
| 1001 | **L** | 999 | 1004 | GRLQSL | 0.796 |
| 1002 | **Q** | 1000 | 1005 | RLQSLQ | 1.394 |
| 1003 | **S** | 1001 | 1006 | LQSLQT | 1.027 |
| 1004 | **L** | 1002 | 1007 | QSLQTY | 1.951 |
| 1005 | **Q** | 1003 | 1008 | SLQTYV | 0.836 |
| 1006 | **T** | 1004 | 1009 | LQTYVT | 0.9 |
| 1007 | **Y** | 1005 | 1010 | QTYVTQ | 1.891 |
| 1008 | **V** | 1006 | 1011 | TYVTQQ | 1.891 |
| 1009 | **T** | 1007 | 1012 | YVTQQL | 1.081 |
| 1010 | **Q** | 1008 | 1013 | VTQQLI | 0.483 |
| 1011 | **Q** | 1009 | 1014 | TQQLIR | 1.276 |
| 1012 | **L** | 1010 | 1015 | QQLIRA | 0.893 |
| 1013 | **I** | 1011 | 1016 | QLIRAA | 0.521 |
| 1014 | **R** | 1012 | 1017 | LIRAAE | 0.521 |
| 1015 | **A** | 1013 | 1018 | IRAAEI | 0.443 |
| 1016 | **A** | 1014 | 1019 | RAAEIR | 1.237 |
| 1017 | **E** | 1015 | 1020 | AAEIRA | 0.638 |
| 1018 | **I** | 1016 | 1021 | AEIRAS | 0.846 |
| 1019 | **R** | 1017 | 1022 | EIRASA | 0.846 |
| 1020 | **A** | 1018 | 1023 | IRASAN | 0.786 |
| 1021 | **S** | 1019 | 1024 | RASANL | 0.925 |
| 1022 | **A** | 1020 | 1025 | ASANLA | 0.477 |
| 1023 | **N** | 1021 | 1026 | SANLAA | 0.477 |
| 1024 | **L** | 1022 | 1027 | ANLAAT | 0.514 |
| 1025 | **A** | 1023 | 1028 | NLAATK | 1.017 |
| 1026 | **A** | 1024 | 1029 | LAATKM | 0.626 |
| 1027 | **T** | 1025 | 1030 | AATKMS | 1.017 |
| 1028 | **K** | 1026 | 1031 | ATKMSE | 1.743 |
| 1029 | **M** | 1027 | 1032 | TKMSEC | 0.925 |
| 1030 | **S** | 1028 | 1033 | KMSECV | 0.476 |
| 1031 | **E** | 1029 | 1034 | MSECVL | 0.196 |
| 1032 | **C** | 1030 | 1035 | SECVLG | 0.196 |
| 1033 | **V** | 1031 | 1036 | ECVLGQ | 0.253 |
| 1034 | **L** | 1032 | 1037 | CVLGQS | 0.196 |
| 1035 | **G** | 1033 | 1038 | VLGQSK | 0.732 |
| 1036 | **Q** | 1034 | 1039 | LGQSKR | 1.931 |
| 1037 | **S** | 1035 | 1040 | GQSKRV | 1.738 |
| 1038 | **K** | 1036 | 1041 | QSKRVD | 2.933 |
| 1039 | **R** | 1037 | 1042 | SKRVDF | 1.466 |
| 1040 | **V** | 1038 | 1043 | KRVDFC | 0.587 |
| 1041 | **D** | 1039 | 1044 | RVDFCG | 0.29 |
| 1042 | **F** | 1040 | 1045 | VDFCGK | 0.296 |
| 1043 | **C** | 1041 | 1046 | DFCGKG | 0.395 |
| 1044 | **G** | 1042 | 1047 | FCGKGY | 0.371 |
| 1045 | **K** | 1043 | 1048 | CGKGYH | 0.583 |
| 1046 | **G** | 1044 | 1049 | GKGYHL | 0.896 |
| 1047 | **Y** | 1045 | 1050 | KGYHLM | 0.896 |
| 1048 | **H** | 1046 | 1051 | GYHLMS | 0.601 |
| 1049 | **L** | 1047 | 1052 | YHLMSF | 0.526 |
| 1050 | **M** | 1048 | 1053 | HLMSFP | 0.519 |
| 1051 | **S** | 1049 | 1054 | LMSFPQ | 0.66 |
| 1052 | **F** | 1050 | 1055 | MSFPQS | 1.073 |
| 1053 | **P** | 1051 | 1056 | SFPQSA | 1.095 |
| 1054 | **Q** | 1052 | 1057 | FPQSAP | 1.264 |
| 1055 | **S** | 1053 | 1058 | PQSAPH | 1.986 |
| 1056 | **A** | 1054 | 1059 | QSAPHG | 1.271 |
| 1057 | **P** | 1055 | 1060 | SAPHGV | 0.545 |
| 1058 | **H** | 1056 | 1061 | APHGVV | 0.302 |
| 1059 | **G** | 1057 | 1062 | PHGVVF | 0.259 |
| 1060 | **V** | 1058 | 1063 | HGVVFL | 0.138 |
| 1061 | **V** | 1059 | 1064 | GVVFLH | 0.138 |
| 1062 | **F** | 1060 | 1065 | VVFLHV | 0.103 |
| 1063 | **L** | 1061 | 1066 | VFLHVT | 0.201 |
| 1064 | **H** | 1062 | 1067 | FLHVTY | 0.425 |
| 1065 | **V** | 1063 | 1068 | LHVTYV | 0.364 |
| 1066 | **T** | 1064 | 1069 | HVTYVP | 0.682 |
| 1067 | **Y** | 1065 | 1070 | VTYVPA | 0.507 |
| 1068 | **V** | 1066 | 1071 | TYVPAQ | 1.182 |
| 1069 | **P** | 1067 | 1072 | YVPAQE | 1.418 |
| 1070 | **A** | 1068 | 1073 | VPAQEK | 1.81 |
| 1071 | **Q** | 1069 | 1074 | PAQEKN | 3.922 |
| 1072 | **E** | 1070 | 1075 | AQEKNF | 2.196 |
| 1073 | **K** | 1071 | 1076 | QEKNFT | 3.138 |
| 1074 | **N** | 1072 | 1077 | EKNFTT | 2.615 |
| 1075 | **F** | 1073 | 1078 | KNFTTA | 1.525 |
| 1076 | **T** | 1074 | 1079 | NFTTAP | 1.179 |
| 1077 | **T** | 1075 | 1080 | FTTAPA | 0.741 |
| 1078 | **A** | 1076 | 1081 | TTAPAI | 0.6 |
| 1079 | **P** | 1077 | 1082 | TAPAIC | 0.223 |
| 1080 | **A** | 1078 | 1083 | APAICH | 0.21 |
| 1081 | **I** | 1079 | 1084 | PAICHD | 0.347 |
| 1082 | **C** | 1080 | 1085 | AICHDG | 0.222 |
| 1083 | **H** | 1081 | 1086 | ICHDGK | 0.44 |
| 1084 | **D** | 1082 | 1087 | CHDGKA | 0.634 |
| 1085 | **G** | 1083 | 1088 | HDGKAH | 1.609 |
| 1086 | **K** | 1084 | 1089 | DGKAHF | 1.024 |
| 1087 | **A** | 1085 | 1090 | GKAHFP | 0.948 |
| 1088 | **H** | 1086 | 1091 | KAHFPR | 1.877 |
| 1089 | **F** | 1087 | 1092 | AHFPRE | 1.625 |
| 1090 | **P** | 1088 | 1093 | HFPREG | 1.592 |
| 1091 | **R** | 1089 | 1094 | FPREGV | 0.868 |
| 1092 | **E** | 1090 | 1095 | PREGVF | 0.868 |
| 1093 | **G** | 1091 | 1096 | REGVFV | 0.417 |
| 1094 | **V** | 1092 | 1097 | EGVFVS | 0.285 |
| 1095 | **F** | 1093 | 1098 | GVFVSN | 0.265 |
| 1096 | **V** | 1094 | 1099 | VFVSNG | 0.265 |
| 1097 | **S** | 1095 | 1100 | FVSNGT | 0.515 |
| 1098 | **N** | 1096 | 1101 | VSNGTH | 0.809 |
| 1099 | **G** | 1097 | 1102 | SNGTHW | 1.146 |
| 1100 | **T** | 1098 | 1103 | NGTHWF | 0.741 |
| 1101 | **H** | 1099 | 1104 | GTHWFV | 0.342 |
| 1102 | **W** | 1100 | 1105 | THWFVT | 0.499 |
| 1103 | **F** | 1101 | 1106 | HWFVTQ | 0.598 |
| 1104 | **V** | 1102 | 1107 | WFVTQR | 0.861 |
| 1105 | **T** | 1103 | 1108 | FVTQRN | 1.317 |
| 1106 | **Q** | 1104 | 1109 | VTQRNF | 1.317 |
| 1107 | **R** | 1105 | 1110 | TQRNFY | 2.78 |
| 1108 | **N** | 1106 | 1111 | QRNFYE | 3.336 |
| 1109 | **F** | 1107 | 1112 | RNFYEP | 2.979 |
| 1110 | **Y** | 1108 | 1113 | NFYEPQ | 2.634 |
| 1111 | **E** | 1109 | 1114 | FYEPQI | 1.148 |
| 1112 | **P** | 1110 | 1115 | YEPQII | 0.929 |
| 1113 | **Q** | 1111 | 1116 | EPQIIT | 0.856 |
| 1114 | **I** | 1112 | 1117 | PQIITT | 0.713 |
| 1115 | **I** | 1113 | 1118 | QIITTD | 0.77 |
| 1116 | **T** | 1114 | 1119 | IITTDN | 0.715 |
| 1117 | **T** | 1115 | 1120 | ITTDNT | 1.473 |
| 1118 | **D** | 1116 | 1121 | TTDNTF | 1.819 |
| 1119 | **N** | 1117 | 1122 | TDNTFV | 0.936 |
| 1120 | **T** | 1118 | 1123 | DNTFVS | 0.869 |
| 1121 | **F** | 1119 | 1124 | NTFVSG | 0.515 |
| 1122 | **V** | 1120 | 1125 | TFVSGN | 0.515 |
| 1123 | **S** | 1121 | 1126 | FVSGNC | 0.191 |
| 1124 | **G** | 1122 | 1127 | VSGNCD | 0.369 |
| 1125 | **N** | 1123 | 1128 | SGNCDV | 0.369 |
| 1126 | **C** | 1124 | 1129 | GNCDVV | 0.204 |
| 1127 | **D** | 1125 | 1130 | NCDVVI | 0.145 |
| 1128 | **V** | 1126 | 1131 | CDVVIG | 0.089 |
| 1129 | **V** | 1127 | 1132 | DVVIGI | 0.116 |
| 1130 | **I** | 1128 | 1133 | VVIGIV | 0.052 |
| 1131 | **G** | 1129 | 1134 | VIGIVN | 0.112 |
| 1132 | **I** | 1130 | 1135 | IGIVNN | 0.243 |
| 1133 | **V** | 1131 | 1136 | GIVNNT | 0.5 |
| 1134 | **N** | 1132 | 1137 | IVNNTV | 0.375 |
| 1135 | **N** | 1133 | 1138 | VNNTVY | 0.839 |
| 1136 | **T** | 1134 | 1139 | NNTVYD | 1.887 |
| 1137 | **V** | 1135 | 1140 | NTVYDP | 1.814 |
| 1138 | **Y** | 1136 | 1141 | TVYDPL | 0.93 |
| 1139 | **D** | 1137 | 1142 | VYDPLQ | 1.116 |
| 1140 | **P** | 1138 | 1143 | YDPLQP | 2.326 |
| 1141 | **L** | 1139 | 1144 | DPLQPE | 2.571 |
| 1142 | **Q** | 1140 | 1145 | PLQPEL | 1.269 |
| 1143 | **P** | 1141 | 1146 | LQPELD | 1.371 |
| 1144 | **E** | 1142 | 1147 | QPELDS | 2.228 |
| 1145 | **L** | 1143 | 1148 | PELDSF | 1.114 |
| 1146 | **D** | 1144 | 1149 | ELDSFK | 1.441 |
| 1147 | **S** | 1145 | 1150 | LDSFKE | 1.441 |
| 1148 | **F** | 1146 | 1151 | DSFKEE | 3.026 |
| 1149 | **K** | 1147 | 1152 | SFKEEL | 1.494 |
| 1150 | **E** | 1148 | 1153 | FKEELD | 1.862 |
| 1151 | **E** | 1149 | 1154 | KEELDK | 4.3 |
| 1152 | **L** | 1150 | 1155 | EELDKY | 3.369 |
| 1153 | **D** | 1151 | 1156 | ELDKYF | 1.685 |
| 1154 | **K** | 1152 | 1157 | LDKYFK | 1.945 |
| 1155 | **Y** | 1153 | 1158 | DKYFKN | 3.793 |
| 1156 | **F** | 1154 | 1159 | KYFKNH | 3.091 |
| 1157 | **K** | 1155 | 1160 | YFKNHT | 2.23 |
| 1158 | **N** | 1156 | 1161 | FKNHTS | 1.908 |
| 1159 | **H** | 1157 | 1162 | KNHTSP | 3.406 |
| 1160 | **T** | 1158 | 1163 | NHTSPD | 2.845 |
| 1161 | **S** | 1159 | 1164 | HTSPDV | 1.313 |
| 1162 | **P** | 1160 | 1165 | TSPDVD | 1.611 |
| 1163 | **D** | 1161 | 1166 | SPDVDL | 0.921 |
| 1164 | **V** | 1162 | 1167 | PDVDLG | 0.68 |
| 1165 | **D** | 1163 | 1168 | DVDLGD | 0.734 |
| 1166 | **L** | 1164 | 1169 | VDLGDI | 0.308 |
| 1167 | **G** | 1165 | 1170 | DLGDIS | 0.557 |
| 1168 | **D** | 1166 | 1171 | LGDISG | 0.33 |
| 1169 | **I** | 1167 | 1172 | GDISGI | 0.28 |
| 1170 | **S** | 1168 | 1173 | DISGIN | 0.456 |
| 1171 | **G** | 1169 | 1174 | ISGINA | 0.276 |
| 1172 | **I** | 1170 | 1175 | SGINAS | 0.527 |
| 1173 | **N** | 1171 | 1176 | GINASV | 0.292 |
| 1174 | **A** | 1172 | 1177 | INASVV | 0.219 |
| 1175 | **S** | 1173 | 1178 | NASVVN | 0.502 |
| 1176 | **V** | 1174 | 1179 | ASVVNI | 0.219 |
| 1177 | **V** | 1175 | 1180 | SVVNIQ | 0.375 |
| 1178 | **N** | 1176 | 1181 | VVNIQK | 0.56 |
| 1179 | **I** | 1177 | 1182 | VNIQKE | 1.306 |
| 1180 | **Q** | 1178 | 1183 | NIQKEI | 1.234 |
| 1181 | **K** | 1179 | 1184 | IQKEID | 1.281 |
| 1182 | **E** | 1180 | 1185 | QKEIDR | 3.58 |
| 1183 | **I** | 1181 | 1186 | KEIDRL | 1.705 |
| 1184 | **D** | 1182 | 1187 | EIDRLN | 1.371 |
| 1185 | **R** | 1183 | 1188 | IDRLNE | 1.371 |
| 1186 | **L** | 1184 | 1189 | DRLNEV | 1.451 |
| 1187 | **N** | 1185 | 1190 | RLNEVA | 0.878 |
| 1188 | **E** | 1186 | 1191 | LNEVAK | 0.896 |
| 1189 | **V** | 1187 | 1192 | NEVAKN | 1.748 |
| 1190 | **A** | 1188 | 1193 | EVAKNL | 0.896 |
| 1191 | **K** | 1189 | 1194 | VAKNLN | 0.832 |
| 1192 | **N** | 1190 | 1195 | AKNLNE | 1.942 |
| 1193 | **L** | 1191 | 1196 | KNLNES | 2.577 |
| 1194 | **N** | 1192 | 1197 | NLNESL | 1.062 |
| 1195 | **E** | 1193 | 1198 | LNESLI | 0.463 |
| 1196 | **S** | 1194 | 1199 | NESLID | 0.938 |
| 1197 | **L** | 1195 | 1200 | ESLIDL | 0.481 |
| 1198 | **I** | 1196 | 1201 | SLIDLQ | 0.481 |
| 1199 | **D** | 1197 | 1202 | LIDLQE | 0.622 |
| 1200 | **L** | 1198 | 1203 | IDLQEL | 0.622 |
| 1201 | **Q** | 1199 | 1204 | DLQELG | 0.877 |
| 1202 | **E** | 1200 | 1205 | LQELGK | 1.051 |
| 1203 | **L** | 1201 | 1206 | QELGKY | 1.996 |
| 1204 | **G** | 1202 | 1207 | ELGKYE | 1.996 |
| 1205 | **K** | 1203 | 1208 | LGKYEQ | 1.996 |
| 1206 | **Y** | 1204 | 1209 | GKYEQY | 3.793 |
| 1207 | **E** | 1205 | 1210 | KYEQYI | 2.687 |
| 1208 | **Q** | 1206 | 1211 | YEQYIK | 2.687 |
| 1209 | **Y** | 1207 | 1212 | EQYIKW | 1.803 |
| 1210 | **I** | 1208 | 1213 | QYIKWP | 1.61 |
| 1211 | **K** | 1209 | 1214 | YIKWPW | 0.977 |
| 1212 | **W** | 1210 | 1215 | IKWPWY | 0.977 |
| 1213 | **P** | 1211 | 1216 | KWPWYI | 0.977 |
| 1214 | **W** | 1212 | 1217 | WPWYIW | 0.514 |
| 1215 | **Y** | 1213 | 1218 | PWYIWL | 0.403 |
| 1216 | **I** | 1214 | 1219 | WYIWLG | 0.258 |
| 1217 | **W** | 1215 | 1220 | YIWLGF | 0.212 |
| 1218 | **L** | 1216 | 1221 | IWLGFI | 0.095 |
| 1219 | **G** | 1217 | 1222 | WLGFIA | 0.137 |
| 1220 | **F** | 1218 | 1223 | LGFIAG | 0.129 |
| 1221 | **I** | 1219 | 1224 | GFIAGL | 0.129 |
| 1222 | **A** | 1220 | 1225 | FIAGLI | 0.091 |
| 1223 | **G** | 1221 | 1226 | IAGLIA | 0.107 |
| 1224 | **L** | 1222 | 1227 | AGLIAI | 0.107 |
| 1225 | **I** | 1223 | 1228 | GLIAIV | 0.078 |
| 1226 | **A** | 1224 | 1229 | LIAIVM | 0.078 |
| 1227 | **I** | 1225 | 1230 | IAIVMV | 0.07 |
| 1228 | **V** | 1226 | 1231 | AIVMVT | 0.145 |
| 1229 | **M** | 1227 | 1232 | IVMVTI | 0.101 |
| 1230 | **V** | 1228 | 1233 | VMVTIM | 0.142 |
| 1231 | **T** | 1229 | 1234 | MVTIML | 0.158 |
| 1232 | **I** | 1230 | 1235 | VTIMLC | 0.086 |
| 1233 | **M** | 1231 | 1236 | TIMLCC | 0.062 |
| 1234 | **L** | 1232 | 1237 | IMLCCM | 0.042 |
| 1235 | **C** | 1233 | 1238 | MLCCMT | 0.087 |
| 1236 | **C** | 1234 | 1239 | LCCMTS | 0.118 |
| 1237 | **M** | 1235 | 1240 | CCMTSC | 0.077 |
| 1238 | **T** | 1236 | 1241 | CMTSCC | 0.077 |
| 1239 | **S** | 1237 | 1242 | MTSCCS | 0.192 |
| 1240 | **C** | 1238 | 1243 | TSCCSC | 0.104 |
| 1241 | **C** | 1239 | 1244 | SCCSCL | 0.059 |
| 1242 | **S** | 1240 | 1245 | CCSCLK | 0.089 |
| 1243 | **C** | 1241 | 1246 | CSCLKG | 0.164 |
| 1244 | **L** | 1242 | 1247 | SCLKGC | 0.164 |
| 1245 | **K** | 1243 | 1248 | CLKGCC | 0.065 |
| 1246 | **G** | 1244 | 1249 | LKGCCS | 0.164 |
| 1247 | **C** | 1245 | 1250 | KGCCSC | 0.106 |
| 1248 | **C** | 1246 | 1251 | GCCSCG | 0.053 |
| 1249 | **S** | 1247 | 1252 | CCSCGS | 0.071 |
| 1250 | **C** | 1248 | 1253 | CSCGSC | 0.071 |
| 1251 | **G** | 1249 | 1254 | SCGSCC | 0.071 |
| 1252 | **S** | 1250 | 1255 | CGSCCK | 0.106 |
| 1253 | **C** | 1251 | 1256 | GSCCKF | 0.172 |
| 1254 | **C** | 1252 | 1257 | SCCKFD | 0.29 |
| 1255 | **K** | 1253 | 1258 | CCKFDE | 0.375 |
| 1256 | **F** | 1254 | 1259 | CKFDED | 1.167 |
| 1257 | **D** | 1255 | 1260 | KFDEDD | 3.636 |
| 1258 | **E** | 1256 | 1261 | FDEDDS | 2.436 |
| 1259 | **D** | 1257 | 1262 | DEDDSE | 4.872 |
| 1260 | **D** | 1258 | 1263 | EDDSEP | 4.512 |
| 1261 | **S** | 1259 | 1264 | DDSEPV | 1.934 |
| 1262 | **E** | 1260 | 1265 | DSEPVL | 0.955 |
| 1263 | **P** | 1261 | 1266 | SEPVLK | 1.143 |
| 1264 | **V** | 1262 | 1267 | EPVLKG | 0.844 |
| 1265 | **L** | 1263 | 1268 | PVLKGV | 0.362 |
| 1266 | **K** | 1264 | 1269 | VLKGVK | 0.468 |
| 1267 | **G** | 1265 | 1270 | LKGVKL | 0.52 |
| 1268 | **V** | 1266 | 1271 | KGVKLH | 0.858 |
| 1269 | **K** | 1267 | 1272 | GVKLHY | 0.672 |
| 1270 | **L** | 1268 | 1273 | VKLHYT | 0.98 |
| E | | | | | |

| 3 | S | 1 | 6 | MYSFVS | 0.787 |
| --- | --- | --- | --- | --- | --- |
| 4 | **F** | 2 | 7 | YSFVSE | 1.377 |
| 5 | **V** | 3 | 8 | SFVSEE | 1.522 |
| 6 | **S** | 4 | 9 | FVSEET | 1.639 |
| 7 | **E** | 5 | 10 | VSEETG | 1.873 |
| 8 | **E** | 6 | 11 | SEETGT | 3.643 |
| 9 | **T** | 7 | 12 | EETGTL | 2.242 |
| 10 | **G** | 8 | 13 | ETGTLI | 0.907 |
| 11 | **T** | 9 | 14 | TGTLIV | 0.389 |
| 12 | **L** | 10 | 15 | GTLIVN | 0.433 |
| 13 | **I** | 11 | 16 | TLIVNS | 0.587 |
| 14 | **V** | 12 | 17 | LIVNSV | 0.302 |
| 15 | **N** | 13 | 18 | IVNSVL | 0.302 |
| 16 | **S** | 14 | 19 | VNSVLL | 0.355 |
| 17 | **V** | 15 | 20 | NSVLLF | 0.414 |
| 18 | **L** | 16 | 21 | SVLLFL | 0.212 |
| 19 | **L** | 17 | 22 | VLLFLA | 0.16 |
| 20 | **F** | 18 | 23 | LLFLAF | 0.187 |
| 21 | **L** | 19 | 24 | LFLAFV | 0.168 |
| 22 | **A** | 20 | 25 | FLAFVV | 0.151 |
| 23 | **F** | 21 | 26 | LAFVVF | 0.151 |
| 24 | **V** | 22 | 27 | AFVVFL | 0.151 |
| 25 | **V** | 23 | 28 | FVVFLL | 0.124 |
| 26 | **F** | 24 | 29 | VVFLLV | 0.106 |
| 27 | **L** | 25 | 30 | VFLLVT | 0.206 |
| 28 | **L** | 26 | 31 | FLLVTL | 0.229 |
| 29 | **V** | 27 | 32 | LLVTLA | 0.267 |
| 30 | **T** | 28 | 33 | LVTLAI | 0.227 |
| 31 | **L** | 29 | 34 | VTLAIL | 0.227 |
| 32 | **A** | 30 | 35 | TLAILT | 0.441 |
| 33 | **I** | 31 | 36 | LAILTA | 0.309 |
| 34 | **L** | 32 | 37 | AILTAL | 0.309 |
| 35 | **T** | 33 | 38 | ILTALR | 0.599 |
| 36 | **A** | 34 | 39 | LTALRL | 0.704 |
| 37 | **L** | 35 | 40 | TALRLC | 0.458 |
| 38 | **R** | 36 | 41 | ALRLCA | 0.32 |
| 39 | **L** | 37 | 42 | LRLCAY | 0.497 |
| 40 | **C** | 38 | 43 | RLCAYC | 0.323 |
| 41 | **A** | 39 | 44 | LCAYCC | 0.088 |
| 42 | **Y** | 40 | 45 | CAYCCN | 0.172 |
| 43 | **C** | 41 | 46 | AYCCNI | 0.225 |
| 44 | **C** | 42 | 47 | YCCNIV | 0.166 |
| 45 | **N** | 43 | 48 | CCNIVN | 0.17 |
| 46 | **I** | 44 | 49 | CNIVNV | 0.235 |
| 47 | **V** | 45 | 50 | NIVNVS | 0.588 |
| 48 | **N** | 46 | 51 | IVNVSL | 0.302 |
| 49 | **V** | 47 | 52 | VNVSLV | 0.32 |
| 50 | **S** | 48 | 53 | NVSLVK | 0.861 |
| 51 | **L** | 49 | 54 | VSLVKP | 0.828 |
| 52 | **V** | 50 | 55 | SLVKPS | 1.495 |
| 53 | **K** | 51 | 56 | LVKPSF | 0.966 |
| 54 | **P** | 52 | 57 | VKPSFY | 1.835 |
| 55 | **S** | 53 | 58 | KPSFYV | 1.835 |
| 56 | **F** | 54 | 59 | PSFYVY | 1.438 |
| 57 | **Y** | 55 | 60 | SFYVYS | 1.246 |
| 58 | **V** | 56 | 61 | FYVYSR | 1.821 |
| 59 | **Y** | 57 | 62 | YVYSRV | 1.561 |
| 60 | **S** | 58 | 63 | VYSRVK | 1.992 |
| 61 | **R** | 59 | 64 | YSRVKN | 4.316 |
| 62 | **V** | 60 | 65 | SRVKNL | 2.272 |
| 63 | **K** | 61 | 66 | RVKNLN | 2.726 |
| 64 | **N** | 62 | 67 | VKNLNS | 1.865 |
| 65 | **L** | 63 | 68 | KNLNSS | 3.368 |
| 66 | **N** | 64 | 69 | NLNSSR | 3.298 |
| 67 | **S** | 65 | 70 | LNSSRV | 1.522 |
| 68 | **S** | 66 | 71 | NSSRVP | 2.854 |
| 69 | **R** | 67 | 72 | SSRVPD | 2.964 |
| 70 | **V** | 68 | 73 | SRVPDL | 1.824 |
| 71 | **P** | 69 | 74 | RVPDLL | 1.123 |
| 72 | **D** | 70 | 75 | VPDLLV | 0.425 |
| M | | | | | |
| 3 | **D** | 1 | 6 | MADSNG | 1.179 |
| 4 | **S** | 2 | 7 | ADSNGT | 1.719 |
| 5 | **N** | 3 | 8 | DSNGTI | 1.193 |
| 6 | **G** | 4 | 9 | SNGTIT | 1.031 |
| 7 | **T** | 5 | 10 | NGTITV | 0.571 |
| 8 | **I** | 6 | 11 | GTITVE | 0.615 |
| 9 | **T** | 7 | 12 | TITVEE | 1.076 |
| 10 | **V** | 8 | 13 | ITVEEL | 0.615 |
| 11 | **E** | 9 | 14 | TVEELK | 1.754 |
| 12 | **E** | 10 | 15 | VEELKK | 2.431 |
| 13 | **L** | 11 | 16 | EELKKL | 2.701 |
| 14 | **K** | 12 | 17 | ELKKLL | 1.286 |
| 15 | **K** | 13 | 18 | LKKLLE | 1.286 |
| 16 | **L** | 14 | 19 | KKLLEQ | 2.701 |
| 17 | **L** | 15 | 20 | KLLEQW | 1.42 |
| 18 | **E** | 16 | 21 | LLEQWN | 1.142 |
| 19 | **Q** | 17 | 22 | LEQWNL | 1.142 |
| 20 | **W** | 18 | 23 | EQWNLV | 1.028 |
| 21 | **N** | 19 | 24 | QWNLVI | 0.416 |
| 22 | **L** | 20 | 25 | WNLVIG | 0.238 |
| 23 | **V** | 21 | 26 | NLVIGF | 0.196 |
| 24 | **I** | 22 | 27 | LVIGFL | 0.1 |
| 25 | **G** | 23 | 28 | VIGFLF | 0.105 |
| 26 | **F** | 24 | 29 | IGFLFL | 0.117 |
| 27 | **L** | 25 | 30 | GFLFLT | 0.241 |
| 28 | **F** | 26 | 31 | FLFLTW | 0.256 |
| 29 | **L** | 27 | 32 | LFLTWI | 0.207 |
| 30 | **T** | 28 | 33 | FLTWIC | 0.135 |
| 31 | **W** | 29 | 34 | LTWICL | 0.128 |
| 32 | **I** | 30 | 35 | TWICLL | 0.128 |
| 33 | **C** | 31 | 36 | WICLLQ | 0.154 |
| 34 | **L** | 32 | 37 | ICLLQF | 0.127 |
| 35 | **L** | 33 | 38 | CLLQFA | 0.183 |
| 36 | **Q** | 34 | 39 | LLQFAY | 0.535 |
| 37 | **F** | 35 | 40 | LQFAYA | 0.655 |
| 38 | **A** | 36 | 41 | QFAYAN | 1.277 |
| 39 | **Y** | 37 | 42 | FAYANR | 1.444 |
| 40 | **A** | 38 | 43 | AYANRN | 2.682 |
| 41 | **N** | 39 | 44 | YANRNR | 5.199 |
| 42 | **R** | 40 | 45 | ANRNRF | 2.873 |
| 43 | **N** | 41 | 46 | NRNRFL | 2.346 |
| 44 | **R** | 42 | 47 | RNRFLY | 2.285 |
| 45 | **F** | 43 | 48 | NRFLYI | 0.818 |
| 46 | **L** | 44 | 49 | RFLYII | 0.357 |
| 47 | **Y** | 45 | 50 | FLYIIK | 0.364 |
| 48 | **I** | 46 | 51 | LYIIKL | 0.347 |
| 49 | **I** | 47 | 52 | YIIKLI | 0.295 |
| 50 | **K** | 48 | 53 | IIKLIF | 0.163 |
| 51 | **L** | 49 | 54 | IKLIFL | 0.192 |
| 52 | **I** | 50 | 55 | KLIFLW | 0.287 |
| 53 | **F** | 51 | 56 | LIFLWL | 0.119 |
| 54 | **L** | 52 | 57 | IFLWLL | 0.119 |
| 55 | **W** | 53 | 58 | FLWLLW | 0.178 |
| 56 | **L** | 54 | 59 | LWLLWP | 0.317 |
| 57 | **L** | 55 | 60 | WLLWPV | 0.286 |
| 58 | **W** | 56 | 61 | LLWPVT | 0.392 |
| 59 | **P** | 57 | 62 | LWPVTL | 0.392 |
| 60 | **V** | 58 | 63 | WPVTLA | 0.48 |
| 61 | **T** | 59 | 64 | PVTLAC | 0.245 |
| 62 | **L** | 60 | 65 | VTLACF | 0.137 |
| 63 | **A** | 61 | 66 | TLACFV | 0.137 |
| 64 | **C** | 62 | 67 | LACFVL | 0.078 |
| 65 | **F** | 63 | 68 | ACFVLA | 0.096 |
| 66 | **V** | 64 | 69 | CFVLAA | 0.096 |
| 67 | **L** | 65 | 70 | FVLAAV | 0.133 |
| 68 | **A** | 66 | 71 | VLAAVY | 0.241 |
| 69 | **A** | 67 | 72 | LAAVYR | 0.635 |
| 70 | **V** | 68 | 73 | AAVYRI | 0.54 |
| 71 | **Y** | 69 | 74 | AVYRIN | 0.859 |
| 72 | **R** | 70 | 75 | VYRINW | 0.894 |
| 73 | **I** | 71 | 76 | YRINWI | 0.844 |
| 74 | **N** | 72 | 77 | RINWIT | 0.778 |
| 75 | **W** | 73 | 78 | INWITG | 0.393 |
| 76 | **I** | 74 | 79 | NWITGG | 0.555 |
| 77 | **T** | 75 | 80 | WITGGI | 0.242 |
| 78 | **G** | 76 | 81 | ITGGIA | 0.232 |
| 79 | **G** | 77 | 82 | TGGIAI | 0.232 |
| 80 | **I** | 78 | 83 | GGIAIA | 0.163 |
| 81 | **A** | 79 | 84 | GIAIAM | 0.163 |
| 82 | **I** | 80 | 85 | IAIAMA | 0.166 |
| 83 | **A** | 81 | 86 | AIAMAC | 0.127 |
| 84 | **M** | 82 | 87 | IAMACL | 0.104 |
| 85 | **A** | 83 | 88 | AMACLV | 0.11 |
| 86 | **C** | 84 | 89 | MACLVG | 0.107 |
| 87 | **L** | 85 | 90 | ACLVGL | 0.09 |
| 88 | **V** | 86 | 91 | CLVGLM | 0.088 |
| 89 | **G** | 87 | 92 | LVGLMW | 0.172 |
| 90 | **L** | 88 | 93 | VGLMWL | 0.172 |
| 91 | **M** | 89 | 94 | GLMWLS | 0.311 |
| 92 | **W** | 90 | 95 | LMWLSY | 0.492 |
| 93 | **L** | 91 | 96 | MWLSYF | 0.517 |
| 94 | **S** | 92 | 97 | WLSYFI | 0.366 |
| 95 | **Y** | 93 | 98 | LSYFIA | 0.352 |
| 96 | **F** | 94 | 99 | SYFIAS | 0.571 |
| 97 | **I** | 95 | 100 | YFIASF | 0.369 |
| 98 | **A** | 96 | 101 | FIASFR | 0.461 |
| 99 | **S** | 97 | 102 | IASFRL | 0.439 |
| 100 | **F** | 98 | 103 | ASFRLF | 0.543 |
| 101 | **R** | 99 | 104 | SFRLFA | 0.543 |
| 102 | **L** | 100 | 105 | FRLFAR | 0.793 |
| 103 | **F** | 101 | 106 | RLFART | 1.322 |
| 104 | **A** | 102 | 107 | LFARTR | 1.322 |
| 105 | **R** | 103 | 108 | FARTRS | 2.149 |
| 106 | **T** | 104 | 109 | ARTRSM | 2.456 |
| 107 | **R** | 105 | 110 | RTRSMW | 2.556 |
| 108 | **S** | 106 | 111 | TRSMWS | 1.749 |
| 109 | **M** | 107 | 112 | RSMWSF | 1.049 |
| 110 | **W** | 108 | 113 | SMWSFN | 0.862 |
| 111 | **S** | 109 | 114 | MWSFNP | 0.994 |
| 112 | **F** | 110 | 115 | WSFNPE | 1.74 |
| 113 | **N** | 111 | 116 | SFNPET | 2.388 |
| 114 | **P** | 112 | 117 | FNPETN | 2.865 |
| 115 | **E** | 113 | 118 | NPETNI | 2.32 |
| 116 | **T** | 114 | 119 | PETNIL | 1.19 |
| 117 | **N** | 115 | 120 | ETNILL | 0.634 |
| 118 | **I** | 116 | 121 | TNILLN | 0.589 |
| 119 | **L** | 117 | 122 | NILLNV | 0.303 |
| 120 | **L** | 118 | 123 | ILLNVP | 0.291 |
| 121 | **N** | 119 | 124 | LLNVPL | 0.343 |
| 122 | **V** | 120 | 125 | LNVPLH | 0.565 |
| 123 | **P** | 121 | 126 | NVPLHG | 0.679 |
| 124 | **L** | 122 | 127 | VPLHGT | 0.609 |
| 125 | **H** | 123 | 128 | PLHGTI | 0.575 |
| 126 | **G** | 124 | 129 | LHGTIL | 0.307 |
| 127 | **T** | 125 | 130 | HGTILT | 0.537 |
| 128 | **I** | 126 | 131 | GTILTR | 0.773 |
| 129 | **L** | 127 | 132 | TILTRP | 1.207 |
| 130 | **T** | 128 | 133 | ILTRPL | 0.69 |
| 131 | **R** | 129 | 134 | LTRPLL | 0.812 |
| 132 | **P** | 130 | 135 | TRPLLE | 1.704 |
| 133 | **L** | 131 | 136 | RPLLES | 1.583 |
| 134 | **L** | 132 | 137 | PLLESE | 1.399 |
| 135 | **E** | 133 | 138 | LLESEL | 0.746 |
| 136 | **S** | 134 | 139 | LESELV | 0.672 |
| 137 | **E** | 135 | 140 | ESELVI | 0.571 |
| 138 | **L** | 136 | 141 | SELVIG | 0.326 |
| 139 | **V** | 137 | 142 | ELVIGA | 0.246 |
| 140 | **I** | 138 | 143 | LVIGAV | 0.105 |
| 141 | **G** | 139 | 144 | VIGAVI | 0.09 |
| 142 | **A** | 140 | 145 | IGAVIL | 0.1 |
| 143 | **V** | 141 | 146 | GAVILR | 0.278 |
| 144 | **I** | 142 | 147 | AVILRG | 0.278 |
| 145 | **L** | 143 | 148 | VILRGH | 0.375 |
| 146 | **R** | 144 | 149 | ILRGHL | 0.416 |
| 147 | **G** | 145 | 150 | LRGHLR | 1.163 |
| 148 | **H** | 146 | 151 | RGHLRI | 0.989 |
| 149 | **L** | 147 | 152 | GHLRIA | 0.51 |
| 150 | **R** | 148 | 153 | HLRIAG | 0.51 |
| 151 | **I** | 149 | 154 | LRIAGH | 0.51 |
| 152 | **A** | 150 | 155 | RIAGHH | 0.841 |
| 153 | **G** | 151 | 156 | IAGHHL | 0.354 |
| 154 | **H** | 152 | 157 | AGHHLG | 0.5 |
| 155 | **H** | 153 | 158 | GHHLGR | 0.97 |
| 156 | **L** | 154 | 159 | HHLGRC | 0.525 |
| 157 | **G** | 155 | 160 | HLGRCD | 0.645 |
| 158 | **R** | 156 | 161 | LGRCDI | 0.332 |
| 159 | **C** | 157 | 162 | GRCDIK | 0.805 |
| 160 | **D** | 158 | 163 | RCDIKD | 1.359 |
| 161 | **I** | 159 | 164 | CDIKDL | 0.572 |
| 162 | **K** | 160 | 165 | DIKDLP | 1.651 |
| 163 | **D** | 161 | 166 | IKDLPK | 1.977 |
| 164 | **L** | 162 | 167 | KDLPKE | 4.883 |
| 165 | **P** | 163 | 168 | DLPKEI | 1.712 |
| 166 | **K** | 164 | 169 | LPKEIT | 1.479 |
| 167 | **E** | 165 | 170 | PKEITV | 1.331 |
| 168 | **I** | 166 | 171 | KEITVA | 0.87 |
| 169 | **T** | 167 | 172 | EITVAT | 0.628 |
| 170 | **V** | 168 | 173 | ITVATS | 0.486 |
| 171 | **A** | 169 | 174 | TVATSR | 1.357 |
| 172 | **T** | 170 | 175 | VATSRT | 1.357 |
| 173 | **S** | 171 | 176 | ATSRTL | 1.508 |
| 174 | **R** | 172 | 177 | TSRTLS | 2.0 |
| 175 | **T** | 173 | 178 | SRTLSY | 2.172 |
| 176 | **L** | 174 | 179 | RTLSYY | 2.539 |
| 177 | **S** | 175 | 180 | TLSYYK | 2.593 |
| 178 | **Y** | 176 | 181 | LSYYKL | 1.482 |
| 179 | **Y** | 177 | 182 | SYYKLG | 1.778 |
| 180 | **K** | 178 | 183 | YYKLGA | 1.34 |
| 181 | **L** | 179 | 184 | YKLGAS | 1.146 |
| 182 | **G** | 180 | 185 | KLGASQ | 1.267 |
| 183 | **A** | 181 | 186 | LGASQR | 1.241 |
| 184 | **S** | 182 | 187 | GASQRV | 1.117 |
| 185 | **Q** | 183 | 188 | ASQRVA | 1.14 |
| 186 | **R** | 184 | 189 | SQRVAG | 1.117 |
| 187 | **V** | 185 | 190 | QRVAGD | 1.392 |
| 188 | **A** | 186 | 191 | RVAGDS | 1.077 |
| 189 | **G** | 187 | 192 | VAGDSG | 0.544 |
| 190 | **D** | 188 | 193 | AGDSGF | 0.635 |
| 191 | **S** | 189 | 194 | GDSGFA | 0.635 |
| 192 | **G** | 190 | 195 | DSGFAA | 0.648 |
| 193 | **F** | 191 | 196 | SGFAAY | 0.608 |
| 194 | **A** | 192 | 197 | GFAAYS | 0.608 |
| 195 | **A** | 193 | 198 | FAAYSR | 1.203 |
| 196 | **Y** | 194 | 199 | AAYSRY | 2.177 |
| 197 | **S** | 195 | 200 | AYSRYR | 4.222 |
| 198 | **R** | 196 | 201 | YSRYRI | 2.929 |
| 199 | **Y** | 197 | 202 | SRYRIG | 1.85 |
| 200 | **R** | 198 | 203 | RYRIGN | 2.22 |
| 201 | **I** | 199 | 204 | YRIGNY | 1.776 |
| 202 | **G** | 200 | 205 | RIGNYK | 2.267 |
| 203 | **N** | 201 | 206 | IGNYKL | 0.954 |
| 204 | **Y** | 202 | 207 | GNYKLN | 2.19 |
| 205 | **K** | 203 | 208 | NYKLNT | 3.193 |
| 206 | **L** | 204 | 209 | YKLNTD | 3.316 |
| 207 | **N** | 205 | 210 | KLNTDH | 2.88 |
| 208 | **T** | 206 | 211 | LNTDHS | 1.93 |
| 209 | **D** | 207 | 212 | NTDHSS | 3.136 |
| 210 | **H** | 208 | 213 | TDHSSS | 2.613 |
| 211 | **S** | 209 | 214 | DHSSSS | 2.427 |
| 212 | **S** | 210 | 215 | HSSSSD | 2.427 |
| 213 | **S** | 211 | 216 | SSSSDN | 2.868 |
| 214 | **S** | 212 | 217 | SSSDNI | 1.5 |
| 215 | **D** | 213 | 218 | SSDNIA | 1.131 |
| 216 | **N** | 214 | 219 | SDNIAL | 0.696 |
| 217 | **I** | 215 | 220 | DNIALL | 0.428 |
| 218 | **A** | 216 | 221 | NIALLV | 0.19 |
| 219 | **L** | 217 | 222 | IALLVQ | 0.205 |
