# Supplementary material for: Reverse vaccinology assisted designing of multiepitope-based subunit vaccine against SARS-CoV-2
Source: Infect Dis Poverty. 2020 Sep 16;9:132. doi: 10.1186/s40249-020-00752-w (PMC7492789; doi:10.1186/s40249-020-00752-w)
Supplement: Supplementary file 5 — Additional file 5: Table S4. Discontinuous epitopes predicted through DiscoTop 2.0 server. [file 40249_2020_752_MOESM5_ESM.docx]

Table S4. Discontinuous epitopes predicted through DiscoTop 2.0 server

| Protein | Residues position | Residues names | Number of contacts | Propensity score | DicsoTope score |  |
| --- | --- | --- | --- | --- | --- | --- |
| S | 282 | ASN | 4 | -2.346 | -2.537 | |
|  | 415 | THR | 1 | -3.642 | -3.338 |  |
|  | 439 | ASN | 14 | -2.039 | -3.415 |  |
|  | 440 | ASN | 4 | -1.934 | -2.172 |  |
|  | 448 | ASN | 23 | -0.603 | -3.178 |  |
|  | 449 | TYR | 4 | -0.399 | -0.813 |  |
|  | 450 | ASN | 8 | -1.453 | -2.206 |  |
|  | 455 | LEU | 6 | -1.119 | -1.680 |  |
|  | 456 | PHE | 5 | -0.760 | -1.247 |  |
|  | 457 | ARG | 22 | 0.500 | -2.087 |  |
|  | 458 | LYS | 4 | 1.236 | 0.634 |  |
|  | 459 | SER | 4 | 1.271 | 0.665 |  |
|  | 460 | ASN | 13 | 0.073 | -1.430 |  |
|  | 462 | LYS | 1 | -3.956 | -3.616 |  |
|  | 468 | ILE | 2 | -2.185 | -2.164 |  |
|  | 469 | SER | 13 | -1.347 | -2.687 |  |
|  | 470 | THR | 4 | -2.130 | -2.345 |  |
|  | 490 | PHE | 4 | -2.056 | -2.279 |  |
|  | 492 | LEU | 18 | -1.230 | -3.158 |  |
|  | 493 | GLN | 12 | -0.625 | -1.933 |  |
|  | 494 | SER | 8 | -1.035 | -1.836 |  |
|  | 496 | GLY | 2 | 0.335 | 0.067 |  |
|  | 498 | GLN | 3 | 1.807 | 1.254 |  |
|  | 499 | PRO | 4 | 1.917 | 1.237 |  |
|  | 500 | THR | 2 | 3.437 | 2.811 |  |
|  | 501 | ASN | 22 | 2.379 | -0.425 |  |
|  | 503 | VAL | 5 | -0.472 | -0.993 |  |
|  | 504 | GLY | 4 | -2.462 | -2.638 |  |
|  | 505 | TYR | 10 | -0.741 | -1.806 |  |
|  | 556 | ASN | 0 | -3.687 | -3.263 |  |
|  | 558 | LYS | 0 | -1.282 | -1.135 |  |
|  | 560 | LEU | 4 | -3.379 | -3.450 |  |
|  | 561 | PRO | 0 | -3.821 | -3.382 |  |
|  | 703 | ASN | 3 | -2.182 | -2.276 |  |
|  | 704 | SER | 3 | -1.361 | -1.549 |  |
|  | 705 | VAL | 9 | -2.785 | -3.499 |  |
|  | 793 | PRO | 0 | -1.814 | -1.605 |  |
|  | 794 | ILE | 4 | -2.148 | -2.361 |  |
|  | 809 | PRO | 6 | -2.403 | -2.816 |  |
|  | 810 | SER | 4 | 0.639 | 0.106 |  |
|  | 811 | LYS | 18 | -0.867 | -2.837 |  |
|  | 914 | ASN | 7 | -0.804 | -1.516 |  |
|  | 917 | TYR | 9 | -2.580 | -3.318 |  |
|  | 918 | GLU | 12 | -2.383 | -3.489 |  |
|  | 1071 | GLN | 4 | -3.248 | -3.334 |  |
|  | 1100 | THR | 0 | -3.478 | -3.078 |  |
|  | 1118 | ASP | 7 | -2.987 | -3.449 |  |
|  | 1140 | PRO | 8 | -0.757 | -1.590 |  |
|  | 1141 | LEU | 3 | -0.698 | -0.963 |  |
|  | 1142 | GLN | 6 | -0.050 | -0.735 |  |
|  | 1143 | PRO | 6 | 0.444 | -0.297 |  |
|  | 1144 | GLU | 4 | 0.587 | 0.060 |  |
|  | 1145 | LEU | 5 | -0.213 | -0.763 |  |
|  | 1146 | ASP | 5 | 0.694 | 0.039 |  |
|  | 1147 | SER | 6 | 0.000 | -0.690 |  |
| E | 63 | LYS | 4 | -3.653 | -3.693 |  |
| M | 1 | MET | 9 | -0.899 | -1.831 |  |
|  | 2 | ALA | 6 | -2.467 | -2.873 |  |
|  | 3 | ASP | 10 | -2.746 | -3.580 |  |
|  | 4 | SER | 0 | -2.407 | -2.131 |  |
|  | 202 | GLY | 2 | -3.269 | -3.123 |  |
|  | 203 | ASN | 2 | -2.220 | -2.195 |  |
|  | 204 | TYR | 17 | -1.043 | -2.878 |  |
|  | 205 | LYS | 5 | 0.198 | -0.400 |  |
|  | 206 | LEU | 4 | -0.429 | -0.840 |  |
|  | 207 | ASN | 9 | 0.348 | -0.727 |  |
|  | 208 | THR | 6 | 1.343 | 0.498 |  |
|  | 209 | ASP | 0 | 1.026 | 0.908 |  |
|  | 210 | HIS | 3 | 0.399 | 0.008 |  |
|  | 211 | SER | 3 | 0.359 | -0.027 |  |
|  | 212 | SER | 6 | -0.919 | -1.503 |  |
|  | 213 | SER | 2 | -0.541 | -0.709 |  |
|  | 214 | SER | 3 | -0.862 | -1.108 |  |
|  | 215 | ASP | 0 | -1.638 | -1.449 |  |
|  | 216 | ASN | 3 | -2.148 | -2.246 |  |
|  | 217 | ILE | 3 | -2.798 | -2.821 |  |
|  | 218 | ALA | 3 | -3.375 | -3.332 |  |
|  | 222 | GLN | 3 | -3.606 | -3.537 |  |
