# Supplementary material for: Reverse vaccinology assisted designing of multiepitope-based subunit vaccine against SARS-CoV-2
Source: Infect Dis Poverty. 2020 Sep 16;9:132. doi: 10.1186/s40249-020-00752-w (PMC7492789; doi:10.1186/s40249-020-00752-w)
Supplement: Supplementary file 6 — Additional file 6: Table S5. MHC class-I allele and MHC class-II binding peptides with their antigenicity scores. [file 40249_2020_752_MOESM6_ESM.docx]

Table S5. MHC class-I allele and MHC class-II binding peptides with their antigenicity scores

| Protein | Peptides (position) | Alleles | Antigenicity |
| --- | --- | --- | --- |
| MHC class I | | | |
| S | VRFPNITNLCPF (327-338) | HLA-B*35:03, HLA-B*53:01, HLA-A*24:02, HLA-C*07:01,, HLA-B*35:01, HLA-C*06:02, HLA-A*23:01, HLA-B*51:01, HLA-C*14:02, | 1.2 |
|  | ALQIPFAMQMAY (893-904) | HLA-B*35:01, HLA-B*15:01, HLA-A*29:02, HLA-A*03:01, HLA-A*30:02, HLA-B*18:01, HLA-A*25:01 | 0.9 |
|  | IPFAMQMAYRFN (896-907) | HLA-B*35:01, HLA-B*35:03, HLA-A*24:02, HLA-B*51:01, HLA-B*53:01 | 1.3 |
| E | PSFYVYSRVKNL (54-65) | HLA-C*14:02, HLA-C*07:01, HLA-C*06:02 | 0.8 |
|  | SFYVYSRVKNLN (55-66) | HLA-C*14:02, HLA-C*07:01, HLA-C*06:02 | 0.9 |
|  | FYVYSRVKNLNS (56-67) | HLA-C*14:02, HLA-C*07:01, HLA-C*06:02 | 0.6 |
| M | YRINWITGGIAI (71-82) | HLA-B*27:05, HLA-A*32:01, HLA-C*06:02, HLA-B*39:01, HLA-C*07:01 | 1.2 |
|  | SFRLFARTRSMW (99-110) | HLA-C*06:02, HLA-C*07:01, HLA-B*14:02, HLA-A*32:01, HLA-B*57:01 | 0.6 |
|  | ITVATSRTLSYY (168-179) | HLA-A*01:01, HLA-A*30:02, HLA-A*26:01, HLA-A*29:02, HLA-B*57:01 | 0.7 |
| MHC class II | | | |
| S | FVFLVLLPLVSSQCV (2-16) | HLA-DRB1*13:21, HLA-DRB1*01:01, HLA-DRB1*15:02, HLA-DRB1*11:28, HLA-DRB1*13:05, HLA-DPA1*03:01/DPB1*04:02, HLA-DRB1*13:07, HLA-DRB1*11:01, HLA-DRB1*11:02, HLA-DRB1*11:21, HLA-DRB1*13:22, HLA-DRB1*11:04, HLA-DRB1*11:06, HLA-DRB1*13:11, HLA-DRB1*08:17, HLA-DRB1*13:01, HLA-DRB1*13:27, HLA-DRB1*13:28, HLA-DRB1*11:14, HLA-DRB1*13:23, HLA-DRB1*07:03, HLA-DRB1*04:08 | 0.7 |
| E | LLFLAFVVFLLVTLA (18-32) | HLA-DPA1*03:01/DPB1*04:02, HLA-DPA1*01:03/DPB1*02:01, HLA-DPA1*01/DPB1*04:01, HLA-DPA1*02:01/DPB1*01:01, HLA-DRB1*15:02, HLA-DRB1*04:23, HLA-DRB1*04:04, HLA-DRB1*04:08, HLA-DRB1*04:10, HLA-DQA1*05:01/DQB1*02:01, HLA-DRB1*08:13, HLA-DRB1*07:03, HLA-DRB1*01:02, HLA-DRB1*04:05 | 0.8 |
|  | AFVVFLLVTLAILTA (22-36) | HLA-DPA1*03:01/DPB1*04:02, HLA-DRB1*15:02, HLA-DRB1*04:23, HLA-DRB1*11:04, HLA-DRB1*11:06, HLA-DRB1*13:11, HLA-DRB1*04:08, HLA-DRB1*04:10, HLA-DRB1*11:28, HLA-DRB1*13:05, HLA-DPA1*01/DPB1*04:01, HLA-DRB1*04:21, HLA-DRB1*08:13, HLA-DRB1*04:01, HLA-DRB1*04:26, HLA-DRB1*07:03, HLA-DRB1*01:01, HLA-DPA1*02:01/DPB1*01:01, HLA-DRB1*01:02, HLA-DRB1*04:05, HLA-DRB1*13:07, | 0.6 |
|  | FVVFLLVTLAILTAL | HLA-DPA1*03:01/DPB1*04:02, HLA-DRB1*15:02, HLA-DRB1*04:23, HLA-DRB1*11:04, HLA-DRB1*11:06, DRB1*13:11, HLA-DRB1*04:08, HLA-DRB1*04:10, HLA-DRB1*11:28, HLA-DRB1*13:05, HLA-DPA1*01/DPB1*04:01, HLA-DRB1*01:01, HLA-DRB1*04:01, HLA-DRB1*04:26, HLA-DRB1*07:03, HLA-DPA1*02:01/DPB1*01:01, HLA-DRB1*01:02, HLA-DRB1*04:05, HLA-DRB1*13:07 | 0.5 |
| M | ASFRLFARTRSMWSF (98-112) | HLA-DRB1*08:13, HLA-DRB1*11:14, HLA-DRB1*13:23, HLA-DRB1*15:02, HLA-DRB1*11:20, HLA-DRB1*11:01 HLA-DRB1*13:07, HLA-DRB1*15:06 HLA-DRB1*11:28, HLA-DRB1*13:05 HLA-DRB1*04:01, HLA-DRB1*04:26 HLA-DRB1*07:01, HLA-DRB1*11:02 HLA-DRB1*11:21, HLA-DRB1*13:22 HLA-DRB1*03:05, | 0.7 |
|  | FRLFARTRSMWSFNP (100-114) | HLA-DRB1*08:13, HLA-DRB1*11:14, HLA-DRB1*13:23, HLA-DRB1*15:02, HLA-DRB1*11:20, HLA-DRB1*13:07, HLA-DRB1*11:01, HLA-DRB1*15:06, HLA-DRB1*11:28, HLA-DRB1*04:01, HLA-DRB1*04:26, HLA-DRB1*11:02, HLA-DRB1*11:21, HLA-DRB1*13:22, HLA-DRB1*03:05 | 0.8 |
|  | VTLACFVLAAVYRIN (60-74) | HLA-DRB1*07:03, HLA-DRB1*11:20, HLA-DRB1*01:02, HLA-DRB1*07:01, HLA-DRB1*11:14, HLA-DRB1*13:23, HLA-DRB1*03:09, HLA-DRB1*13:07, HLA-DRB1*11:28, HLA-DRB1*13:05, HLA-DRB1*03:05, HLA-DRB1*04:08 | 1.0 |
