# Supplementary material for: Reverse vaccinology assisted designing of multiepitope-based subunit vaccine against SARS-CoV-2
Source: Infect Dis Poverty. 2020 Sep 16;9:132. doi: 10.1186/s40249-020-00752-w (PMC7492789; doi:10.1186/s40249-020-00752-w)
Supplement: Supplementary file 7 — Additional file 7: Table S6. Digestion, allergenicity, toxicity and physiochemical profiling of selected peptides (NA: not allergic; NT: nontoxic). [file 40249_2020_752_MOESM7_ESM.docx]

Table S6. Digestion, allergenicity, toxicity and physiochemical profiling of selected peptides (NA: not allergic; NT: nontoxic)

| Peptides | Non-digesting enzyme | Allergenicity | Toxicity | Hydrophilicity | Hydrophobicity | | Charge | | | pI | M.W |
| --- | --- | --- | --- | --- | --- | --- | --- | --- | --- | --- | --- |
| MHC class I | | | | | | | | | | | |
| VRFPNITNLCPF | Chymotrypsin,Cyanogen_Bromide, IodosoBenzoate,Staph_Protease, Trypsin_K, AspN, | NA | NT | -0.67 | -0.02 | 1.00 | | 8.60 | | | 1420.86 |
| ALQIPFAMQMAY | Trypsin, Clostripain, IodosoBenzoate, Staph_Protease, Trypsin_K, Trypsin_R, AspN, | NA | NT | -1.01 | 0.14 | 0.00 | | 5.88 | | | 1383.86 |
| IPFAMQMAYRFN | IodosoBenzoate, Staph_Protease, Trypsin_K, AspN | NA | NT | -0.78 | -0.01 | 1.00 | | 9.10 | | | 1488.95 |
| PSFYVYSRVKNL | Cyanogen_Bromide, IodosoBenzoate, Staph_Protease, AspN | NA | NT | -0.42 | -0.15 | 2.00 | | 9.72 | | | 1472.87 |
| SFYVYSRVKNLN | AspN, Staph_Protease, Proline_Endopept, IodosoBenzoate, Cyanogen_Bromide | NA | NT | -0.41 | -0.20 | 2.00 | | 9.72 | | | 1489.86 |
| FYVYSRVKNLNS | Cyanogen_Bromide, IodosoBenzoate, Proline_Endopept, Staph_Protease, AspN | NA | NT | -0.41 | -0.20 | 2.00 | | 9.70 | | | 1489.86 |
| YRINWITGGIAI | Cyanogen_Bromide, Proline_Endopept, Staph_Protease, Trypsin_K, AspN | NA | NT | -0.88 | 0.11 | 1.00 | | 9.10 | | | 1376.81 |
| SFRLFARTRSMW | IodosoBenzoate, Proline_Endopept, Staph_Protease, Trypsin_K, AspN | NA | NT | -0.23 | -0.28 | 3.00 | | 12.31 | | | 1557.99 |
| ITVATSRTLSYY | Cyanogen_Bromide, IodosoBenzoate, Proline_Endopept, Staph_Protease, Trypsin_K, AspN | NA | NT | -0.65 | -0.06 | 1.00 | | 8.93 | | | 1374.72 |
| MHC class II | | | | | | | | | | | |
| FVFLVLLPLVSSQCV | Trypsin, Clostripain, Cyanogen_Bromide, IodosoBenzoate, Staph_Protease, Trypsin_K, Trypsin_R, AspN | NA | NT | -1.23 | 0.28 | | 0.00 | | 5.85 | | 1664.31 |
| LLFLAFVVFLLVTLA | Trypsin, Clostripain, Cyanogen_Bromide, IodosoBenzoate, Proline_Endopept, Staph_Protease, Trypsin_K, Trypsin_R,  AspN | NA | NT | -1.61 | 0.46 | | 0.00 | | 5.88 | | 1679.40 |
| AFVVFLLVTLAILTA | AspN, Trypsin_R, Trypsin_K, Staph_Protease, Proline_Endopept,  IodosoBenzoate, Cyanogen_Bromide, Clostripain, Trypsin | NA | NT | -1.39 | 0.41 | | 0.00 | | 5.88 | | 1591.24 |
| FVVFLLVTLAILTAL | Trypsin,  Clostripain, Cyanogen_Bromide, IodosoBenzoate, Proline_Endopept, Staph_Protease, Trypsin_K, Trypsin_R, AspN | NA | NT | -1.47 | 0.42 | | 0.00 | | 5.88 | | 1633.33 |
| ASFRLFARTRSMWSF | Proline_Endopept, Staph_Protease, Trypsin_K, AspN | NA | NT | -0.18 | -0.37 | | 3.00 | | 12.31 | | 1863.36 |
| FRLFARTRSMWSFNP | Proline_Endopept, Staph_Protease, Trypsin_K, AspN | NA | NT | -0.34 | -0.23 | | 3.00 | | 12.31 | | 1916.43 |
| VTLACFVLAAVYRIN | AspN, Trypsin_K, Staph_Protease, Proline_Endopept, IodosoBenzoate, Cyanogen_Bromide | NA | NT | -0.96 | 0.15 | | 1.00 | | 8.57 | | 1653.23 |
