# Supplementary material for: Reverse vaccinology assisted designing of multiepitope-based subunit vaccine against SARS-CoV-2
Source: Infect Dis Poverty. 2020 Sep 16;9:132. doi: 10.1186/s40249-020-00752-w (PMC7492789; doi:10.1186/s40249-020-00752-w)
Supplement: Supplementary file 8 — Additional file 8: Table S7. Linear B cell epitopes predicted in vaccine construct. [file 40249_2020_752_MOESM8_ESM.docx]

Table S7. Linear B cell epitopes predicted in vaccine construct

| Sr.No | Linear B cell epitope | Position | Score | Antigenicity |
| --- | --- | --- | --- | --- |
|  | GIAIAAYSFRLFAR | 74 | 0.88 | 0.6 |
|  | FRLFARTRSMWSFN | 198 | 0.87 | 0.7 |
|  | CCRRKKEAAAKVRF | 40 | 0.84 | 1.0 |
|  | AYSFRLFARTRSMW | 79 | 0.79 | 0.6 |
|  | KEAAAKVRFPNITN | 45 | 0.78 | 0.5 |
|  | LCFTNVYKKEILDI | 222 | 0.78 | 1.0 |
|  | TRGRKCCRRKKEAA | 35 | 0.84 | 1.0 |
|  | PTKLNDLCFTNVYK | 216 | 0.84 | 1.4 |
|  | IGKCSTRGRKCCRR | 30 | 0.83 | 1.2 |
|  | SMWSFNPKKSPTKL | 206 | 0.81 | 0.7 |
|  | LVTLAILTALGPGP | 143 | 0.81 | 0.7 |
|  | AAYYRINWITGGIA | 63 | 0.76 | 1.0 |
|  | TLACFVLAAVYRIN | 159 | 0.75 | 0.9 |
|  | NITNLCPFAAYYRI | 55 | 0.74 | 1.0 |
|  | GLLFLAFVVFLLVT | 97 | 0.67 | 0.7 |
|  | PGPGVTLACFVLAA | 154 | 0.67 | 0.7 |
|  | FARTRSMWSFGPGP | 183 | 0.66 | 0.8 |
|  | PGAFVVFLLVTLAI | 116 | 0.63 | 0.6 |
