# Supplementary material for: Reverse vaccinology assisted designing of multiepitope-based subunit vaccine against SARS-CoV-2
Source: Infect Dis Poverty. 2020 Sep 16;9:132. doi: 10.1186/s40249-020-00752-w (PMC7492789; doi:10.1186/s40249-020-00752-w)
Supplement: Supplementary file 9 — Additional file 9: Table S8. Conformational epitopes in 3D structure of vaccine. [file 40249_2020_752_MOESM9_ESM.docx]

Table S8. Conformational epitopes in 3D structure of vaccine

| Sr.No | Residues | Number of residues | Score |
| --- | --- | --- | --- |
|  | S206, _:M207, _:W208, _:S209, _:F210, _:N211, _:P212, _:K213, _:K214, _:S215, _:P216, _:T217, _:K218 | 13 | 0.936 |
|  | P249, _:V250, _:S251, _:M252, _:T253, _:K254, _:T255, _:S256, _:V257, _:D258, _:C259, _:T260, _:K261, _:K262, _:L263, _:E264, _:Q265, _:N267, _:V269 | 19 | 0.809 |
|  | G1, _:I2, _:I3, _:N4, _:T5, _:L6, _:Q7, _:K8, _:Y9, _:Y10, _:C18, _:A19, _:L21, _:S22, _:C23, _:E27, _:Q29, _:I30, _:G31, _:K32, _:C33, _:S34, _:T35, _:R36, _:G37, _:R38, _:K39, _:C40, _:R42, _:R43, _:E46, _:A47, _:A48, _:A49, _:K50, _:V51, _:R52, _:F53, _:P54, _:N55, _:I56, _:T57, _:N58, _:L59, _:C60, _:P61, _:F62, _:A63, _:Y65, _:R67, _:N69, _:I71, _:Y80, _:S81, _:F82, _:R83, _:A86, _:C238, _:S239, _:F240, _:G241, _:G242, _:V243, _:S244, _:K245, _:L273, _:L275, _:T276 | 68 | 0.67 |
|  | _  :P174, _:G175, _:P176, _:G177, _:S179, _:R181, _:F183, _:R199, _:F201, _:R203, _:T204, _:R205, _:L219, _:N220, _:D221, _:C223, _:F224, _:T225, _:N226, _:V227, _:K229 | 21 | 0.649 |
|  | _:P116, _:G117, _:A118, _:L152, _:G153, _:P154, _:G155, _:P156, _:G157, _:V158, _:A184, _:R185, _:T186, _:R187, _:S188, _:S191, _:F192, _:G193, _:P194, _:G195, _:P196, _:G197, _:F198 | 23 | 0.593 |
|  | _:G15, _:G16, _:R17, _:S90 | 4 | 0.57 |
